# Priority areas for conservation of Old World vultures

**DOI:** 10.1111/cobi.13282

**Published:** 2019-03-13

**Authors:** Andrea Santangeli, Marco Girardello, Evan Buechley, Andre Botha, Enrico Di Minin, Atte Moilanen

**Affiliations:** ^1^ The Helsinki Lab of Ornithology, Finnish Museum of Natural History University of Helsinki Helsinki FI‐00014 Finland; ^2^ Helsinki Institute of Sustainability Science University of Helsinki Helsinki FI‐00014 Finland; ^3^ cE3c ‐ Centre for Ecology Evolution and Environmental Changes/Azorean Biodiversity Group and Universidade. dos Açores – Depto de Ciências e Engenharia do Ambiente Angra do Heroísmo Açores PT‐9700‐042 Portugal; ^4^ HawkWatch International Salt Lake City UT 84106 U.S.A.; ^5^ Department of Biology University of Utah Salt Lake City UT 84112 U.S.A.; ^6^ Endangered Wildlife Trust Modderfontein 1609 South Africa; ^7^ Digital Geography Lab, Department of Geosciences and Geography University of Helsinki Helsinki FI‐00014 Finland; ^8^ School of Life Sciences University of KwaZulu‐Natal Durban 4000 South Africa; ^9^ Finnish Museum of Natural History Luomus University of Helsinki P.O. Box 17 Helsinki FI‐00014 Finland; ^10^ Department of Geosciences and Geography University of Helsinki Helsinki FI‐00014 Finland

**Keywords:** African‐Eurasian vultures, biodiversity benefits, ecosystem balance, ecosystem service, scavenger conservation, Zonation software, balance ambiental, beneficios de la biodiversidad, buitres africanos – euroasiáticos, conservación de carroñeros, servicio ambiental, software Zonation, 非洲‐欧亚的秃鹰, Zonation 软件, 食腐动物保护, 生态系统服务, 生物多样性效益, 生态系统平衡

## Abstract

The prosperity and well‐being of human societies relies on healthy ecosystems and the services they provide. However, the biodiversity crisis is undermining ecosystems services and functions. Vultures are among the most imperiled taxonomic groups on Earth, yet they have a fundamental ecosystem function. These obligate scavengers rapidly consume large amounts of carrion and human waste, a service that may aid in both disease prevention and control of mammalian scavengers, including feral dogs, which in turn threaten humans. We combined information about the distribution of all 15 vulture species found in Europe, Asia, and Africa with their threats and used detailed expert knowledge on threat intensity to prioritize critical areas for conserving vultures in Africa and Eurasia. Threats we identified included poisoning, mortality due to collision with wind energy infrastructures, and other anthropogenic activities related to human land use and influence. Areas important for vulture conservation were concentrated in southern and eastern Africa, South Asia, and the Iberian Peninsula, and over 80% of these areas were unprotected. Some vulture species required larger areas for protection than others. Finally, countries that had the largest share of all identified important priority areas for vulture conservation were those with the largest expenditures related to rabies burden (e.g., India, China, and Myanmar). Vulture populations have declined markedly in most of these countries. Restoring healthy vulture populations through targeted actions in the priority areas we identified may help restore the ecosystem services vultures provide, including sanitation and potentially prevention of diseases, such as rabies, a heavy burden afflicting fragile societies. Our findings may guide stakeholders to prioritize actions where they are needed most in order to achieve international goals for biodiversity conservation and sustainable development.

## Abstract 2

*Article impact statement*: Eighty percent of areas important for Old World vulture conservation are unprotected and in southern and eastern Africa, South Asia, and Iberia.

## Abstract (es)

Áreas Prioritarias para la Conservación de Buitres del Viejo Mundo

**Resumen**

La prosperidad y el bienestar de la sociedad humana dependen de ecosistemas sanos y de los servicios ambientales que éstos proporcionan. Sin embargo, la crisis de biodiversidad está afectando a los servicios ambientales y sus funciones. Los buitres se encuentran entre los grupos taxonómicos con mayor amenaza sobre el planeta, a pesar de tener una función fundamental en los ecosistemas. Estos carroñeros obligados consumen rápidamente grandes cantidades de carroña y desechos humanos, un servicio que puede ayudar en la prevención de enfermedades y en el control de mamíferos carroñeros, incluyendo a los perros ferales, los cuales pueden ser un peligro para los humanos. Combinamos la información sobre la distribución de las 15 especies de buitres en Europa, Asia y África con las amenazas que presentan y usamos el conocimiento detallado de expertos sobre la intensidad de las amenazas para priorizar las áreas críticas para la conservación de buitres en África y en Eurasia. Las amenazas que identificamos incluyeron el envenenamiento, la mortalidad por colisiones con infraestructura eólica y otras actividades antropogénicas relacionadas con el uso de suelo y la influencia humana. Las áreas importantes para la conservación de buitres estuvieron concentradas en el sur y el este de África, el sur de Asia y la Península Ibérica, y más del 80% de estas áreas no contaban con protección. Algunas especies de buitres requirieron áreas más grandes para su protección que otras especies. Finalmente, los países que tuvieron la mayor porción de todas las áreas prioritarias importantes e identificadas para la conservación de buitres también fueron aquellos con los mayores gastos relacionados con la carga de la rabia (por ejemplo, India, China y Myanmar). Las poblaciones de buitres han declinado marcadamente en la mayoría de estos países. La restauración de poblaciones sanas de buitres por medio de acciones enfocadas en las áreas prioritarias que identificamos puede ayudar a restaurar los servicios ambientales que proporcionan los buitres, incluyendo el saneamiento y la prevención potencial de enfermedades, como la rabia, una carga pesada que aflige a las sociedades frágiles. Nuestros resultados pueden guiar a los interesados hacia la priorización de acciones en donde más se necesitan para poder alcanzar los objetivos internacionales para la conservación de la biodiversidad y el desarrollo sustentable.

## 摘要

人类社会的繁荣昌盛依赖于健康的生态系统及其所提供的服务。然而, 生物多样性危机正在破坏生态系统的服务和功能。秃鹰是地球上最濒危的类群之一, 而它们却能提供基础的生态系统功能。它们作为专性食腐动物可以快速消耗大量腐肉及人类废弃物, 提供的生态系统服务有助于预防疾病, 以及控制哺乳类食腐动物, 如会对人类造成威胁的野狗。我们将欧洲、亚洲和非洲的全部十五种秃鹰的分布信息与其面临的威胁相结合, 利用详细的关于威胁强度的专业知识, 确定了非洲及欧亚关键的秃鹰保护优先地区。秃鹰面临的威胁包括中毒、撞击风能设施导致的死亡, 以及与人类土地利用和影响有关的其它人类活动。秃鹰的重点保护区域集中在非洲南部和东部、南非和利比亚半岛, 这些地区超过 80% 的土地没有得到保护。另外, 秃鹰中某些物种相比之下需要更大区域进行保护。我们还发现, 秃鹰的重要优先保护区域占比最大的国家同时也是那些在狂犬病上的支出最高的国家（如印度、中国、缅甸）, 而其中大多数国家的秃鹰种群数量已经明显下降。通过在我们确定的优先保护区域采取有针对性的行动来恢复健康的秃鹰种群, 可能有助于恢复秃鹰提供的生态系统服务, 包括环境卫生和预防潜在疾病, 比如狂犬病这种沉重的社会负担。我们的研究成果可以指导利益相关者在需求最迫切的地方优先采取行动, 以实现生物多样性保护和可持续发展的国际目标。**【翻译: 胡怡思; 审校: 聂永刚】**

## Introduction

The health and well‐being of human societies heavily relies on the services that healthy ecosystems provide, which in turn depend on biodiversity (Bennett et al. 2015). However, current unprecedented biodiversity loss can undermine the foundations of ecosystems resilience and associated services (Cardinale et al. 2012). The agenda for averting biodiversity decline has been internationally formalized through the Convention on Biological Diversity's 2020 Strategic Plan for Biodiversity (Aichi targets; Secretariat of the Convention on Biological Diversity 2014), which is linked to the UN 2030 Sustainable Development Goals (United Nations 2015).

Not all species are equally important in maintaining ecosystems resilience (Díaz et al. 2006), and species groups have widely different population statuses and trends (Butchart et al. 2004). As the sole obligate scavengers, vultures comprise a unique functional guild among vertebrates and play an unparalleled role in maintaining ecosystem balance (Buechley & Şekercioğlu 2016). Yet, they are among the species most threatened with extinction (Buechley & Şekercioğlu 2016; O'Bryan et al. 2018). By efficiently consuming carrion (Ogada et al. 2012), vultures may help control the spread of disease and of facultative scavenger species that can cause human injury or death, such as feral dogs (Markandya et al. 2008; Ogada et al. 2012). Vultures also play a key role in terms of waste‐disposal services and nutrient cycling (e.g., Gangoso et al. 2013; Moleón et al. 2014). Replacing these services could entail substantial costs and added greenhouse gas emissions, for example, from incineration of carcasses (Markandya et al. 2008; Morales‐Reyes et al. 2017; O'Bryan et al. 2018). Vultures are threatened by many anthropogenic drivers, such as poisons and other dietary toxins, direct persecution, collision with infrastructures and electrocution, disturbance, and habitat loss and degradation (Buechley & Şekercioğlu 2016; Botha et al. 2017). More regionally, for example, in Europe, vultures are also threatened by food shortage following sanitary regulations (Margalida & Moleón 2016) or abandonment of traditional farming practices (e.g., Olea & Mateo‐Tomás 2009). The extent of these threats and their consequences on vulture population persistence varies across the world's regions (Buechley & Şekercioğlu 2016; Ogada et al. 2016*b*; Botha et al. 2017); threats are most intense in the Old World, where most vulture species are at high risk of extinction (Buechley & Şekercioğlu 2016).

Preventing extinctions of Old World vultures is possible, as examples from Europe and Asia demonstrate (Chaudhry et al. 2012; Moreno‐Opo & Margalida 2013). However, given an accelerating decline of vulture populations (Buechley & Şekercioğlu 2016; Ogada et al. 2016b), there is an urgent need for action. Recently, a Multi‐Species Action Plan to Conserve African‐Eurasian Vultures (MSAP) has been formalized (Botha et al. 2017) that lists effective actions to conserve African‐Eurasian vultures and brings them to the top of the international conservation policy agenda. Although commitments to act and knowledge about threats have been established, limited resources impair wide‐scale implementation. Therefore, there is a need to identify priority areas for vulture conservation through conservation planning approaches (Moilanen et al. 2005). Such approaches require information about the distributions of biodiversity and associated threats; however, such information is often lacking (Joppa et al. 2016).

We combined spatially explicit data sets of relevant threats with vulture distributions to provide novel and timely insight into priority areas for vulture conservation across the Old World. We first identified priority areas where vultures and major threats, such as poisoning, wind energy infrastructure, and other human pressures, co‐occur. Second, we assessed the relationship between these priority areas and geopolitical (e.g., governance) and biodiversity conservation characteristics of the countries hosting those priorities. Governance and other national indicators are strong correlates of national investment into biodiversity conservation (Amano et al. 2017; Baynham‐Herd et al. 2018). Third, we explored the relationship between vulture priority areas identified and the national incidence of rabies mortality and associated costs. This was assessed because the decline of vultures has been linked with simultaneous increases in numbers of feral dogs and other mammalian scavengers (Markandya et al. 2008; Ogada et al. 2012) that spread diseases such as rabies. Potential linkage between loss of vultures and increased human disease burden underscores a potential synergistic opportunity for conserving vultures while helping control disease (Markandya et al. 2008; Hampson et al. 2015; O'Bryan et al. 2018).

## Methods

### Vulture Distributions

We focused on all vulture species of Africa and Eurasia, excluding the palm nut vulture (*Gypohierax angolensis*), which is not an obligate scavenger and faces different threats compared with other vultures (Buechley & Şekercioğlu 2016). Thus, we examined the 15 species that are the focus of the MSAP (Botha et al. 2017). We combined different data sources and used geostatistics to derive spatial layers of vulture distributions and threats. Details on how different spatial layers were derived are given in Supporting Information.

We extracted the resident and breeding range of each of the 15 vultures (BirdLife International and NatureServe 2015) and refined occurrence within those ranges with a statistical species distribution modeling (SDM) framework. We combined vulture occurrence data from the Global Biodiversity Information Facility and from the African Raptor DataBank from 1980 onward (Supporting Information). We filtered out duplicates and occurrences closer than 30 km from each other to minimize spatial autocorrelation (Aiello‐Lammens et al. 2015). Vulture observations were then correlated with environmental variables (e.g., climate, land‐cover, and topography [details given in Supporting Information]) with SDMs (including generalized linear models, random forest, boosted regression trees, and Maxent). Finally, we used an ensemble of the above SDMs (Urban et al. 2016) to derive consensus occurrence probabilities within the breeding and resident distribution range of each species.

### Unintentional and Intentional Poisoning

A major threat to vultures is unintentional poisoning, in which vultures are killed as a side‐effect when farmers use poison to kill carnivores following livestock depredation (Mateo‐Tomas et al. 2012; Buechley & Şekercioğlu 2016; Ogada et al. 2016b). To a lesser degree and in specific regions (e.g., Europe), poisoning may also occur when hunters attempt to regulate competitor carnivore populations (Mateo‐Tomas et al. 2012). Overall, human–carnivore conflict is a strong determinant of unintentional poisoning risk (Mateo‐Tomas et al. 2012; Santangeli et al. 2016a).

Therefore, we derived a map showing the intensity of potential human–carnivore conflict across the study region by interacting distributions of carnivores (IUCN 2017) with those of selected livestock (Robinson et al. 2014) matched by body mass (i.e., carnivores were matched with potential livestock prey species). Matching was done by searching literature for reported predation of each carnivore species on each of 3 livestock categories (poultry, including duck and chicken; small stock, including sheep, goats, and pigs; large stock, including cattle and buffalo) (Supporting Information). This literature search suggested that poultry could be predated on by any carnivore, small stock by carnivores ≥2 kg, and large stock by carnivores ≥10 kg. We then multiplied the density of each of the 3 livestock classes with the average per‐pixel (10 × 10 km grid cell) body mass of selected carnivores, resulting in 3 maps (Supporting Information). We used average body mass of selected carnivores because we were interested in a community‐weighted mean trait value of the carnivores composing a specific assembly, following, for example, Díaz et al. (2007). To obtain an overall index of human–carnivore conflict, we averaged the above 3 maps, weighted by the natural log of the body mass of each livestock class (Supporting Information). By weighting the maps by the log of the body mass of each livestock class, we aimed at assigning a higher weight to the interaction between larger stock and selected carnivores. This is associated with higher economic losses (a cow has a much higher economic value than a goat (details given in Supporting Information), implying a stronger trigger for poisoning and elevated threat intensity because larger poisoned carcasses are more likely consumed by vultures. Finally, we visually validated the resulting priority areas for vulture conservation (based on the 15 vulture species and poisoning layers) with independent data on known poisoning locations (details given in Supporting Information).

Across Sub‐Saharan Africa, a recently uncovered threat to vultures is represented by poachers intentionally poisoning vultures to eliminate their sentinel function (i.e., indicating to authorities where poaching has occurred by circling over carcasses) (Ogada et al. 2016a; Botha et al. 2017). To map this threat, we identified the herbivore and carnivore species targeted by poachers based on information on the occurrence and frequency of ungulates and carnivores found poisoned from 2007 onward (Endangered Wildlife Trust and the Peregrine Fund 2017). This search suggested that the carcasses of medium‐ to large‐sized herbivores (e.g., body mass of 53 kg for impala [*Aepyceros melampus*] and above) and 2 carnivores (lion [*Panthera leo*] and leopard [*Panthera pardus*]) are poisoned to intentionally kill vultures; some species were targeted more often than others. Because not all poisoning incidences have been detected and reported in the database, we conservatively selected all Cetartiodactyla and Perissodactyla species of Africa with body mass ≥ 20 kg and the African elephant (*Loxodonta africana*), lion, and leopard (Ogada et al. 2016a). This resulted in 72 species, to each of which we assigned a conservative weight based on the log of the frequency of its occurrence in the African wildlife poisoning database (Supporting Information). Thus, species that are more often reported in the database had a comparatively higher weight in driving the intentional poisoning map compared with species reported once or never (the latter 2 equally received the lowest weight) (Supporting Information). We then used the International Union for Conservation of Nature (IUCN 2017) range maps of the 72 target species of intentional poisoning and the species‐specific weight to derive a single map of sentinel poisoning risk. In doing so, we calculated the average of the species‐specific weight of intentional poisoning across all species present in each 10 × 10 km pixel within the range of the study area in Africa (Supporting Information).

### Wind Collision Risk

We used Pogson et al.’s (2013) map of wind‐power potential as a proxy for exposure to collision with wind turbines, a major threat to vultures (Pearce‐Higgins & Green 2014; Ogada et al. 2016b; Botha et al. 2017). This map approximates installed and planned national wind energy capacity (REN21 2017; Santangeli et al. 2018) and has been used to evaluate impacts of wind energy expansion on biodiversity (Santangeli et al. 2016b, 2016c, 2018).

### Human Influence Index

Collision with and electrocution on energy infrastructure, human disturbance, habitat degradation, and decline in food availability are further important threats to vultures (Botha et al. 2017). These 5 threats are all linked to human influence and were captured by a single proxy layer: the Global Human Influence Index (GHII) (Wildlife Conservation Society and Center for International Earth Science Information Network 2005). The GHII combines information on human population pressure, land use, infrastructure, and accessibility (details given in Supporting Information).

### Treatment of Expert Knowledge

The priority of each of the above threats to vultures varies regionally and has been recently quantified as part of the MSAP by experts using standardized classes of critical, high, and medium or low priority (Botha et al. 2017). We incorporated this information by assigning a weight of 2 to critical threats, 1.5 to high threats, and 1 to medium to low threats by region (Botha et al. 2017) (Supporting Information). We then refined the spatial threat layers by multiplying their original values with the assigned threat‐specific regional weight (Supporting Information). Because GHII represents a proxy for 5 threats, the sum of weights of the 5 threats per region was used. The resulting 4 spatial layers would ultimately represent the spatial distribution of threats, weighted by priority, as assessed by experts (Supporting Information).

### Spatial Prioritization Analyses

We used the software Zonation version 4.0 (Moilanen et al. 2014) to identify priority areas for vulture conservation across Africa and Eurasia. Heuristically expressed, we sought to identify areas where many vultures occur and threats are most intense. It is important that the threats incorporated here can be reduced with specific actions (e.g., Woodroffe et al. 2005; Northrup & Wittemyer 2013; Botha et al. 2017; Murn & Botha 2018). Zonation develops spatial priority rankings based on the distributions of biodiversity features (e.g., vultures) and, optionally, on threats or costs. It is an important characteristic of Zonation that complementarity is accounted for (i.e., a balanced representation across all features is maintained through the priority ranking) (Moilanen et al. 2014).

We used the core area method (CAZ; Moilanen et al. 2005) as the ranking method, with the interpretation that high‐priority areas should include locally high‐quality areas for all species individually (Moilanen et al. 2005). All feature layers (vulture distributions and their threats) were rasterized to the resolution of 10 × 10 km, and each threat layer was rescaled to between 0 and 1 prior to analyses. The adopted resolution was deemed most appropriate given the available distribution data on vultures and their threats. Commission errors (i.e., predicting presence incorrectly) are minimized for the vulture distributions because these species’ ranges are well known and because they have been refined here with a robust SDM approach (details given in Supporting Information). Similarly, the underlying data used to derive the threat layers had a resolution of 10 × 10 km or higher or an appropriate original resolution given that used here (Montesino Pouzols et al. 2014). In all analyses, each vulture species was given a specific weight based on its IUCN Red List status following Di Minin et al. (2016) (weights: 1, least concern; 2, near threatened; 3, vulnerable, 4, endangered, 5, critically endangered; none, data deficient).

**Figure 1:**
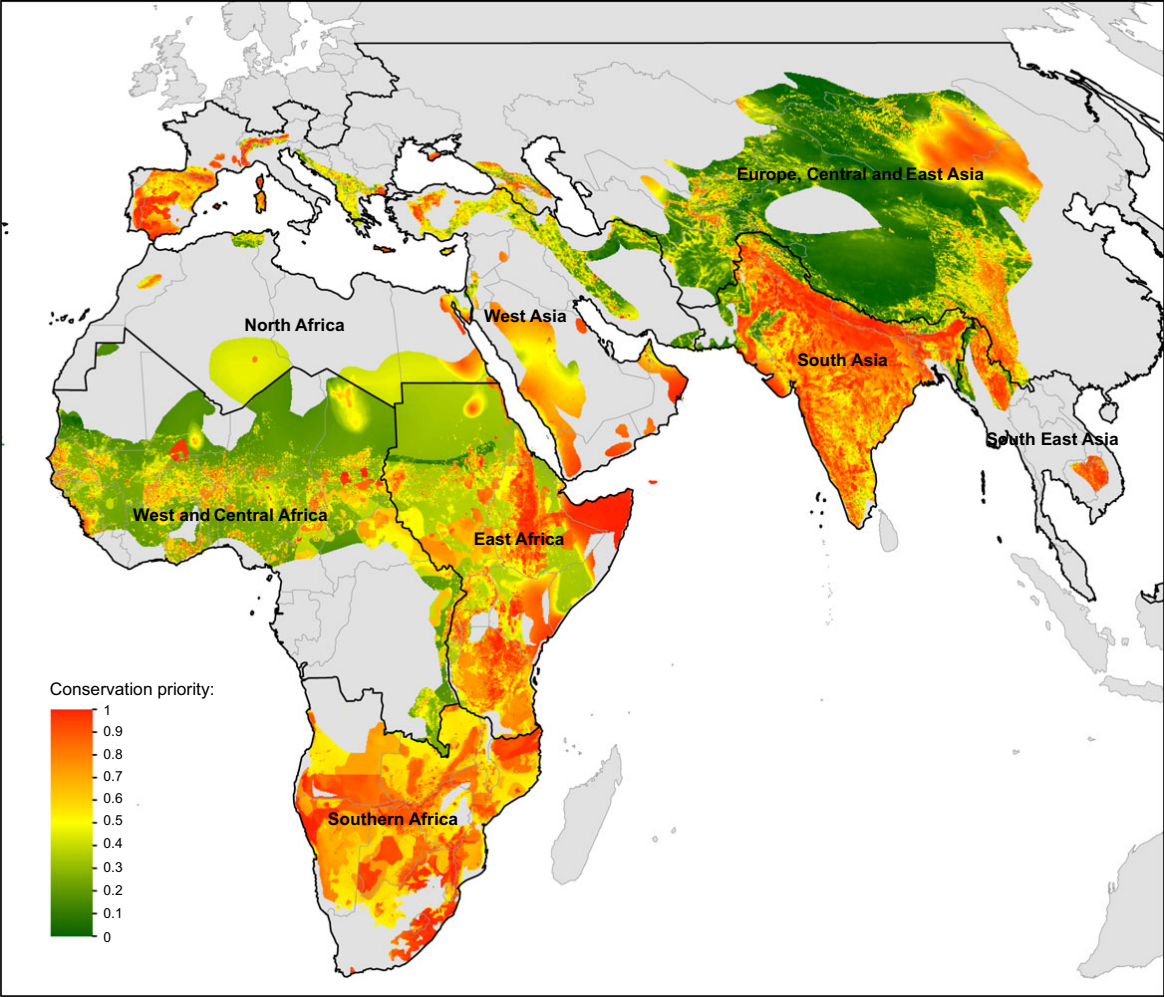
Priority areas for vulture conservation identified through spatial conservation prioritization across Africa and Eurasia (gray, outside breeding and resident range of any of the 15 vultures considered). Priorities are ranked from highest (red) to lowest (green). The abrupt shift in priorities across some country borders is due to the different weights assigned to threats within 8 subregions (black lines) based on expert knowledge (Supporting Information).

We first ran 4 separate prioritization analyses, each including the distributions of the 15 vultures and with each of the 3 threats included in turn: unintentional and intentional poisoning together, wind energy, and GHII. Next, we ran a holistic scenario in which all 3 threats and the 15 vultures were included. For all scenarios, threat layers were weighted so that their aggregate weight equaled the aggregate weight of all vulture species, following Montesino Pouzols et al. (2014). This ensured a balanced representation between areas important for vulture conservation and areas where threats are most intense. These prioritization analyses deliver maps showing the priority areas for vulture conservation where threats are most intense. We further extracted the 30% of priority areas most important (i.e., all pixels with ranked value >0.7 [Fig. 1]) (rationale below) for vulture conservation (hereafter, vulture priority areas) (rationale discussed below), converted them into vector files, and calculated the area of overlap with the global network of protected areas (PAs) (i.e., IUCN protected‐area categories I–VI obtained from UNEP‐WCMC and IUCN [2017]) and important bird and biodiversity areas (IBAs) (BirdLife International 2014). We also identified the number of IBAs in danger (i.e., under high pressure in 2018) (BirdLife International 2014) that at least partly overlapped with the above vulture priority areas. Robustness to the uncertainty associated with the species distributions and selected threat layers, as well as to changes in the relative weight assigned to each feature, was assessed by means of sensitivity analyses (details given in Supporting Information).

### National‐Level Analyses

We calculated the national share of the vulture priority areas (pixels with rank > 0.7) (Fig. 1) following Butchart et al. (2015), Santangeli et al. (2018), and Santangeli et al. (2016b). Although the top 30% threshold used here is somewhat arbitrary, it represents a balanced trade‐off between allowing enough protection for vulture ranges and the reality of resource limitations and societal constraints on implementation. We used this value as the response in a beta regression model (because the response is a percentage) aiming to quantify its relationship with national socioeconomic, governance, and environmental indicators (Di Minin et al. 2016; Amano et al. 2017; Baynham‐Herd et al. 2018), as well as national incidence of and costs related to rabies (Hampson et al. 2015) (Supporting Information). Prior to analyses, we log transformed and rescaled all predictor variables. A variance inflation analysis (VIF) run on the set of predictors indicated that country size and human development index had high VIF values (i.e., > 3) and were therefore excluded. The remaining predictors (governance, percent terrestrial PAs, rabies costs, and incidences) were largely uncorrelated (VIF < 2). We applied multimodel selection and averaging based on the best models (with ∆AIC < 4) (Burnham & Anderson 2002) to quantify the relative importance and relationship of each predictor with the response variable with the package MuMIn in R version 3.0.3 (Bartoń 2014). Model validation was performed by inspecting the residuals. There was no sign of violation of model assumptions. We repeated the above analyses with national share of vulture priority areas as the response variable, calculated by considering in turn the top 20% and 40% of priority areas.

## Results

**Figure 2:**
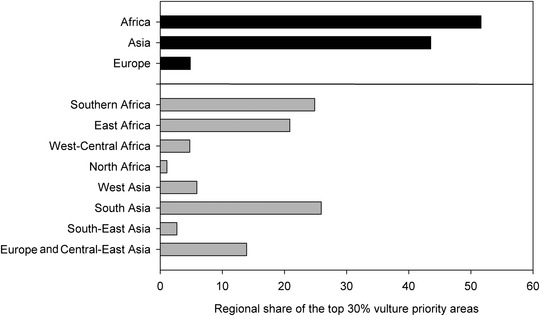
Continental (black) and regional (gray) share of the 30% of priority areas most important for vulture conservation (red areas in Fig. 1). Geographic regions considered are those defined in the Multi‐Species Action Plan to Conserve African‐Eurasian Vultures (Botha et al. 2017) (Supporting Information).

**Figure 3:**
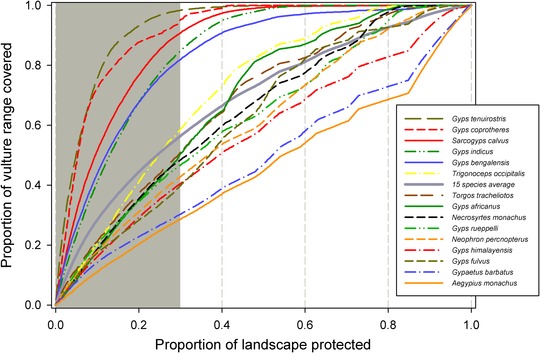
Zonation performance curves showing the relation between conservation coverage of the range of each vulture species (y‐axis: 1, all species’ ranges protected) and hypothetical proportions of the landscape protected for vultures (x‐axis: 1, entire study area protected) (gray, 30% of priority areas most important for vulture conservation). Species‐specific conservation coverage can be determined from the y‐axis, where the rightmost edge of the gray area (*x* = 0.3) intersects the species‐specific performance curve.

The prioritization analysis indicated the highest priority areas for vulture conservation across the Old World were concentrated in southern and eastern Africa, southern Europe, the Arabian Peninsula, and the Indian subcontinent (Fig. 1). About 95% of the top‐ranked 30% priority areas for vulture conservation in the region fell within Africa (51.6%) and Asia (43.5%), and within these southern Africa, East Africa, and South Asia each supported over 20% of the top 30% ranked areas for vulture conservation (Fig. 2). The high‐priority areas for vulture conservation as shown in Figure 1 are typically affected by multiple threats, particularly poisoning and other threats associated with human influence (Supporting Information). The above results were robust (i.e., had low sensitivity to the uncertainty associated with the vulture distributions and selected threats or to changes in the weight assigned to each feature) (Supporting Information). About one‐fifth of the top 30% vulture priority areas shown in Figure 1 were covered by the global PA network (19%), whereas 12% of them were covered by IBAs. Moreover, 38 of the IBAs overlapping vulture priority areas had been classified by BirdLife International as being in danger, largely owing to high pressures to develop these areas. Species‐specific performance curves showed the increase in (conservation) coverage of each vulture species' distribution as a function of area selected (Fig. 3). These curves clearly indicated that protection efforts will not be similarly effective for all species. Some species, such as *Gyps tenuirostris* and *Gyps coprotheres*, would reach almost full range coverage if just 30% of the landscape were protected. Others, such as *Aegypius monachus* and *Gypaetus barbatus*, would require a much larger fraction of land to be protected for their ranges to be adequately covered (Fig. 3). These differences between species were explained by different distribution sizes and overlaps of species distribution: small overlapping ranges can be protected much easier than large nonoverlapping ranges.

**Figure 4:**
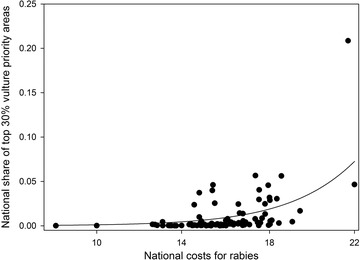
Relationship between proportion of national share of vulture priority areas (i.e., the 30% of priority areas most important for vulture conservation; red areas in Fig. 1) and the total national costs related to the burden of rabies ($US in 2010 per year on a log scale; dots, separate countries; black line, relationship as predicted by the beta regression model [see Table 1 and Methods]).

We found that countries harboring the highest share of vulture priority areas were those that incurred the highest economic costs, but not human mortality incidences, associated with the burden of rabies (Fig. 4), and those with good governance (Table 1). Analyses based on the top 20% and 40% priority areas confirmed the above results.

**Table 1:** Fit of a beta regression model quantifying the relationship between national share of the 30% of priority areas most important for vulture conservation (red areas in Fig. 1) and 5 national‐level variables (predictors described in Supporting Information)

Variable	*β*	SE	*Z*	*p*
Intercept	−4.69	0.14	32.46	<0.001
Governance	0.24	0.11	2.13	0.033
Terrestrial protected areas (%)	0.12	0.11	1.10	0.273
Rabies costs	0.72	0.10	7.16	<0.001
Rabies incidence	0.03	0.11	0.28	0.782

## Discussion

By combining vulture distributions with their threats and using regional expert knowledge on threat intensity, we produced the first holistic map of priority areas for vulture conservation across Africa and Eurasia. We found that high‐priority areas were mostly concentrated in southern and eastern Africa, South Asia, and the Iberian Peninsula. These areas were largely unprotected. We found major differences in the species‐specific performance curves, highlighting that some species would require larger areas for protection than others. Finally, we showed that countries holding the largest share of priority areas for vulture conservation were those that also paid the highest costs from rabies burden (such as India, China, and Myanmar).

Priority areas for vulture conservation identified here represented large areas that were clustered within specific regions and countries. Although this aggregation of priorities may represent a challenge due to the disproportionate responsibility of some countries toward vulture conservation, it may also present an opportunity, due to the limited number of countries and stakeholders involved. Actions to revert the major threats to vultures are broadly known (Buechley & Şekercioğlu 2016; Botha et al. 2017). Although some of these actions, such as rapid response interventions on poisoning events and supplementary feeding stations, may reduce adverse impacts from threats such as poisoning (Cortes‐Avizanda et al. 2016; Murn & Botha 2018), these are short‐term solutions only. Fundamentally, actions of wide temporal and spatial scope and impact are needed. Among these, design and strong enforcement of targeted legislation would help restrict the distribution and use of drugs and poisons that threaten vultures (Ogada 2014). Similarly, strict regulations to ensure proper environmental impact assessments and planning would help reduce risks from wind energy and other infrastructure development in areas important for vulture conservation. For regionally localized threats, locally targeted measures could play a key role. For example, the Protection Areas for the Feeding of Necrophagous Species of European Interest (EC 142/2011) program in the Iberian Peninsula allows farmers to leave livestock carcasses in the field, providing food for vultures (Morales‐Reyes et al. 2017). This practice was banned following bovine spongiform encephalopathy in Europe. Ultimately, it will be important to identify the relevant local stakeholders, such as communities, nongovernmental organizations, government institutions, private, and state‐owned companies, and address the threats with a participatory, community‐engaged approach.

Our results also highlight that restricting conservation efforts toward vulture priority areas as identified here (Fig. 1) may be effective for some species but inadequate for others (Fig. 3). Species with a restricted distribution, such as *G. tenuirostris* and *G. coprotheres*, would benefit from actions that target threats inside the vulture priority areas. Conversely, widespread species, such as *G. barbatus* and *A. monachus*, will require action across very large areas.

We found a positive relationship between national governance and the share of vulture priority areas. This indicates that vulture priority areas are more concentrated in countries with good governance. National governments can play a key role for vulture conservation, as the Asian case indicates (e.g., Prakash et al. 2012). Overall, governance levels may drive the cost‐effectiveness of conservation (Amano et al. 2017), and the potential implications of this should be considered when defining actions targeting vulture priority areas. To this end, local and national stakeholder participation will be key to designing national‐ and regional‐level actions that will be feasible to implement and cost‐effective under each specific context, governance level included.

We found correlative evidence that countries investing large financial resources to counter rabies also held the largest shares of vulture priority areas. In many of these areas, vulture populations have declined markedly over the past decades, leading to a loss of their waste removal and potential disease regulating services (Pain et al. 2008; Ogada et al. 2012; Buechley & Şekercioğlu 2016). By rapidly consuming carrion, vultures reduce access to carrion and direct contact between mammalian scavengers (Ogada et al. 2012) and thus have been theorized to help mitigate the spread of diseases such as rabies (Markandya et al. 2008). Spread of rabies leads to increased livestock and human mortality and increased public health expenditure, causing high rabies‐related economic costs in Asia and Africa (Hampson et al. 2015). The positive correlation between national economic spending for rabies and vulture priority areas that we found may highlight a potential opportunity for restoring vulture populations that could simultaneously result in national savings from reduced rabies burden. However, the extent and value of potential disease control provided by vultures needs to be better demonstrated with empirical evidence (O'Bryan et al. 2018). At present, this evidence is largely correlative and localized, thereby preventing firm conclusions about the link (or lack thereof) between vultures and disease, including rabies. Localized evidence is available, for example, from a study in India, which reported that when the national vulture populations dropped by 99% from 1992 to 2003, feral dog numbers increased and so did cases of human dog bites and rabies (Markandya et al. 2008). This occurred despite widespread and very expensive dog sterilization programs over the period (Markandya et al. 2008).

Although there has been recent progress to bring vulture conservation to the top of the international conservation science and policy agenda (Botha et al. 2017), there is now an urgent need to mobilize funds and implement action. Our findings can help guide direct action where it is needed most. Saving vultures is not only a matter of conservation ethics and principle, but also about saving a unique functional guild that provides key ecosystem services (Markandya et al. 2008; Buechley & Şekercioğlu 2016). No other functional guild is dominated by a group of so few and yet so endangered species.

## Supporting information

Additional methods (Appendix S1); count of vulture occurrence data (Appendix S2); list of land‐cover categories (Appendix S3); SDM model performance (Appendix S4); list of carnivore species and attributes (Appendix S5); interactions of large stock, small stock, and poultry with carnivores (Appendix S6–8); livestock–carnivore interactions (Appendix S9); validation of poisoning layer (Appendix S10); list of species used for intentional poisoning layer (Appendix S11); intentional poisoning layer (Appendix S12); threat intensity by region (Appendix S13); threat weights by region (Appendix S14); description of national predictors (Appendix S15); priority areas from intermediate scenarios (Appendix S16); validation priority areas (Appendix S17); and sensitivity of priorities to weight changes (Appendix S18) are available online. The authors are solely responsible for the content and functionality of these materials. Queries (other than absence of the material) should be directed to the corresponding author. Priority maps of the holistic scenario and alternative intermediate scenarios are available from https://vultureconservation.shinyapps.io/vulturepriorities/.

Supporting Information

Supporting Information

Supporting Information

## References

[cobi13282-bib-0001] Aiello‐Lammens ME , Boria RA , Radosavljevic A , Vilela B , Anderson RP . 2015 spThin: an R package for spatial thinning of species occurrence records for use in ecological niche models. Ecography 38:541–545.

[cobi13282-bib-0002] Amano T , Székely T , Sandel B , Nagy S , Mundkur T , Langendoen T , Blanco D , Soykan CU , Sutherland WJ . 2017 Successful conservation of global waterbird populations depends on effective governance. Nature 553:199. 29258291 10.1038/nature25139

[cobi13282-bib-0003] Bartoń K . 2014 Package “MuMIn” — multi‐model interence. Available from https://CRAN.R-project.org/package=MuMIn (accessed November 2017).

[cobi13282-bib-0004] Baynham‐Herd Z , Amano T , Sutherland WJ , Donald PF . 2018 Governance explains variation in national responses to the biodiversity crisis. Environmental Conservation 45:407–418.

[cobi13282-bib-0005] Bennett EM , et al. 2015 Linking biodiversity, ecosystem services, and human well‐being: three challenges for designing research for sustainability. Current Opinion in Environmental Sustainability 14:76–85.

[cobi13282-bib-0006] BirdLife International . 2014 Important bird and biodiversity areas: a global network for conserving nature and benefiting people. BirdLife International, Cambridge, United Kingdom. Available from http://datazone.birdlife.org/userfiles/file/IBAs (accessed November 2017).

[cobi13282-bib-0007] BirdLife International and NatureServe . 2015 Bird species distribution maps of the world. Version 4.0. BirdLife International, Cambridge, United Kingdom, and NatureServe, Arlington, Virginia.

[cobi13282-bib-0008] Botha A , Andevski J , Bowden CGR , Gudka M , Safford RJ , Tavares J , Williams NP . 2017 Multi‐species Action Plan to Conserve African‐Eurasian Vultures. Coordinating Unit of the CMS Raptors MOU. CMS raptors MOU technical publication 5. United Nations Environment Programme, Abu Dhabi, United Arab Emirates.

[cobi13282-bib-0009] Buechley ER , Şekercioğlu ÇH . 2016 The avian scavenger crisis: looming extinctions, trophic cascades, and loss of critical ecosystem functions. Biological Conservation 198:220–228.

[cobi13282-bib-0010] Burnham KP , Anderson DR . 2002 Model selection and multimodel inference: a practical information‐theoretic approach. Springer, New York.

[cobi13282-bib-0011] Butchart SHM , et al. 2015 Shortfalls and solutions for meeting national and global conservation area targets. Conservation Letters 8:329–337.

[cobi13282-bib-0012] Butchart SHM , Stattersfield AJ , Bennun LA , Shutes SM , Akcakaya HR , Baillie JEM , Stuart SN , Hilton‐Taylor C , Mace GM . 2004 Measuring global trends in the status of biodiversity: red list indices for birds. PLOS Biology 10.1371/journal.pbio.0020383. PMC524254 15510230

[cobi13282-bib-0013] Cardinale BJ , et al. 2012 Biodiversity loss and its impact on humanity. Nature 486:59. 22678280 10.1038/nature11148

[cobi13282-bib-0014] Chaudhry MJI , Ogada DL , Malik RN , Virani MZ , Giovanni MD . 2012 First evidence that populations of the critically endangered Long‐billed Vulture *Gyps indicus* in Pakistan have increased following the ban of the toxic veterinary drug diclofenac in south Asia. Bird Conservation International 22:389–397.

[cobi13282-bib-0015] Cortes‐Avizanda A , Blanco G , DeVault TL , Markandya A , Virani MZ , Brandt J , Donazar JA . 2016 Supplementary feeding and endangered avian scavengers: benefits, caveats, and controversies. Frontiers in Ecology and the Environment 14:191–199.

[cobi13282-bib-0016] Di Minin E , Slotow R , Hunter LTB , Montesino Pouzols F , Toivonen T , Verburg PH , Leader‐Williams N , Petracca L , Moilanen A . 2016 Global priorities for national carnivore conservation under land use change. Scientific Reports 10.1038/srep23814. PMC4817124 27034197

[cobi13282-bib-0017] Díaz S , Fargione J , Chapin FS, III , Tilman D . 2006 Biodiversity loss threatens human well‐being. PLOS Biology 10.1371/journal.pbio.0040277. PMC1543691 16895442

[cobi13282-bib-0018] Díaz S , Lavorel S , de Bello F , Quétier F , Grigulis K , Robson TM . 2007 Incorporating plant functional diversity effects in ecosystem service assessments. Proceedings of the National Academy of Sciences of the United States of America 104:20684–20689. 18093933 10.1073/pnas.0704716104 PMC2410063

[cobi13282-bib-0019] Endangered Wildlife Trust and the Peregrine Fund. 2017 African wildlife poisoning database. Available from http://www.africanwildlifepoisoning.org (accessed November 2017).

[cobi13282-bib-0020] Gangoso L , Agudo R , Anadón JD , Riva Mdela , Suleyman AS , Porter R , Donázar JA . 2013 Reinventing mutualism between humans and wild fauna: insights from vultures as ecosystem services providers. Conservation Letters 6:172–179.

[cobi13282-bib-0021] Hampson K , et al. 2015 Estimating the global burden of endemic canine rabies. PLOS Neglected Tropical Diseases 10.1371/journal.pntd.0003709. PMC4400070 25881058

[cobi13282-bib-0022] IUCN (International Union for Conservation of Nature) . 2017 The IUCN Red List of threatened species. IUCN, Gland, Switzerland. Available from: http://www.iucnredlist.org (accessed April 2017).

[cobi13282-bib-0023] Joppa LN , et al. 2016 Filling in biodiversity threat gaps. Science 352:416–418. 27102469 10.1126/science.aaf3565

[cobi13282-bib-0024] Margalida A , Moleón M . 2016 Toward carrion‐free ecosystems? Frontiers in Ecology and the Environment 14:183–184.

[cobi13282-bib-0025] Markandya A , Taylor T , Longo A , Murty MN , Murty S , Dhavala K . 2008 Counting the cost of vulture decline — an appraisal of the human health and other benefits of vultures in India. Ecological Economics 67:194–204.

[cobi13282-bib-0026] Mateo‐Tomas P , Olea PP , Sanchez‐Barbudo IS , Mateo R . 2012 Alleviating human–wildlife conflicts: identifying the causes and mapping the risk of illegal poisoning of wild fauna. Journal of Applied Ecology 49:376–385.

[cobi13282-bib-0027] Moilanen A , Franco AMA , Early RI , Fox R , Wintle B , Thomas CD . 2005 Prioritizing multiple‐use landscapes for conservation: methods for large multi‐species planning problems. Proceedings of the Royal Society B: Biological Sciences 272:1885–1891. 10.1098/rspb.2005.3164 PMC1559892 16191593

[cobi13282-bib-0028] Moilanen A , Pouzols FM , Meller L , Veach V , Arponen A , Leppänen J , Kujala H . 2014 Zonation version 4 user manual. C‐BIG Conservation Biology Informatics Group, Department of Biosciences, University of Helsinki, Helsinki.

[cobi13282-bib-0029] Moleón M , Sánchez‐Zapata JA , Margalida A , Carrete M , Owen‐Smith N , Donázar JA . 2014 Humans and scavengers: the evolution of interactions and ecosystem services. BioScience 64:394–403.

[cobi13282-bib-0030] Montesino Pouzols F , et al. 2014 Global protected area expansion is compromised by projected land‐use and parochialism. Nature 516:383–386. 25494203 10.1038/nature14032

[cobi13282-bib-0031] Morales‐Reyes Z , et al. 2017 Evaluation of the network of protection areas for the feeding of scavengers in Spain: from biodiversity conservation to greenhouse gas emission savings. Journal of Applied Ecology 54:1120–1129.

[cobi13282-bib-0032] Moreno‐Opo R , Margalida A . 2013 Conservation of the Cinereous Vulture *Aegypius monachus* in Spain (1966–2011): a bibliometric review of threats, research and adaptive management. Bird Conservation International 24:178–191.

[cobi13282-bib-0033] Murn C , Botha A . 2018 A clear and present danger: impacts of poisoning on a vulture population and the effect of poison response activities. Oryx 52:552–558.

[cobi13282-bib-0034] Northrup JM , Wittemyer G . 2013 Characterising the impacts of emerging energy development on wildlife, with an eye towards mitigation. Ecology Letters 16:112–125. 23013218 10.1111/ele.12009

[cobi13282-bib-0035] O'Bryan CJ , Braczkowski AR , Beyer HL , Carter NH , Watson JEM , McDonald‐Madden E . 2018 The contribution of predators and scavengers to human well‐being. Nature Ecology & Evolution 2:229–236. 29348647 10.1038/s41559-017-0421-2

[cobi13282-bib-0036] Ogada D , Botha A , Shaw P . 2016a Ivory poachers and poison: drivers of Africa's declining vulture populations. Oryx 50:593–596.

[cobi13282-bib-0037] Ogada D , et al. 2016b Another continental vulture crisis: Africa's vultures collapsing toward extinction. Conservation Letters 9:89–97.

[cobi13282-bib-0038] Ogada DL . 2014 The power of poison: pesticide poisoning of Africa's wildlife. Annals of the New York Academy of Sciences 1322:1–20. 24716788 10.1111/nyas.12405

[cobi13282-bib-0039] Ogada DL , Torchin ME , Kinnaird MF , Ezenwa VO . 2012 Effects of vulture declines on facultative scavengers and potential implications for mammalian disease transmission. Conservation Biology 26:453–460. 22443166 10.1111/j.1523-1739.2012.01827.x

[cobi13282-bib-0040] Olea PP , Mateo‐Tomás P . 2009 The role of traditional farming practices in ecosystem conservation: the case of transhumance and vultures. Biological Conservation 142:1844–1853.

[cobi13282-bib-0041] Pain DJ , et al. 2008 The race to prevent the extinction of South Asian vultures. Bird Conservation International 18:S30–S48.

[cobi13282-bib-0042] Pearce‐Higgins JW , Green RE . 2014 Birds and climate change — impacts and conservation responses. Cambridge University Press, Cambridge, United Kingdom.

[cobi13282-bib-0043] Pogson M , Hastings A , Smith P . 2013 How does bioenergy compare with other land‐based renewable energy sources globally? Global Change Biology Bioenergy 5:513–524.

[cobi13282-bib-0044] Prakash V , et al. 2012 The population decline of *Gyps* vultures in India and Nepal has slowed since veterinary use of diclofenac was banned. PLOS ONE (e49118) 10.1371/journal.pone.0049118. PMC3492300 23145090

[cobi13282-bib-0045] REN21 . 2017 Renewables 2017 global status report in R. REN21 Secretariat, Paris.

[cobi13282-bib-0046] Robinson TP , Wint GRW , Conchedda G , Van Boeckel TP , Ercoli V , Palamara E , Cinardi G , D'Aietti L , Hay SI , Gilbert M . 2014 Mapping the global distribution of livestock. PLOS ONE (e96084) 10.1371/journal.pone.0096084. PMC4038494 24875496

[cobi13282-bib-0047] Santangeli A , Arkumarev V , Rust N , Girardello M . 2016a Understanding, quantifying and mapping the use of poison by commercial farmers in Namibia – implications for scavengers' conservation and ecosystem health. Biological Conservation 204:205–211.

[cobi13282-bib-0048] Santangeli A , Butchart SHM , Pogson M , Hastings A , Smith P , Girardello M , Moilanen A . 2018 Mapping the global potential exposure of soaring birds to terrestrial wind energy expansion. Ornis Fennica 95:1–14.

[cobi13282-bib-0049] Santangeli A , Di Minin E , Toivonen T , Pogson M , Hastings A , Smith P , Moilanen A . 2016b Synergies and trade‐offs between renewable energy expansion and biodiversity conservation — a cross‐national multifactor analysis. Global Change Biology Bioenergy 8:1191–1200.

[cobi13282-bib-0054] Santangeli A , Toivonen T , Pouzols FM , Pogson M , Hastings A , Smith P , Moilanen A . 2016c Global change synergies and trade‐offs between renewable energy and biodiversity. Global Change Biology Bioenergy 8:941–951.

[cobi13282-bib-0050] UNEP (UN Environment Programme)‐WCMC (World Conservation Monitoring Centre) and International Union for Conservation of Nature (IUCN) . 2017 Protected planet: the world database on protected areas. UNEP, Nairobi. Available from http://www.protectedplanet.net (accessed August 2017).

[cobi13282-bib-0051] United Nations (UN) . 2015 Transforming our world: the 2030 agenda for sustainable development. UN General Assembly, New York.

[cobi13282-bib-0055] Urban MC , et al. 2016 Improving the forecast for biodiversity under climate change. Science 353 10.1126/science.aad8466. 27609898

[cobi13282-bib-0052] Wildlife Conservation Society (WCS) and Center for International Earth Science Information Network. 2005 Last of the wild project, version 2, global human footprint dataset (geographic). WCS, New York. Available from 10.7927/H4M61H5F (accessed April 2017).

[cobi13282-bib-0053] Woodroffe R, Thirgood SJ, Rabinowitz A. 2005 People and wildlife: Conflict or co‐existence? Cambridge University Press, Cambridge, United Kingdom.

