# Supplementary material for: Priority areas for conservation of Old World vultures
Source: Conserv Biol. 2019 Mar 13;33(5):1056–65. doi: 10.1111/cobi.13282 (PMC6849836; doi:10.1111/cobi.13282)
Supplement: Supplementary file 1 — Additional methods (Appendix S1); count of vulture occurrence data (Appendix S2); list of land‐cover categories (Appendix S3); SDM model performance (Appendix S4); list of carnivore species and attributes (Appendix S5); interactions of large stock, small stock, and poultry with carnivores (Appendix S6–8); livestock–carnivore interactions (Appendix S9); validation of poisoning layer (Appendix S10); list of species used for intentional poisoning layer (Appendix S11); intentional poisoning layer (Appendix S12); threat intensity by region (Appendix S13); threat weights by region (Appendix S14); description of national predictors (Appendix S15); priority areas from intermediate scenarios (Appendix S16); validation priority areas (Appendix S17); and sensitivity of priorities to weight changes (Appendix S18) are available online. The authors are solely responsible for the content and functionality of these materials. Queries (other than absence of the material) should be directed to the corresponding author. Priority maps of the holistic scenario and alternative intermediate scenarios are available from https://vultureconservation.shinyapps.io/vulturepriorities/. [file COBI-33-1056-s001.docx]

**Supporting Material Figures:**


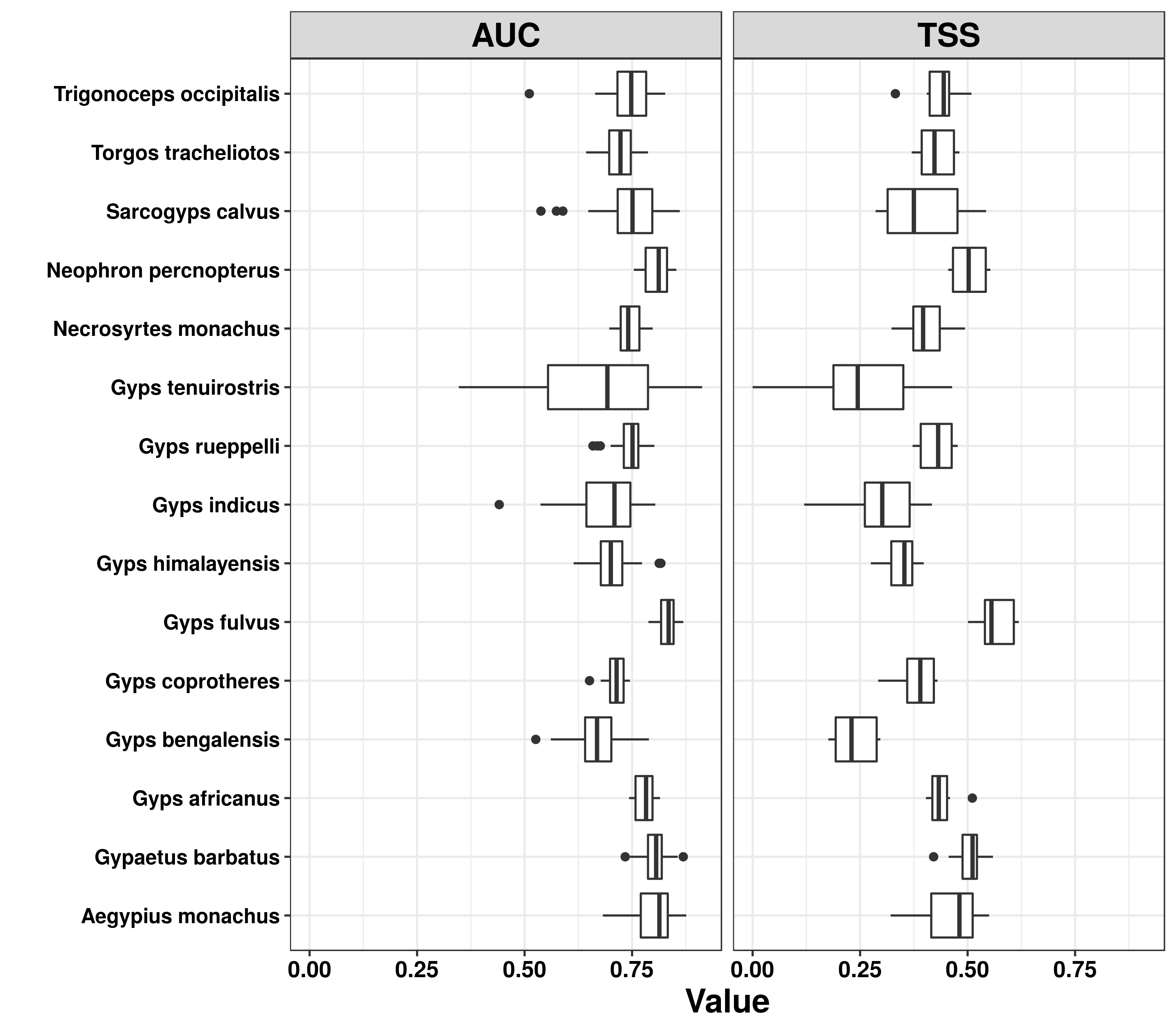


**Figure S1.** Validation metrics for the species distribution models (SDM) for each of the 15 vulture species considered for this study. The figure shows values for the Area Under the receiver operating characteristic Curve (AUC; values of AUC > 0.70 indicate a fair discriminatory capacity according to the guidelines proposed by Swets (1988)) and the maximum True Skill Statistic (TSS; values of TSS > 0.4 indicate good discriminatory capacity) for the SDM of each vulture species used for the prioritization analysis. Only models with a fair performance according the ROC values were retained to create the final ensemble (AUC > 0.70). These were chosen on the basis of the guidelines provided by Swets 1988 (Measuring the accuracy of diagnostic systems. *Science*, *240*(4857), 1285-1293).


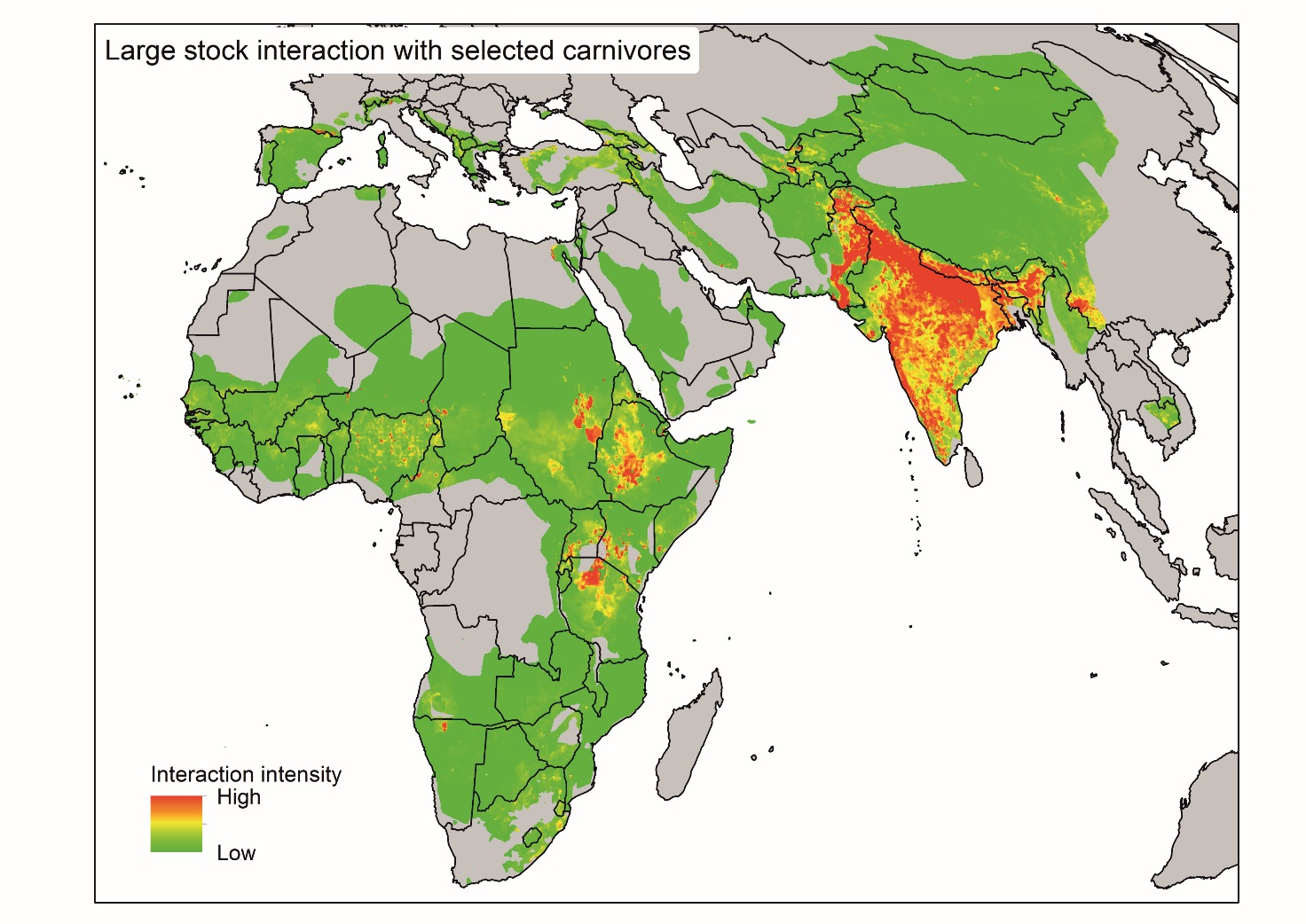


**Figure S2.** Map showing the interaction between density of large stock (i.e. cattle and buffalo) and the average body mass of selected carnivores (see Table S3). The interaction was performed by means of multiplication of the values of large stock density and average value for the selected carnivore body mass (see extended methods Appendix 1 for further details). Areas with color towards red represent high values for the interaction, whereby high densities of large stock co-occur with selected carnivores of high average body mass. These areas are intended as proxies for high potential conflict between large livestock farming and carnivores as opposed to the green areas in the map. Areas beyond the range of any of the 15 vulture species focus of this study are shown in grey.


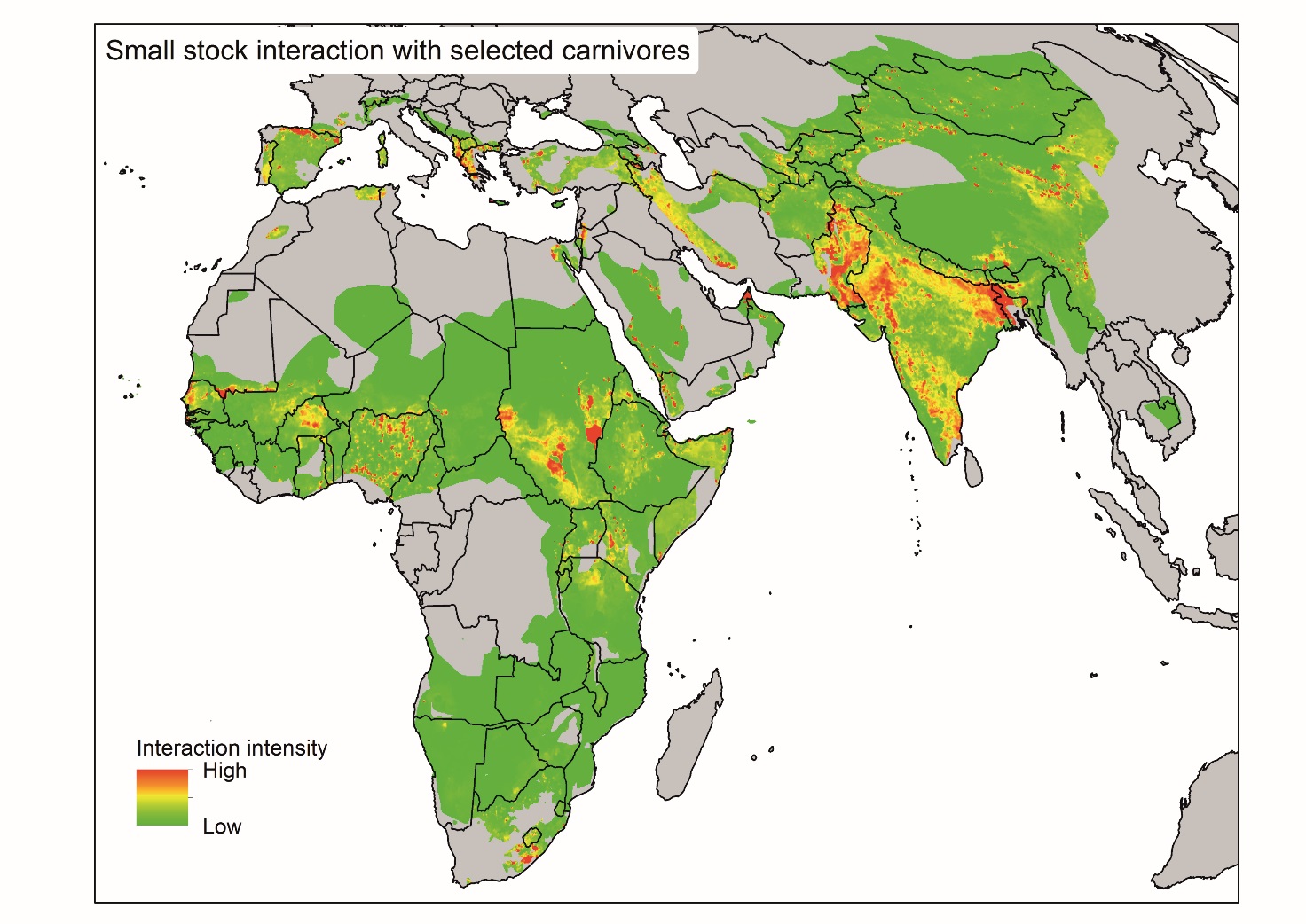


**Figure S3.** Map showing the interaction between density of small stock (i.e. sheep, goat and pig) and the average body mass of selected carnivores (see Table S3). The interaction was performed by means of multiplication of the values of small stock density and average value for the selected carnivore body mass (see extended methods Appendix 1 for further details). Areas with color towards red represent high values for the interaction, whereby high densities of small stock co-occur with selected carnivores of high average body mass. These areas are intended as proxies for high potential conflict between small livestock farming and carnivores as opposed to the green areas in the map. Areas beyond the range of any of the 15 vulture species focus of this study are shown in grey.


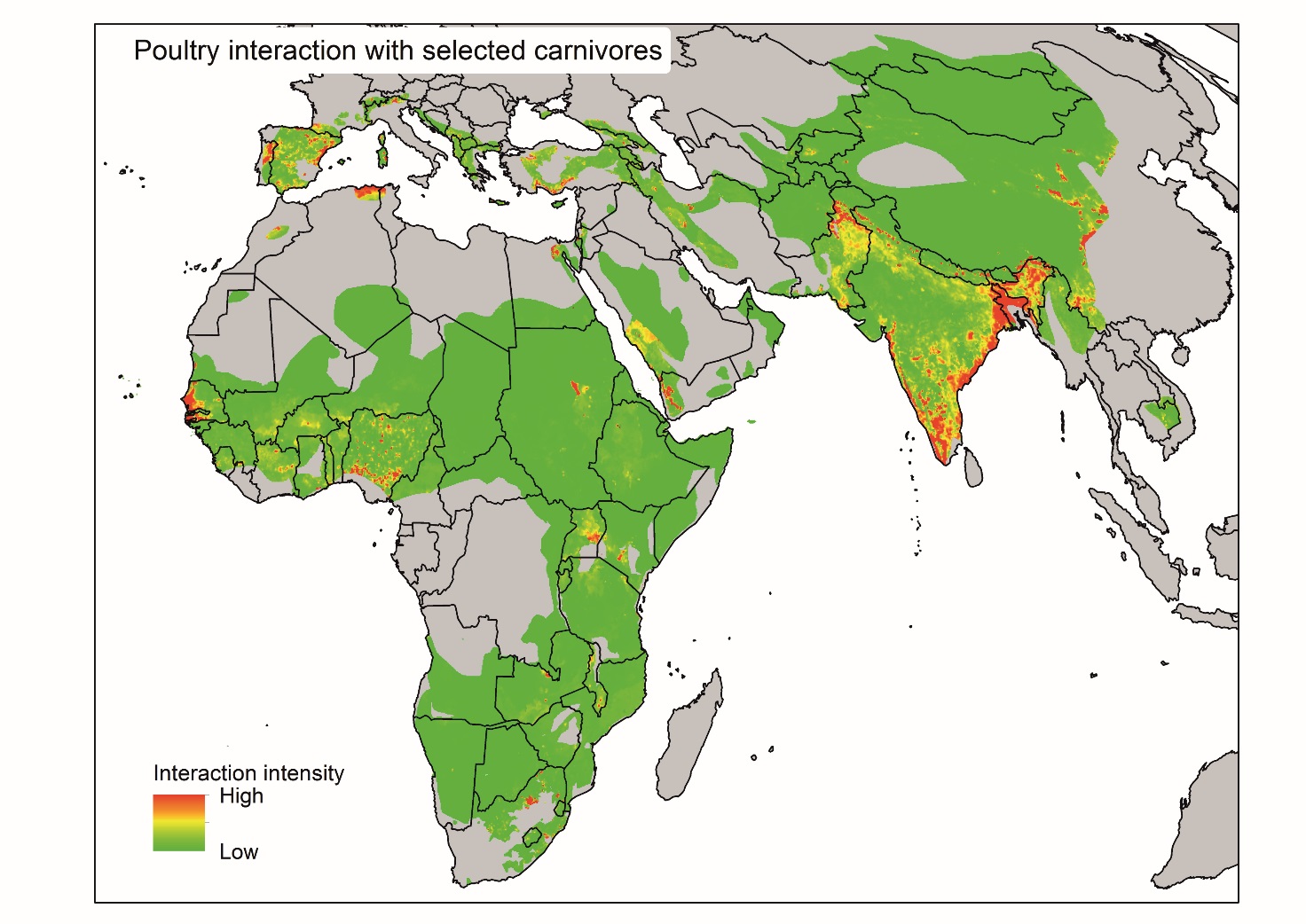


**Figure 4.** Map showing the interaction between density of poultry (i.e. duck and chicken) and the average body mass of selected carnivores (see Table S3). The interaction was performed by means of multiplication of the values of poultry density and average value for the selected carnivore body mass (see extended methods Appendix 1 for further details). Areas with color towards red represent high values for the interaction, whereby high densities of poultry co-occur with selected carnivores of high average body mass. These areas are intended as proxies for high potential conflict between poultry farming and carnivores as opposed to the green areas in the map. Areas beyond the range of any of the 15 vulture species focus of this study are shown in grey.


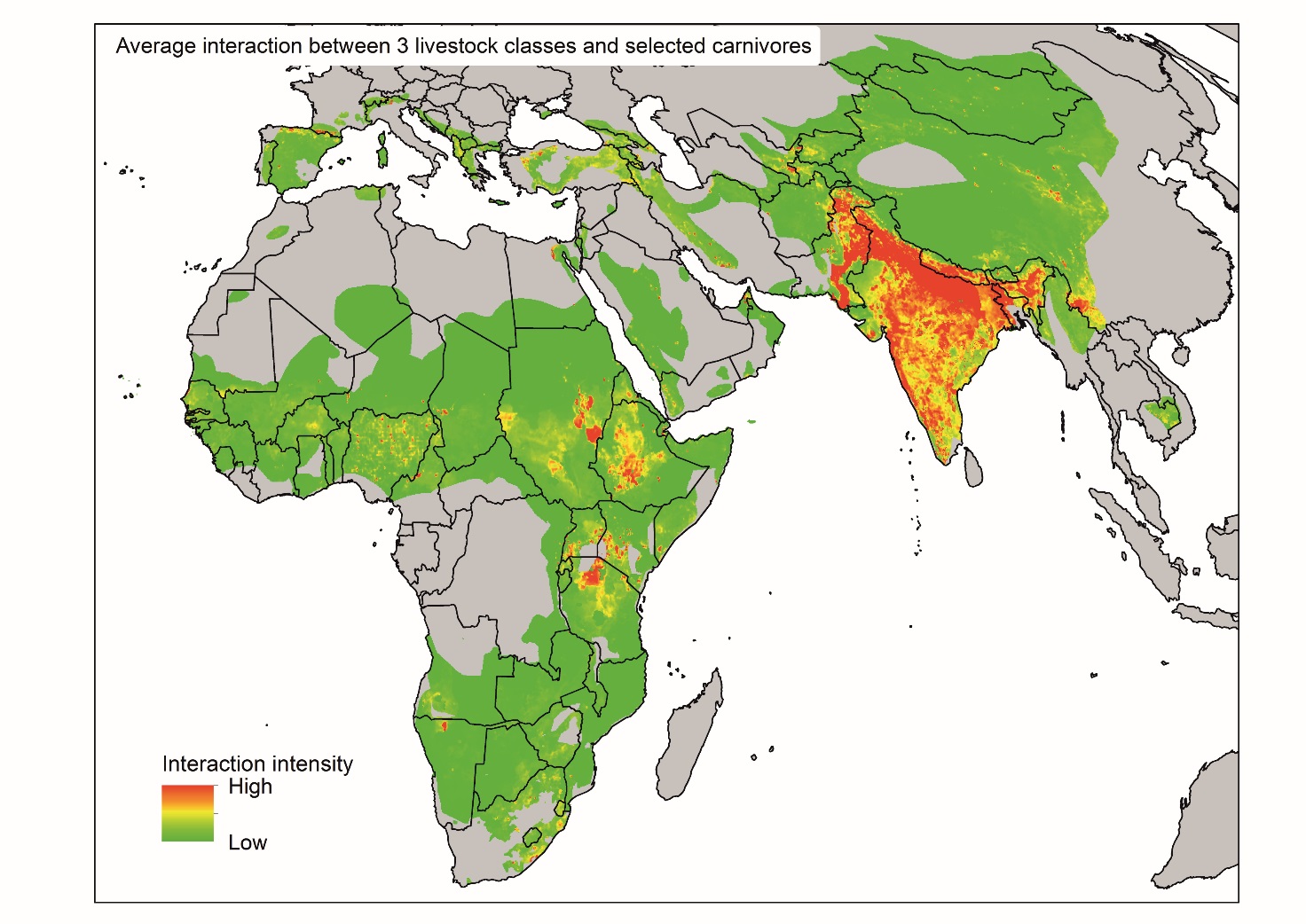


**Figure S5**. The combined map showing the average of the three layers representing the interaction between the three livestock classes (poultry, large stock, small stock) and selected carnivores (Fig. S2 –S4). To derive this map, the weighted average approach was used whereby the values of each of the three input maps (Fig. S2 –S4) were given a weight according to the average body mass (log transformed) of the livestock class in each of the three layers (see extended methods Appendix 1 for further details). Red areas in the map represent high values for the interaction. These areas are intended as having high potential conflict between livestock farming and carnivores as opposed to the green areas. Areas beyond the range of any of the 15 vulture species focus of this study are shown in grey.


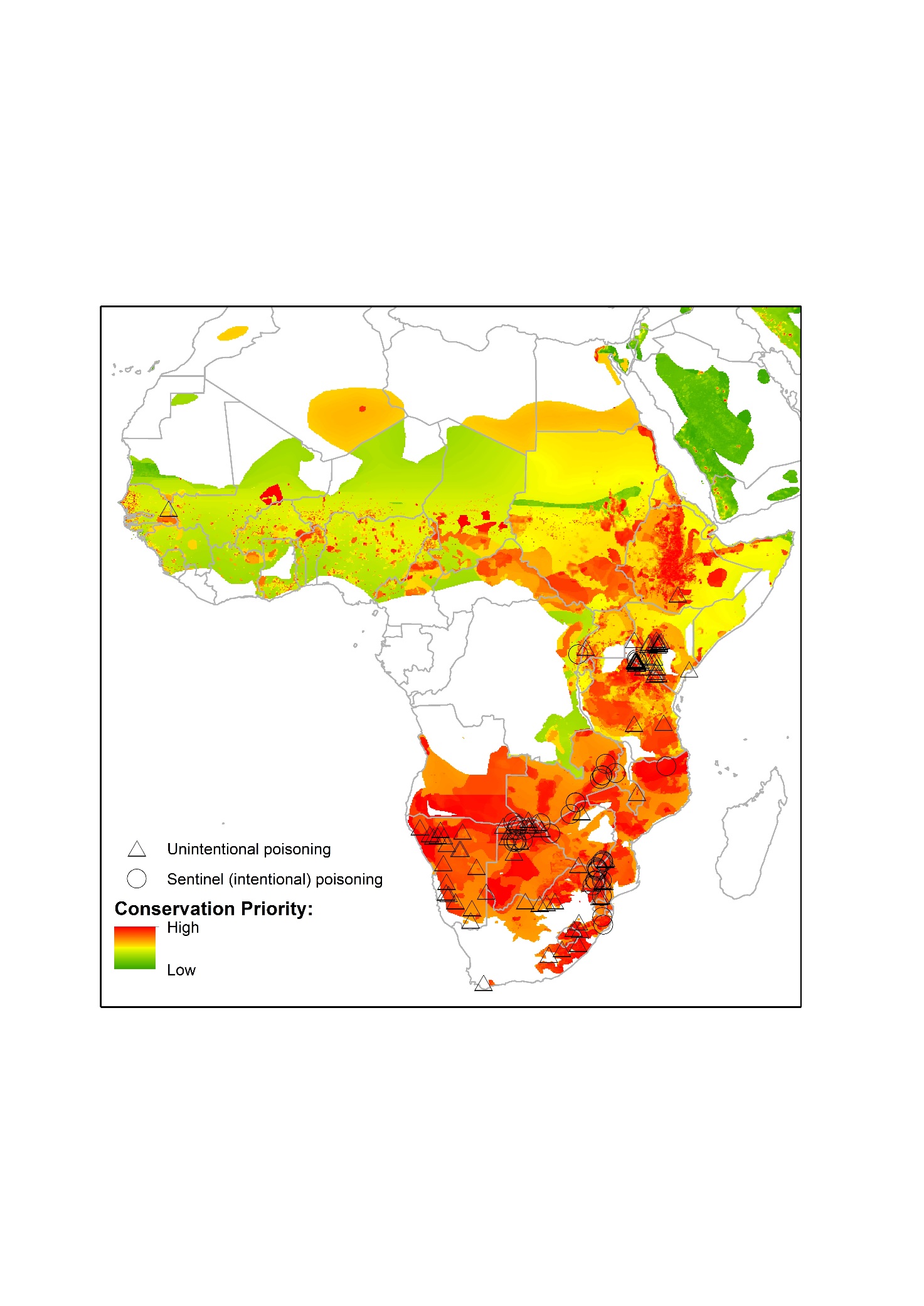


**Figure S6**. The distribution of sentinel (intentional) and unintentional poisoning incidences reported in the African wildlife poisoning database overlaid on the distribution of priority areas for vulture conservation identified through a conservation planning exercise including the distributions of the 15 vulture species and both poisoning threat layers (see Figure S9b below). Note that the database is not complete and nor unbiased, so that poisoning may be well reported in some areas, and less so in other areas. Hence, areas where poisoning incidences are not shown may be due to undetected and/or unreported cases rather than lack of poisoning per se.


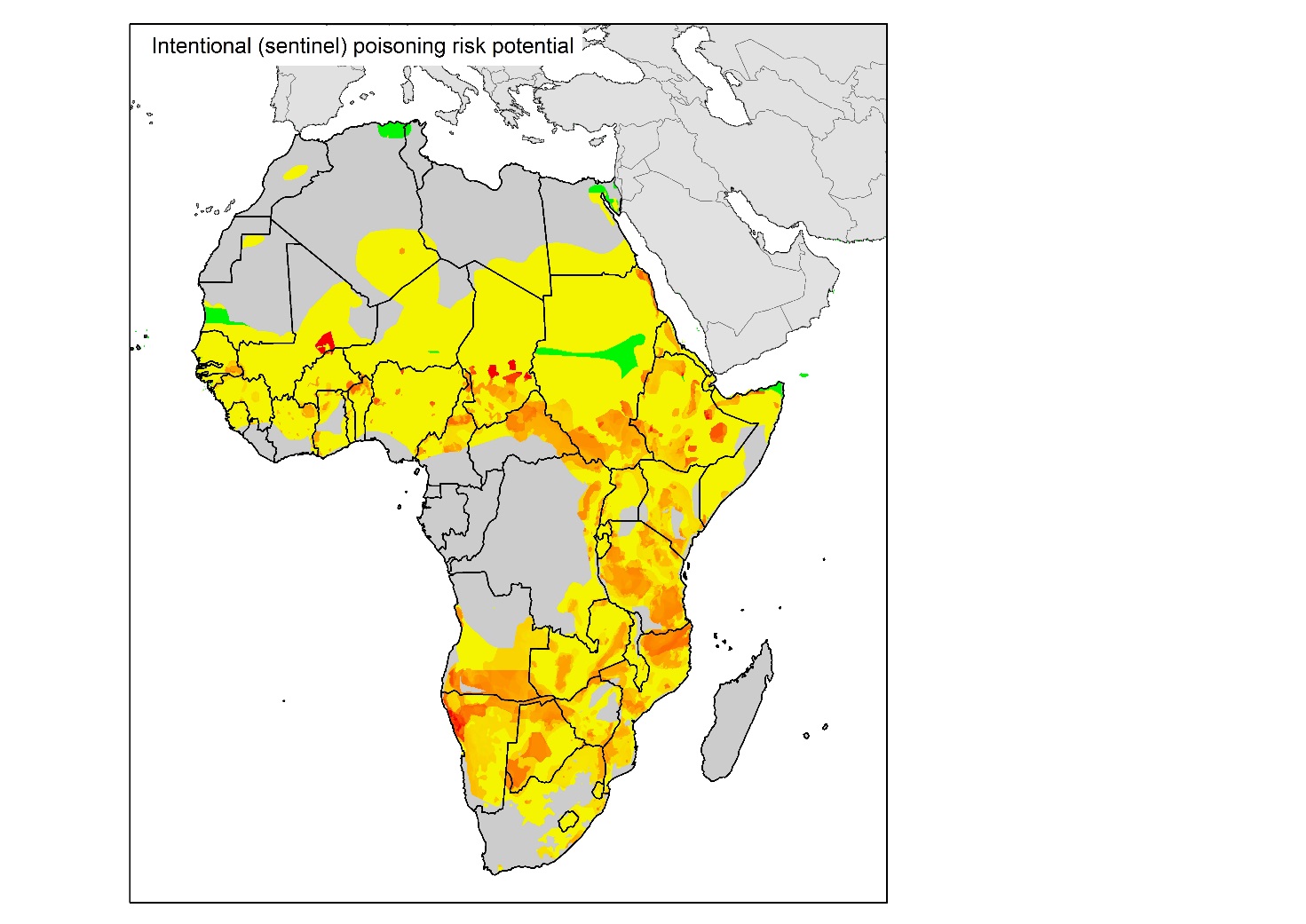


**Figure S7**. Map showing the areas of potentially high risk of intentional (sentinel) poisoning (red colors) across Africa obtained by deriving the per pixel average of an index, the species-specific weight of intentional poisoning (see Table S4), across all species occurring in each 10 km X 10 km pixel in the study region. Areas beyond the study region are shown in grey.


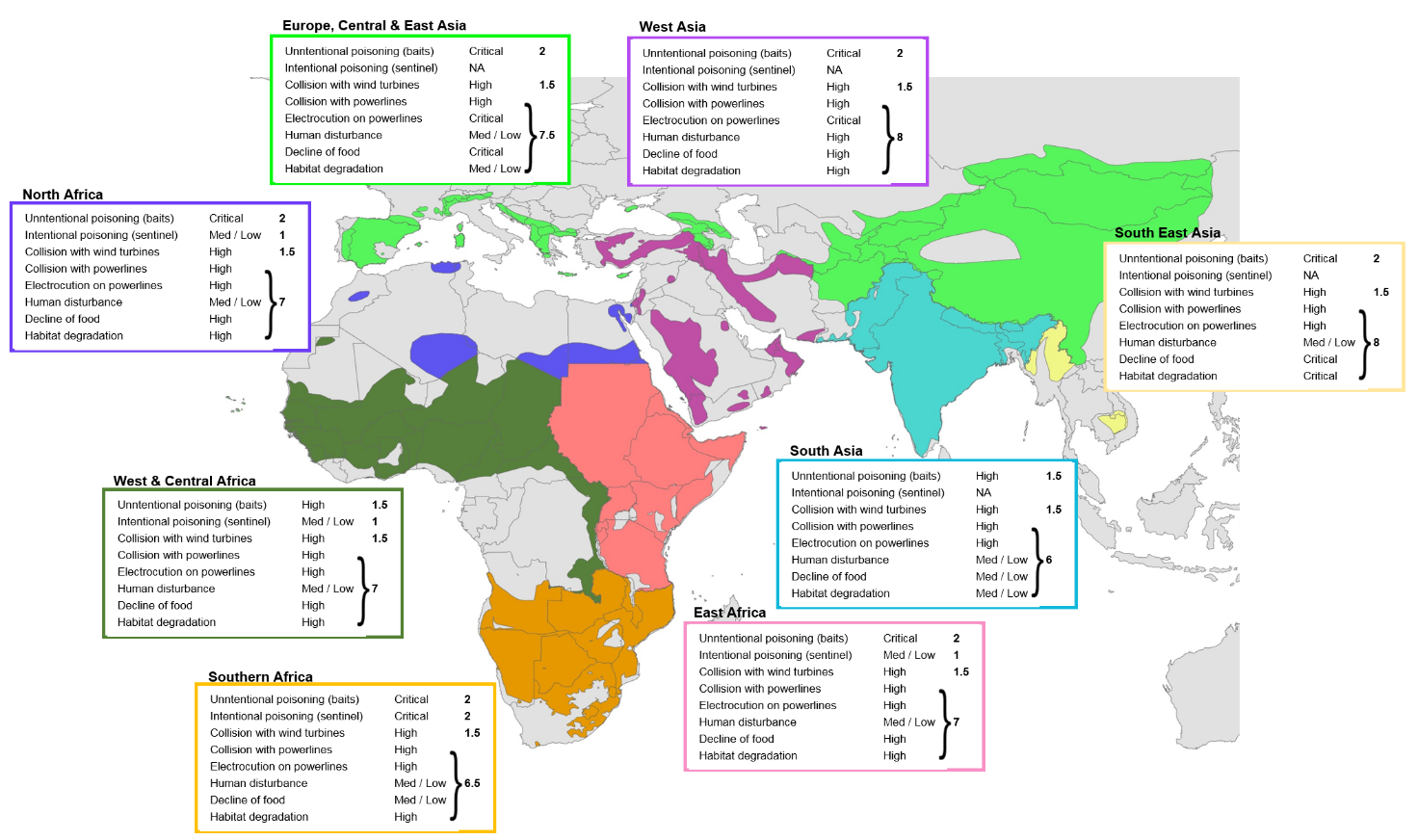


**Figure S8.** The vulture study region across Africa and Eurasia divided in the eight regions (in a different colour) following Botha et al. 2017 (Multi-species Action Plan to Conserve African-Eurasian Vultures). Each threat to vultures was regionally ranked in its priority level by a regional panel of experts. Threat levels used were: Critical, high, medium and low, which are listed in the insets of the map next to the corresponding region (Botha et al. 2017). The present study included spatial layers as proxies for unintentional and intentional poisoning, collision with wind turbines, and a broad layer, namely the Global Human Influence Index, representing a proxy for threats such as collision and electrocution with powerlines, human disturbance, decline in food availability and habitat degradation. Threats for which a spatial layer was not available for the purpose of this study are omitted from the inset lists. Threat levels medium and low in the original document (Botha et al. 2017) were pooled in a single level. The value next to the threat level as assessed by Botha et al. (2017) depicts the weight that we assigned to each threat based on its original priority level (see Table S5). This value was later used to incorporate the expert knowledge information into the spatial layers prior to running the prioritization analyses (see extended methods Appendix 1 for further details).


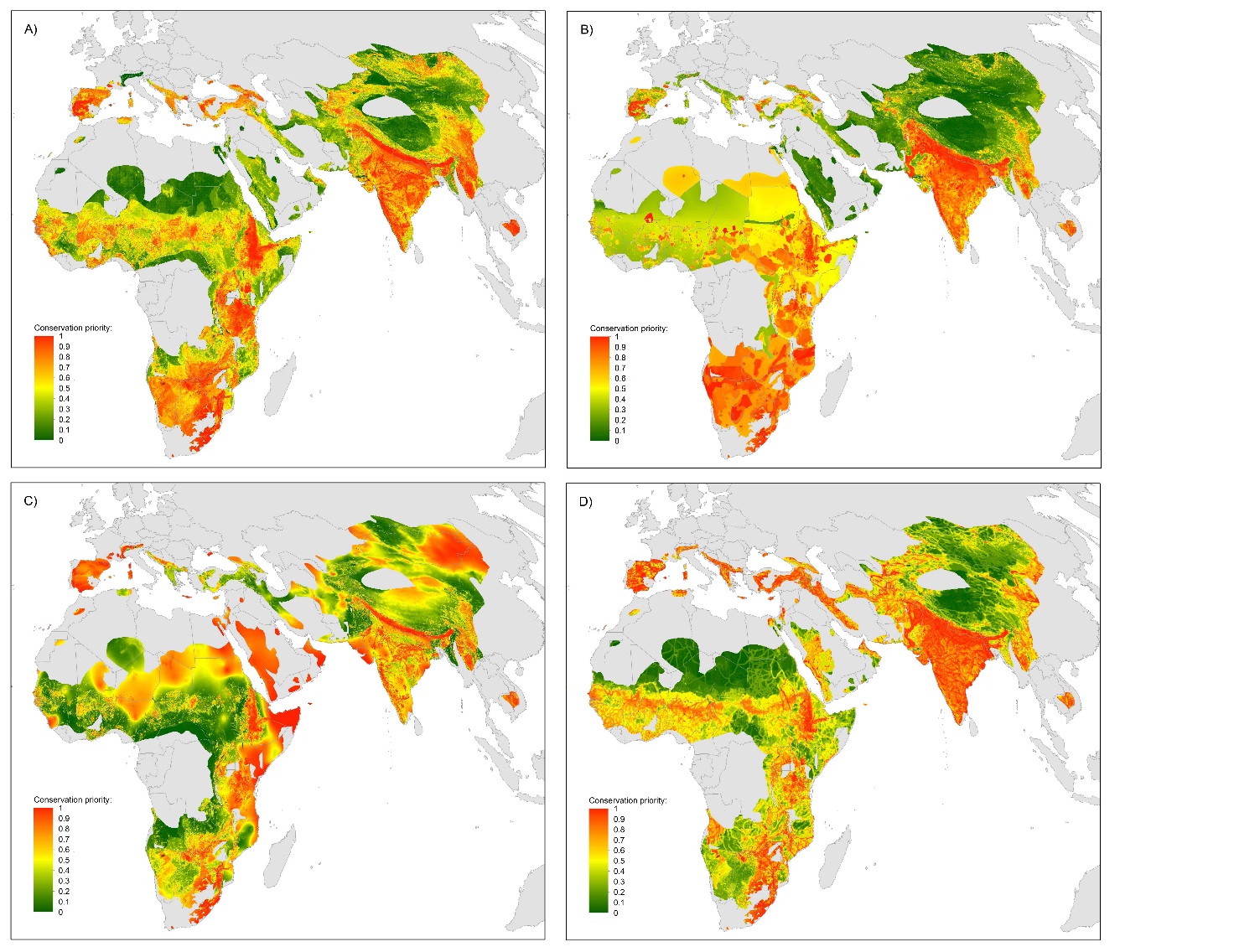


**Figure S9**. The distribution of priority areas for vulture conservation identified through different conservation planning exercises including in turn the distributions of the 15 vulture species only (panel A), the distributions of the 15 vulture species and both poisoning threat layers (B), distributions of the 15 vulture species and wind threat layer (C), distributions of the 15 vulture species and Global Human Influence Index layer (D) across Africa-Eurasia. Grey areas depict places outside of the breeding and resident range of any of the 15 vultures considered. Priorities are ranked from highest (red color) to lowest (green).


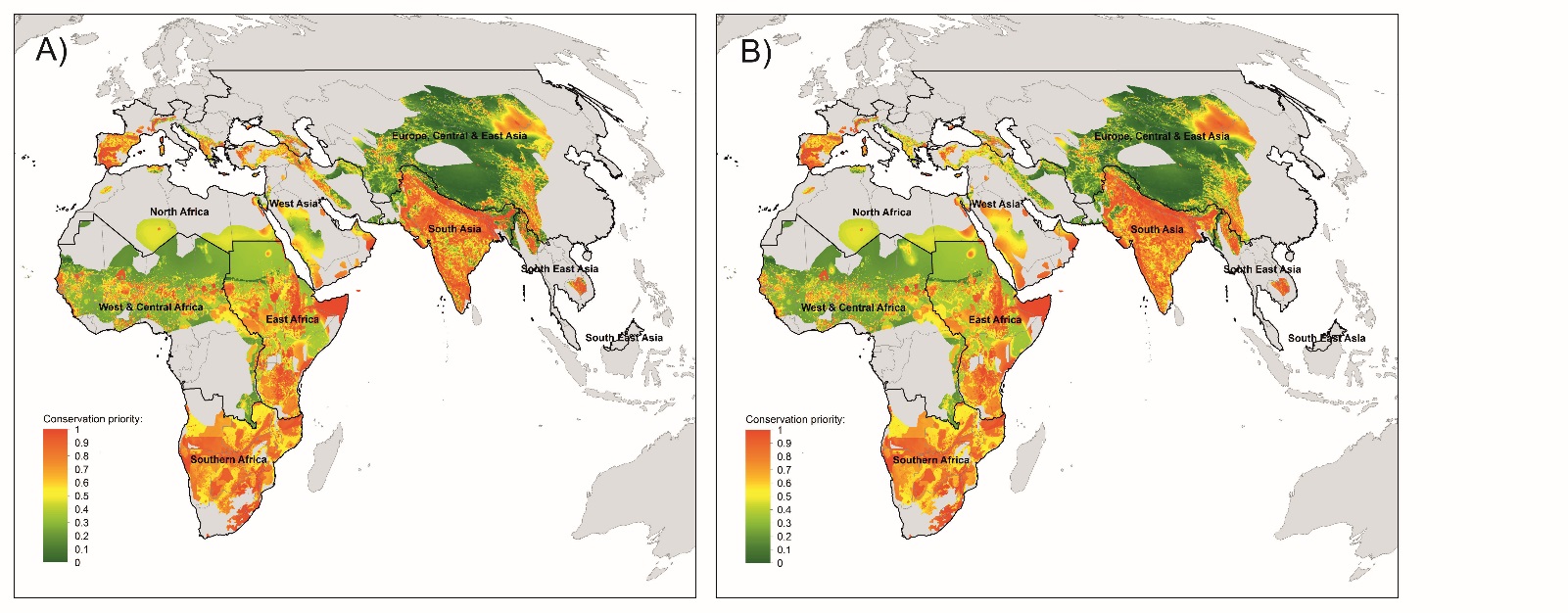


**Figure S10.** The distribution of priority areas for vulture conservation identified as in Figure 1 in the main manuscript but taking in to account (technically penalising) areas of high uncertainty in the species distributions as well as the unintentional poisoning and wind layers (see more details in Supporting Information Appendix 1). Figure A is based on priorities identified when 1 SD (standard deviation) was discounted from the species distributions, unintentional poisoning and wind layers to account for uncertainty, and Figure B when 0.5 SD was discounted. The Pearson correlation between each of the two maps shown here and the map showing the main results (Fig. 1) was r = 0.88 and 0.99 (correlation between Fig. 1 and S10A, and between Fig. 1 and S10B, respectively). This suggests that all these maps are rather concordant in identifying where priorities locate irrespective of the uncertainty associated to the features.


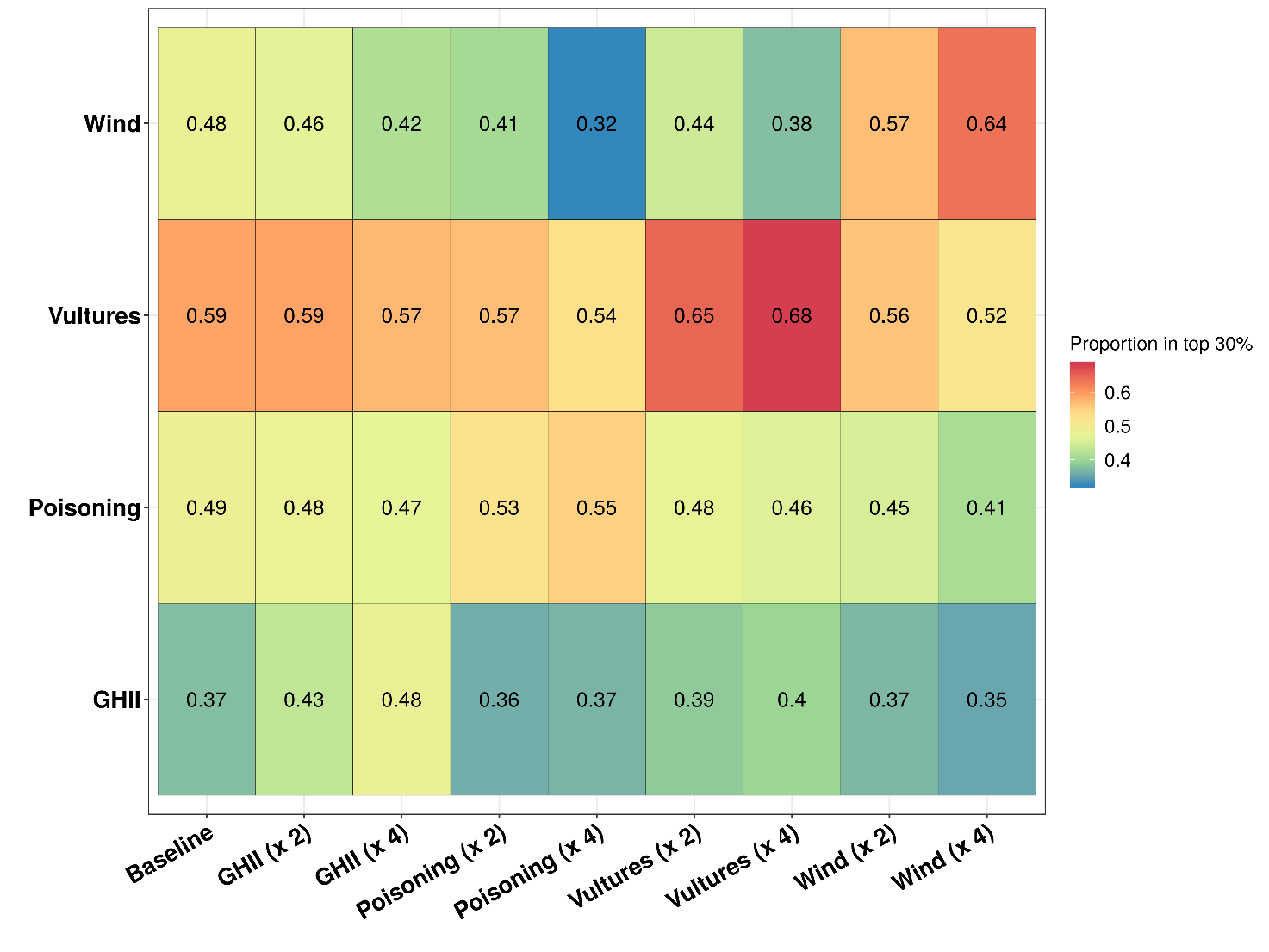
**Figure S11.** The sensitivity of the prioritization results to weight changes assigned to the different features. The proportion of each feature retained in the top 30% fraction of the landscape (values and color gradient within each cell) when the weight of a given feature was varied (see x-axis, the numbers 2 and 4 after the feature name indicate that the weight of that feature was doubled or multiplied by four), while keeping the weights for all the other features constant (see more details in Supporting Information Appendix 1). The baseline represents the situation whereby all features are weighted equally. The red colour indicates that a higher proportion a particular feature was retained in the final landscape. The figure highlights an expected outcome, i.e. when the weight of a given feature is increased by 2 or 4 times, its representation in the top 30% fraction of the landscape consequently increases.
