# Supplementary material for: Priority areas for conservation of Old World vultures
Source: Conserv Biol. 2019 Mar 13;33(5):1056–65. doi: 10.1111/cobi.13282 (PMC6849836; doi:10.1111/cobi.13282)
Supplement: Supplementary file 2 — Supporting Information [file COBI-33-1056-s002.docx]

**Supporting Material Tables:**

**Table S1**. The number of vulture occurrence data used for the species distribution modeling after applying the filters (see Appendix 1 extended methods) on the overall GBIF and ARDB observation data.

| **Species** | **Number of used records** |
| --- | --- |
| Aegypius monachus | 285 |
| Gypaetus barbatus | 443 |
| Gyps africanus | 1327 |
| Gyps bengalensis | 207 |
| Gyps coprotheres | 378 |
| Gyps fulvus | 636 |
| Gyps himalayensis | 213 |
| Gyps indicus | 111 |
| Gyps rueppelli | 383 |
| Gyps tenuirostris | 34 |
| Necrosyrtes monachus | 840 |
| Neophron percnopterus | 982 |
| Sarcogyps calvus | 70 |
| Torgos tracheliotos | 567 |
| Trigonoceps occipitalis | 316 |

**Table S2**. List of the original 35 land-cover categories derived European Space Agency 2012 land-cover map ([www.esa-landcover-cci.org/](http://www.esa-landcover-cci.org/)) and their aggregation in to seven broad and homogenous classes of land-cover used for the species distribution modeling of vulture distributions.

| **Original land-cover categories** | **Pooled land-cover categories** |
| --- | --- |
| Cropland, rainfed | agricultural |
| Herbaceous cover | grassland |
| Tree or shrub cover | woodland |
| Cropland,irrigated or post-flooding | agricultural |
| Mosaic cropland (>50%) / natural vegetation (tree, shrub, herbaceous) | agricultural |
| Tree cover, broadleaved, evergreen, closed to open (>15%) | woodland |
| Tree cover, broadleaved, deciduous, closed to open (>15%) | woodland |
| Tree cover, broadleaved, deciduous, closed (>40%) | woodland |
| Tree cover, broadleaved, deciduous, open (15‐40%) | woodland |
| Tree cover, needleleaved, evergreen, closed to open (>15%) | woodland |
| Tree cover, needleleaved, evergreen, closed (>40% | woodland |
| Tree cover, needleleaved, evergreen, open (15‐40%) | woodland |
| Tree cover, needleleaved, deciduous, closed to open (>15%) | woodland |
| Tree cover, needleleaved, deciduous, closed (>40%) | woodland |
| Tree cover, needleleaved, deciduous, open (15‐40%) | woodland |
| Tree cover, mixed leaf type (broadleaved and needleleaved) | woodland |
| Mosaic tree and shrub (>50%) / herbaceous cover (<50%) | mosaic |
| Mosaic herbaceous cover (>50%) / tree and shrub (<50%) | mosaic |
| Shrubland | shrubland |
| Evergreen shrubland | shrubland |
| Deciduous shrubland | shrubland |
| Grassland | grassland |
| Sparse vegetation (tree, shrub, herbaceous cover) (<15%) | grassland |
| Sparse shrub (<15%) | shrubland |
| Sparse herbaceous cover (<15%) | grassland |
| Urban areas | urban |
| Bare areas | bare |
| Consolidated bare areas | bare |
| Unconsolidated bare areas | bare |

Table S3. The list of 107 carnivore species found within the range of the vulture distributions focus of this study. Species are listed with their latin name along with their body mass in kg obtained from Wilman et al*.* (2014). The third column from the left depicts the matching between each carnivore and the selected livestock prey category/categories based on the outcomes of the literature review. These outcomes are shown in the fourth to sixth columns from the left (poultry, small stock and large stock), whereby any incidence of predation by each carnivore on the selected livestock class is depicted by the value of 1. Empty cells depict lack of information or information not retrieved by our search on predation incidences of the carnivore on the specific livestock class. The rightmost column indicates the code for the source where the incidence was reported, which is listed in full below the table.

| **Latin name** | **Mass (Kg)** | **Livestock prey** | **Poultry** | **Small stock** | | **Large stock** | | **Reference** | |  |
| --- | --- | --- | --- | --- | --- | --- | --- | --- | --- | --- |
| Mustela nivalis | 0,1 | poultry |  | |  | |  | |  | |
| Mustela erminea | 0,12 | poultry |  | |  | |  | |  | |
| Mustela altaica | 0,17 | poultry |  | |  | |  | |  | |
| Mustela kathiah | 0,21 | poultry |  | |  | |  | |  | |
| Poecilogale albinucha | 0,34 | poultry |  | |  | |  | |  | |
| Mustela subpalmata | 0,35 | poultry |  | |  | |  | |  | |
| Mustela sibirica | 0,41 | poultry |  | |  | |  | |  | |
| Mustela lutreola | 0,44 | poultry |  | |  | |  | |  | |
| Vormela peregusna | 0,45 | poultry |  | |  | |  | |  | |
| Poiana leightoni | 0,5 | poultry |  | |  | |  | |  | |
| Poiana richardsonii | 0,5 | poultry |  | |  | |  | |  | |
| Prionodon pardicolor | 0,51 | poultry |  | |  | |  | |  | |
| Herpestes javanicus | 0,75 | poultry | 1 | |  | |  | | 1 | |
| Melogale moschata | 0,81 | poultry |  | |  | |  | |  | |
| Mustela putorius | 0,92 | poultry |  | |  | |  | |  | |
| Genetta servalina | 1,05 | poultry |  | |  | |  | |  | |
| Vulpes zerda | 1,1 | poultry |  | |  | |  | |  | |
| Martes zibellina | 1,13 | poultry |  | |  | |  | |  | |
| Felis nigripes | 1,3 | poultry |  | |  | |  | |  | |
| Ictonyx striatus | 1,3 | poultry |  | |  | |  | |  | |
| Martes martes | 1,3 | poultry |  | |  | |  | |  | |
| Herpestes edwardsii | 1,3 | poultry | 1 | |  | |  | | 1 | |
| Prionailurus rubiginosus | 1,38 | poultry |  | |  | |  | |  | |
| Genetta thierryi | 1,4 | poultry |  | |  | |  | |  | |
| Mustela strigidorsa | 1,5 | poultry |  | |  | |  | |  | |
| Martes foina | 1,54 | poultry |  | |  | |  | |  | |
| Bdeogale crassicauda | 1,55 | poultry |  | |  | |  | |  | |
| Bdeogale omnivora | 1,55 | poultry |  | |  | |  | |  | |
| Paracynictis selousi | 1,64 | poultry |  | |  | |  | |  | |
| Genetta abyssinica | 1,65 | poultry |  | |  | |  | |  | |
| Genetta angolensis | 1,65 | poultry |  | |  | |  | |  | |
| Mustela eversmanii | 1,68 | poultry |  | |  | |  | |  | |
| Melogale personata | 1,7 | poultry |  | |  | |  | |  | |
| Herpestes smithii | 1,78 | poultry |  | |  | |  | |  | |
| Genetta genetta | 1,8 | poultry |  | |  | |  | |  | |
| Genetta bourloni | 1,82 | poultry |  | |  | |  | |  | |
| Genetta pardina | 1,82 | poultry |  | |  | |  | |  | |
| Genetta poensis | 1,82 | poultry |  | |  | |  | |  | |
| Martes flavigula | 1,84 | poultry |  | |  | |  | |  | |
| Herpestes urva | 1,86 | poultry | 1 | |  | |  | | 1 | |
| Genetta cristata | 1,86 | poultry |  | |  | |  | |  | |
| Herpestes fuscus | 1,98 | poultry |  | |  | |  | |  | |
| Nandinia binotata | 2 | poultry AND small stock | 1 | | 1 | |  | | 2 | |
| Martes gwatkinsii | 2,04 | poultry AND small stock |  | |  | |  | |  | |
| Ictonyx libycus | 2,06 | poultry AND small stock |  | |  | |  | |  | |
| Genetta johnstoni | 2,23 | poultry AND small stock |  | |  | |  | |  | |
| Genetta maculata | 2,23 | poultry AND small stock |  | |  | |  | |  | |
| Genetta tigrina | 2,23 | poultry AND small stock |  | |  | |  | |  | |
| Arctogalidia trivirgata | 2,25 | poultry AND small stock |  | |  | |  | |  | |
| Vulpes corsac | 2,4 | poultry AND small stock |  | |  | |  | |  | |
| Bdeogale jacksoni | 2,5 | poultry AND small stock |  | |  | |  | |  | |
| Bdeogale nigripes | 2,5 | poultry AND small stock |  | |  | |  | |  | |
| Felis margarita | 2,52 | poultry AND small stock |  | |  | |  | |  | |
| Herpestes vitticollis | 2,58 | poultry AND small stock |  | |  | |  | |  | |
| Vulpes pallida | 2,8 | poultry AND small stock |  | |  | |  | |  | |
| Pardofelis marmorata | 2,85 | poultry AND small stock |  | |  | |  | |  | |
| Viverricula indica | 2,91 | poultry AND small stock |  | |  | |  | |  | |
| Genetta victoriae | 3 | poultry AND small stock |  | |  | |  | |  | |
| Herpestes naso | 3 | poultry AND small stock |  | |  | |  | |  | |
| Otocolobus manul | 3,05 | poultry AND small stock |  | |  | |  | |  | |
| Vulpes rueppellii | 3,25 | poultry AND small stock |  | |  | |  | |  | |
| Atilax paludinosus | 3,3 | poultry AND small stock |  | |  | |  | |  | |
| Prionailurus bengalensis | 3,3 | poultry AND small stock |  | |  | |  | |  | |
| Nyctereutes procyonoides | 4,04 | poultry AND small stock |  | |  | |  | |  | |
| Otocyon megalotis | 4,15 | poultry AND small stock |  | |  | |  | |  | |
| Paguma larvata | 4,3 | poultry AND small stock |  | |  | |  | |  | |
| Ailurus fulgens | 4,9 | poultry AND small stock |  | |  | |  | |  | |
| Vulpes ferrilata | 5 | poultry AND small stock |  | |  | |  | |  | |
| Felis silvestris | 5,1 | poultry AND small stock |  | |  | |  | |  | |
| Herpestes ichneumon | 5,17 | poultry AND small stock |  | |  | |  | |  | |
| Vulpes vulpes | 5,48 | poultry AND small stock | 1 | | 1 | |  | | 1, 3, 4 | |
| Arctonyx collaris | 6,36 | poultry AND small stock |  | |  | |  | |  | |
| Felis chaus | 7,39 | poultry AND small stock | 1 | |  | |  | | 1 | |
| Mellivora capensis | 8,5 | poultry AND small stock |  | |  | |  | |  | |
| Canis mesomelas | 8,5 | poultry AND small stock |  | | 1 | |  | | 5-8 | |
| Prionailurus viverrinus | 9,14 | poultry AND small stock |  | |  | |  | |  | |
| Viverra megaspila | 9,25 | poultry AND small stock |  | |  | |  | |  | |
| Lynx pardinus | 9,4 | poultry AND small stock | 1 | | 1 | |  | | 9 | |
| Viverra zibetha | 9,5 | poultry AND small stock |  | |  | |  | |  | |
| Arctictis binturong | 9,88 | poultry AND small stock |  | |  | |  | |  | |
| Canis simensis | 10 | poultry AND small stock AND large stock |  | |  | |  | |  | |
| Canis aureus | 10,35 | poultry AND small stock AND large stock |  | | 1 | | 1 | | 7, 10, 11 | |
| Caracal aurata | 10,65 | poultry AND small stock AND large stock |  | |  | |  | |  | |
| Catopuma temminckii | 11,5 | poultry AND small stock AND large stock |  | | 1 | |  | | 7 | |
| Civettictis civetta | 12 | poultry AND small stock AND large stock |  | |  | |  | |  | |
| Leptailurus serval | 12 | poultry AND small stock AND large stock |  | | 1 | |  | | 11 | |
| Meles leucurus | 13 | poultry AND small stock AND large stock |  | |  | |  | |  | |
| Meles meles | 13 | poultry AND small stock AND large stock |  | |  | |  | |  | |
| Caracal caracal | 13,75 | poultry AND small stock AND large stock |  | | 1 | |  | | 7, 8 | |
| Cuon alpinus | 14,17 | poultry AND small stock AND large stock |  | |  | | 1 | | 12 | |
| Gulo gulo | 17,01 | poultry AND small stock AND large stock |  | | 1 | |  | | 3, 7 | |
| Lynx lynx | 17,95 | poultry AND small stock AND large stock |  | | 1 | |  | | 3, 7, 13 | |
| Neofelis nebulosa | 19,68 | poultry AND small stock AND large stock |  | |  | |  | |  | |
| Lycaon pictus | 22,05 | poultry AND small stock AND large stock |  | | 1 | | 1 | | 7, 14 | |
| Canis lupus | 32,18 | poultry AND small stock AND large stock | 1 | | 1 | | 1 | | 7, 8, 12, 13, 15-17 | |
| Hyaena hyaena | 41,71 | poultry AND small stock AND large stock |  | | 1 | |  | | 7 | |
| Parahyaena brunnea | 42,98 | poultry AND small stock AND large stock |  | | 1 | | 1 | | 7 | |
| Panthera uncia | 44,17 | poultry AND small stock AND large stock |  | | 1 | | 1 | | 16, 18 | |
| Helarctos malayanus | 46,5 | poultry AND small stock AND large stock |  | |  | |  | |  | |
| Acinonyx jubatus | 46,7 | poultry AND small stock AND large stock |  | | 1 | | 1 | | 5, 7, 14 | |
| Panthera pardus | 52,04 | poultry AND small stock AND large stock |  | | 1 | | 1 | | 5, 7, 8, 11, 14, 16, 18, 19 | |
| Crocuta crocuta | 66,49 | poultry AND small stock AND large stock |  | | 1 | | 1 | | 5, 7, 8, 11, 14, 19 | |
| Ursus thibetanus | 77,5 | poultry AND small stock AND large stock |  | | 1 | | 1 | | 7, 12, 16, 18, 20 | |
| Ailuropoda melanoleuca | 108,4 | poultry AND small stock AND large stock |  | |  | |  | |  | |
| Panthera leo | 161,5 | poultry AND small stock AND large stock |  | | 1 | | 1 | | 5, 7, 8, 14, 19 | |
| Panthera tigris | 162,56 | poultry AND small stock AND large stock |  | | 1 | | 1 | | 7, 8, 18 | |
| Ursus arctos | 180,52 | poultry AND small stock AND large stock |  | | 1 | | 1 | | 7, 8, 13, 15, 16, 20 | |

**Reference list for table:**

1. Biswas PK*, et al.* (2006) Causes of loss of Sonali chickens on smallholder households in Bangladesh. *Prev. Vet. Med.* 76(3-4):185-195.

2. Campbell M (2009) Proximity in a Ghanaian savanna: Human reactions to the African palm civet Nandinia binotata. *Singapore Journal of Tropical Geography* 30(2):220-231.

3. Warren JT, Mysterud I, & Lynnebakken T (2001) Mortality of lambs in free-ranging domestic sheep (Ovis aries) in northern Norway. *Journal of Zoology* 254(2):195-202.

4. Plumer L, Davison J, & Saarma U (2014) Rapid Urbanization of Red Foxes in Estonia: Distribution, Behaviour, Attacks on Domestic Animals, and Health-Risks Related to Zoonotic Diseases. *PLoS One* 9(12):15.

5. Muriuki MW, Ipara H, & Kiringe JW (2017) The cost of livestock lost to lions and other wildlife species in the Amboseli ecosystem, Kenya. *Eur. J. Wildl. Res.* 63(4):11.

6. Gusset M, Swarner MJ, Mponwane L, Keletile K, & McNutt JW (2009) Human-wildlife conflict in northern Botswana: livestock predation by Endangered African wild dog Lycaon pictus and other carnivores. *Oryx* 43(1):67-72.

7. Breitenmoser U*, et al.* (2005) Non-lethal techniques for reducing depredation. *People and wildlife: conflict or coexistence?*, eds Woodroffe R, Thirgood S, & Rabinowitz A (Cambridge University Press, Cambridge), pp 49 - 71.

8. Miller JRB (2015) Mapping attack hotspots to mitigate human-carnivore conflict: approaches and applications of spatial predation risk modeling. *Biodivers. Conserv.* 24(12):2887-2911.

9. Garrote G*, et al.* (2013) Human-felid conflict as a further handicap to the conservation of the critically endangered Iberian lynx. *Eur. J. Wildl. Res.* 59(2):287-290.

10. Yoram YT, Ashkenazi S, & Viner O (1995) Cattle predation by the golden jackal Canis-aureus in the Golan-Heights, Israel. *Biol. Conserv.* 73(1):19-22.

11. Atickem A, Williams S, Bekele A, & Thirgood S (2010) Livestock predation in the Bale Mountains, Ethiopia. *Afr. J. Ecol.* 48(4):1076-1082.

12. Li XY, Buzzard P, Chen YC, & Jiang XL (2013) Patterns of Livestock Predation by Carnivores: Human-Wildlife Conflict in Northwest Yunnan, China. *Environ. Manage.* 52(6):1334-1340.

13. Karlsson J & Johansson O (2010) Predictability of repeated carnivore attacks on livestock favours reactive use of mitigation measures. *Journal of Applied Ecology* 47(1):166-171.

14. Woodroffe R, Frank LG, Lindsey PA, Ranah S, & Romanach S (2007) Livestock husbandry as a tool for carnivore conservation in Africa's community rangelands: a case-control study. *Biodivers. Conserv.* 16(4):1245-1260.

15. Frank J & Eklund A (2017) Poor construction, not time, takes its toll on subsidised fences designed to deter large carnivores. *PLoS One* 12(4):10.

16. Ahmad S*, et al.* (2016) Carnivores' diversity and conflicts with humans in Musk Deer National Park, Azad Jammu and Kashmir, Pakistan. *Eur. J. Wildl. Res.* 62(5):565-576.

17. Torres RT & Fonseca C (2016) Perspectives on the Iberian wolf in Portugal: population trends and conservation threats. *Biodivers. Conserv.* 25(3):411-425.

18. Sangay T & Vernes K (2008) Human-wildlife conflict in the Kingdom of Bhutan: Patterns of livestock predation by large mammalian carnivores. *Biol. Conserv.* 141(5):1272-1282.

19. Kissui BM (2008) Livestock predation by lions, leopards, spotted hyenas, and their vulnerability to retaliatory killing in the Maasai steppe, Tanzania. *Animal Conservation* 11(5):422-432.

20. Chauhan NPS (2003) Human Casualties and Livestock Depredation by Black and Brown Bears in the Indian Himalaya, 1989-98. *Ursus* 14(1):84-87.

Table S4. The list of 72 species of herbivores and carnivores as identified for being potential target of intentional poisoning by poachers across South Saharan Africa. The weights reported in the relative column represent the relative number of incidences of intentional poisoning involving that species on the log (x+1) scale and as reported in the African wildlife poisoning database. Most species (n = 66) for which no incidences were reported in the poisoning database were given the weight of 0.3, i.e. the weight of the species reported in the database to have been target of intentional poisoning only once. This list of species and associated weights were used to derive the spatial layer of intentional poisoning (Figure S6) across Africa by combining the distribution range of each of the species and their weights (see extended methods Appendix 1 for further details).

| **Latin Name** | **Weight** |
| --- | --- |
| Panthera leo | 1,4 |
| Loxodonta africana | 1,3 |
| Panthera pardus | 0,7 |
| Aepyceros melampus | 0,3 |
| Tragelaphus angasii | 0,3 |
| Hippopotamus amphibius | 0,3 |
| Cephalophus dorsalis | 0,3 |
| Cephalophus ogilbyi | 0,3 |
| Gazella spekei | 0,3 |
| Pelea capreolus | 0,3 |
| Cephalophus niger | 0,3 |
| Eudorcas albonotata | 0,3 |
| Eudorcas thomsonii | 0,3 |
| Gazella dorcas | 0,3 |
| Eudorcas rufifrons | 0,3 |
| Eudorcas tilonura | 0,3 |
| Ammodorcas clarkei | 0,3 |
| Redunca fulvorufula | 0,3 |
| Antidorcas marsupialis | 0,3 |
| Litocranius walleri | 0,3 |
| Nanger soemmerringii | 0,3 |
| Tragelaphus scriptus | 0,3 |
| Redunca redunca | 0,3 |
| Ammotragus lervia | 0,3 |
| Potamochoerus larvatus | 0,3 |
| Nanger granti | 0,3 |
| Cephalophus spadix | 0,3 |
| Redunca arundinum | 0,3 |
| Capra nubiana | 0,3 |
| Addax nasomaculatus | 0,3 |
| Cephalophus jentinki | 0,3 |
| Potamochoerus porcus | 0,3 |
| Phacochoerus aethiopicus | 0,3 |
| Nanger dama | 0,3 |
| Kobus vardonii | 0,3 |
| Cephalophus silvicultor | 0,3 |
| Tragelaphus spekii | 0,3 |
| Kobus kob | 0,3 |
| Beatragus hunteri | 0,3 |
| Tragelaphus imberbis | 0,3 |
| Phacochoerus africanus | 0,3 |
| Kobus megaceros | 0,3 |
| Capra walie | 0,3 |
| Damaliscus pygargus | 0,3 |
| Kobus leche | 0,3 |
| Damaliscus lunatus | 0,3 |
| Hippotragus leucophaeus | 0,3 |
| Oryx gazella | 0,3 |
| Alcelaphus buselaphus | 0,3 |
| Connochaetes gnou | 0,3 |
| Connochaetes taurinus | 0,3 |
| Hylochoerus meinertzhageni | 0,3 |
| Oryx dammah | 0,3 |
| Oryx beisa | 0,3 |
| Kobus ellipsiprymnus | 0,3 |
| Tragelaphus strepsiceros | 0,3 |
| Tragelaphus buxtoni | 0,3 |
| Hippotragus niger | 0,3 |
| Okapia johnstoni | 0,3 |
| Choeropsis liberiensis | 0,3 |
| Equus africanus | 0,3 |
| Hippotragus equinus | 0,3 |
| Equus zebra | 0,3 |
| Tragelaphus eurycerus | 0,3 |
| Equus quagga | 0,3 |
| Equus grevyi | 0,3 |
| Tragelaphus oryx | 0,3 |
| Syncerus caffer | 0,3 |
| Tragelaphus derbianus | 0,3 |
| Giraffa camelopardalis | 0,3 |
| Diceros bicornis | 0,3 |
| Ceratotherium simum | 0,3 |

**Table S5**. The relative weight assigned to each of the four main threats (or proxy for multiple threats) to vultures across the eight regions in Africa and Eurasia where these threats have been assessed and ranked by panels of local and international experts as part of the development of the Multi Species Action Plan to conserve African-Eurasian vultures (Botha et al*.* 2017). The values for these threats have been used to refine the spatial layer of each of the four original threats used in this study by simply multiplying the pixel value of each threat by its relative region-specific weight as reported in this Table (see also Fig. S7). GHII depicts the Global Human Influence Index, which is here used as a combined proxy for multiple threats including collision with, and electrocution on, energy infrastructures (mainly powerlines), human disturbance, habitat degradation (including the loss of nesting trees) and decline in food availability. Intentional poisoning was given a value of zero beyond Africa where this threat has never been reported before.

| **Region:** | **Unintentional poisoning** | **Intentional (sentinel) poisoning** | **Wind energy** | **GHII** |
| --- | --- | --- | --- | --- |
| Southern Africa | 2 | 2 | 1,5 | 6,5 |
| West-Central Africa | 1,5 | 1 | 1,5 | 7 |
| East Africa | 2 | 1 | 1,5 | 7 |
| North Africa | 2 | 1 | 1,5 | 7 |
| Europe Central-East Asia | 2 | 0 | 1,5 | 7,5 |
| West Asia | 2 | 0 | 1,5 | 8 |
| South-East Asia | 2 | 0 | 1,5 | 8 |
| South Asia | 1,5 | 0 | 1,5 | 6 |

**Table S6**. List of explanatory variables considered for the model on the national share of the top 30% priority areas for vulture conservation in Africa and Eurasia. The description of each variable, along with the unit, year and source of the information are also given.

| **Factors name:** | **Description:** | **Unit:** | **Year:** | **Source:** |
| --- | --- | --- | --- | --- |
| HDI | Composite index of health (life expectancy),education (years of schooling) and wealth (gross national income per capita) | Index from 0 = least developed, to 1 = best developed | 2015 | World Bank |
| Governance | The average of six indicators that measure governance and that are all scaled in the same way: voice and accountability, political stability and absence of violence, government effectiveness, regulatory quality, rule of law, control of corruption | Index from -2.5 = poorest governance,to 2.5 = best governance | 2016 | World Bank |
| Country size | The land area of the country | Km2 | 2017 | Global Administrative Areas Database |
| % Terrestrial PAs | The fraction of land in each country that is covered by Protected Areas (PA) | percentage over the total land area | 2014 | World Bank |
| Rabies Incidence | Per capita death rate from rabies over 100,000 people | Death rate / 100,000 | 2015 | Hampson et al. 2015 |
| Rabies Costs | Total costs related to the burden of rabies impacts on the national public health sector budgets, local communities and livestock economies in USD in 2010 | USD | 2015 | Hampson et al. 2015 |

Data from the World Bank was retrieved from [www.govindicators.org](http://www.govindicators.org), whereas data from Global Administrative Areas Database was obtained from [www.gadm.org](http://www.gadm.org), and the rabies data from Hampson, K., Coudeville, L., Lembo, T., Sambo, M., Kieffer, A., Attlan, M., Barrat, J., Blanton, J.D., Briggs, D.J., Cleaveland, S., et al. (2015). Estimating the Global Burden of Endemic Canine Rabies. PLOS Neglected Tropical Diseases *9*, e0003709.
