# Supplementary material for: Priority areas for conservation of Old World vultures
Source: Conserv Biol. 2019 Mar 13;33(5):1056–65. doi: 10.1111/cobi.13282 (PMC6849836; doi:10.1111/cobi.13282)

**Priority areas for Old World vulture conservation**

<https://doi.org/10.1111/cobi.13282>

**Supporting Information**

**Appendix S1: Supplementary text detailing the production of the vulture distributions and the spatially explicit threat layers, and on the sensitivity analyses**

*Vulture occurrence and environmental data*

Vulture occurrence data were derived primarily from the Global Biodiversity Information Facility (GBIF) database, which was complemented with vulture observations for Africa obtained from the African Raptor DataBank (ARDB; see Appendix S2). Data from GBIF were downloaded for the 15 species (see citations with GBIF download links for each of the species downloads in the reference list below) by supplying the species binomial name to a modified version of the occ_download function from the rgbif R package (Chamberlain 2017). All the occurrence records available from GBIF were downloaded. The data were subsequently filtered and we retained only records from 1980 onwards. While local extinction processes may have occurred during this timeframe, our aim was to capture the range of environmental conditions experienced by the species. Furthermore, most records (over 70%) for the 15 species considered here were collected from 1990 onwards. For the purpose of building species distribution models (see below) we cleaned the dataset of all observations according to two criteria. First, we eliminated any duplicate record by screening for unique combinations of species name and date of collection. Second, we filtered all remaining records in order to retain only those with minimum distance of 30km between nearest observations, using the spatial thinning algorithm developed by Aiello-Lammens et al. (2015). This procedure reduces the potential effects of spatial autocorrelation and sampling bias. A summary, with the number of records used for each species, is provided in the supplementary material Appendix S2.

Next, we derived a set of environmental predictors of vulture occurrence, including land-cover, climate and topography, from different sources. Climatic data at the 5 arc minute resolution were obtained from the worldclim database (Fick & Hijmans 2017). We considered two climatic variables: mean annual temperature and total annual precipitation. These variables are known to represent physiological requirements of species (Barbet-Massin et al. 2012). We decided not to use any seasonal temperature or precipitation variables as these are strongly correlated with the aforementioned annual temperature and precipitation. Two topographic variables, slope and aspect, were derived from a Digital Elevation Model available at a 90m resolution (Jarvis et al. 2008). The aspect variable was derived following the procedure described in Hofierka et al. (2009) and expressed in degrees indicating the direction that slopes are facing. Seven land-cover variables were derived from a European Space Agency 2012 land-cover map (www.esa-landcover-cci.org/), available at a 300m resolution. The dataset contains 35 land-cover categories that were aggregated into seven categories (see supplementary material Appendix S3), including: agricultural, bare, grassland, mosaic vegetation, shrubland, urban and woodland. All the environmental data were up-scaled to a resolution of 10km x 10km. Climatic and topographic variables were up-scaled by taking the mean of the pixels contained within each of the squares of a 10 km x10 km grid. The land cover dataset was up-scaled by calculating the proportion of each land cover type contained within each square of the same grid.

*Species distribution modelling*

An ensemble of species distribution models, which is deemed superior than relying on a single method (Urban et al. 2016), was generated separately for each of the 15 vulture species considered. Methods used included: 1) Generalized Linear Models (GLM; McCullagh & Nelder 1989) with linear terms combined with a stepwise procedure using Akaike Information Criterion (AIC) to select the best model. 2) Random Forest (RF; Breiman 2001). The output of a RF model depends on two parameters: the total number of trees and the number of variables used at each split (mtry). In order to optimize the RF models we performed a grid search and varied the number of trees 500 to 2000 trees varied the mtry parameter from 2 to 4. The best performing models were those with the default number of trees (500) and number of variables randomly sampled as candidates at each split (mtry=$\sqrt{x}$, where x is the number of predictors). 3) Boosted Regression Trees (BRT; Friedman 2001) using a 10-fold cross-validation procedure to select the optimal number of boosted regression trees. The trees were gradually added to the model in groups of 100 and a small learning rate (0.001) that represents contribution of each tree to the growing model (Elith et al. 2008). 4) Maxent. The complexity of Maxent models can be adjusted through the regularization multiplier (β-multiplier) and feature classes. To prevent overfitting we chose linear, quadratic and product features. We tested 11 different β-multiplier values (from 0 to 5 in steps of 0.5). The optimal β-multiplier was chosen through a 10-fold cross-validation procedure (Muscarella et al. 2014).

For each technique, presences and pseudo-absences used to calibrate the model were weighted (biomod2) to ensure neutral (0.5) prevalence. The performance of the models was assessed by randomly splitting ten times the data, keeping 70% of them to generate the models and employing the remaining 30% to evaluate their performance based on the AUC criterion and true skill statistic (TSS) statistic (see Appendix S4). After elimination of all models with an AUC < 0.7 or TSS < 0.4, we generated for each species a consensus model (i.e. the ensemble), in which the contribution of each individual technique was proportional to its AUC. The consensus models were used to produce habitat suitability maps for all the 15 vulture species. In order to avoid possible issues related to model overprediction, the suitability maps for the 15 vulture species were further refined using the range map dataset available from Birdlife International and last updated in autumn 2015(BirdLife International and NatureServe, 2015) This dataset contains the vector data on the breeding and resident distribution of the World’s bird species (BirdLife International and NatureServe, 2015). The final outcome is a cropped habitat suitability map for each of the 15 vulture species at the resolution of 10km x 10km within the range of the species, with values ranging from zero, i.e. highly unsuitable area, to one, most suitable area. While refining the distribution of a species may provide a conservative estimate of the distribution of the 15 vulture species, the distributions of African and Eurasian vultures are well known and omission errors are likely to be minimal (Botha et al. 2017; Simmons et al. 2015). All the above analyses were performed using R v 3.4.1 (R Core Development Team 2016) integrated within bash script in a Unix environment.

*Unintentional poisoning*

Unintentional vulture poisoning typically occurs in areas where farmers use poison laced carcasses and baits to kill carnivores as a retaliatory measure resulting from livestock depredation (Buechley & Şekercioğlu 2016; Mateo-Tomas et al. 2012; Ogada et al. 2016b). As such, human-carnivore conflict is a strong determinant of unintentional poisoning risk (Mateo-Tomas et al. 2012; Santangeli et al. 2016a). Therefore, we derived a map showing the intensity of human-carnivore conflict across the study region by interacting carnivores and livestock distributions matched by their body size (so as to avoid unrealistic interactions between, e.g. very small carnivores and large stock; see below for more details). In doing so, we used two sets of information, the distribution of density of livestock (Robinson et al. 2014) and the distribution range of selected carnivore species (obtained from IUCN; www.iucn.org). Global distribution maps of livestock (duck, chicken, goat, sheep, pig, cattle, buffalo) densities at the resolution of 1km x 1km were obtained from Robinson et al. (2014). For the purpose of this study, we classified livestock into three homogeneous size classes, hereafter named poultry (including summed density of duck and chicken), small stock (summed density of sheep, goat and pig) and large stock (cattle and buffalo densities summed).

We then selected all terrestrial carnivores (order Carnivora) of Africa and Eurasia for which the range at least partly overlapped with the study region considered for this study. From the resulting list, we only selected those species for which the diet is comprised at least 10% of endotherm vertebrate prey (diet trait data retrieved from Wilman et al. (2014). This resulted in 107 carnivore species that are potential candidates of triggering a human-carnivore conflict in the study region. We then conducted a literature search to identify, for each of the 107 carnivore species, the type of livestock (considering the three broad categories as above: poultry, small and large stock) they have been reported to attack. This literature search was conducted in Web of Science in October 2017 using the combination of keywords such as “attack” AND “the latin name of each carnivore” OR “the common names of each carnivore” (e.g. “attack” AND “panthera leo” OR “lion” OR “African lion”). The keywords using latin and common names of the species were based on names derived from IUCN for each species. The identified documents were then screened first through the title, and then the abstract and ultimately the full text to identify those documents that focused on human-carnivore conflict involving the focal carnivore species. From this filtered selection of documents, we extracted information on which carnivore predated on which livestock type. We only extracted information where both carnivore and livestock were reported and identified at the species level (see Appendix S5). This literature search was not intended to be fully exhaustive but to give an indication of the type of livestock that each predator has been reported to attack. The results of the literature search indicated that small stock (sheep, goat or pig) could only be predated by carnivores weighting from 2kg and above, and that large stock (cattle or buffalo, including calves) could only be predated by carnivores weighting at least 10kg (see Appendix S5). This information was then used to match the carnivore species with the livestock type based on their respective size. We worked under the assumption that poultry can be predated by carnivores of any size (n = 107 carnivore species). Conversely, based on the above literature search results, small stock could only be predated by any carnivore of size 2kg and above (n = 65), and large stock could only be predated by any carnivore of 10kg of body mass and above (n = 27). We then used the three groups of carnivores (i.e. all carnivores, carnivores with body mass ≥ 2kg, and carnivores with body mass ≥ 10kg), combined with the carnivore ranges, to derive three maps, each showing the average body mass of the selected carnivores occurring in each 10km x 10km grid cell. We used the average body mass of selected carnivores because we were interested in a community-weighted mean trait value of the carnivores composing a specific assembly. Community-weighted mean trait values have been shown to associate with ecosystem processes, among others (see e.g. Díaz et al. 2007). Next we interacted (by means of multiplication, e.g. for each grid cell, the value from one map was multiplied by the value of the other map) each of the three livestock maps (e.g. map of summed density of poultry, small and large stock) with the respective carnivore average body mass map (see above). That is, the poultry density map was multiplied with the map of average body mass of all carnivores, the small stock density map was multiplied with the map of average body mass of carnivores of size 2kg and above, and the large stock density map was multiplied with the map of average body mass of all carnivores of size 10kg and above. This resulted in three separate maps (presented in Appendix S6-8), each showing the potential for interaction of each livestock type and the selected carnivores. We then combined these three maps (interaction between poultry, small and large stock with selected carnivores) into a single layer by calculating a weighted average among the three. Specifically, for each grid cell we averaged across the values of the three maps weighted by the body mass of the focal livestock from each of the three maps. The weighted average was preferred to a simple average among the three layers because it allows to give different importance to potential predation on livestock of different size. This is important to consider because the economic loss from predation on the three livestock types is typically very different (e.g. a cow has a much higher economic value than a goat), with this difference most plausibly associated with different levels of retaliatory predator killing e.g. using poison. We derived the average body mass of each livestock type, e.g. from Jones et al. (2009), resulting in an approximate value of 720 kg body mass for large stock, 120 kg for small stock and 2,4 kg for poultry. Using these crude body mass averages as weights for combining the three livestock-carnivore interaction maps would have resulted in a map of potential human-carnivore conflict largely dominated by large stock interaction with large carnivores, whereas in many areas human-carnivore conflict, and resulting poison use, is triggered by predation on small stock (see Miller 2015; Santangeli et al. 2016a; Woodroffe et al. 2005). Thus we log transformed the average body mass of the three livestock types to reduce the relative difference between them, and used this transformed value for deriving the combined weighted average map showing the potential for interaction between all livestock and all carnivores combined (map shown in Appendix S9). This layer is used here as a proxy for unintentional poisoning resulting from the human-carnivore conflict. We rescaled the values of the final map to range between zero (least potential for unintentional poisoning) to one (highest potential for unintentional poisoning). We also compared the above with a map derived by simply averaging the three above mentioned layers without using any weights. The output map (not shown here) is very similar to that used here and produced with the weighted average.

Finally, we used an independent source of data from a database recording most of the reported poisoning incidences across Africa and the reason for poisoning (African wildlife poisoning database www.africanwildlifepoisoning.org). While this database is nor fully comprehensive of all incidences of poisoning, it can still be useful for visually investigating how the resulting priority areas for vulture conservation (based on the 15 vulture species and poisoning layers) match with the above described poisoning locations. From the poisoning database, we thus selected only those poisoning incidences for which the verified reason was conflict, i.e. referring to unintentional poisoning (n = 83 cases falling within the range of our mapped study region) or intentional (sentinel) poisoning (n = 37 cases). This visual validation, albeit restricted to Africa (see Appendix S10), suggests that poisoning events mostly occur in those areas we identified as high priority for vulture conservation (based on the 15 vulture distributions and the poisoning threat; Appendix S16).

*Intentional (sentinel) poisoning*

Intentional poisoning is typically done by poachers aiming to kill vultures that circle around poached animals and alert authorities of the location of the illegal activity across Sub-Saharan Africa (Ogada et al. 2016a). Particularly in Southern Africa intentional poisoning has been identified as a critical threat to vultures (Botha et al. 2017). In order to derive a spatial layer for this threat, we first identified the herbivore or carnivore species targeted by poachers and that are poisoned to kill vultures. These target species are typically of large size and have high value as bushmeat or for their body parts (e.g. lion *Panthera leo*, leopard *Panthera pardus*, elephant *Loxodonta africana*). We used a recently published database on reported poisoning incidences across Africa (African Wildlife Poisoning Database www.africanwildlifepoisoning.org) to identify the species target of intentional poisoning and the relative frequency in which they are targeted. In doing so, we filtered all poisoning incidences where the known reason was poaching and which dated from 2007 onwards. The latter criterion was used because sentinel poisoning is a relatively recent phenomenon (Ogada et al. 2016a). The above filtering yielded a total of 51 incidences of sentinel poisoning, of which the majority involved a few species, such as lion (n = 25), elephant (n = 19), leopard (n = 4), and with one incidence involving Hippo *Hippopotamus amphibius*, Impala *Aepyceros melanpus* and Nyala *Tragelaphus angasii*. The above search results suggest that medium to large sized herbivores (e.g. body mass of 53kg of the impala, and above) and selected carnivores are target of intentional poisoning, with some species being more targeted than others. As the database on poisoning incidences is not comprehensive of all incidences, we used that information as a broad reference and conservatively selected all Cetartiodactyla and Perissodactyla species of Africa with body mass ≥ 20 kg (the weight of a large duiker or a small gazelle), as well as other three selected species, such as Elephant, Lion and Leopard, for deriving a map of intentional poisoning across Africa, resulting in 72 species overall (see Appendix S11). While the above threshold of 20 kg is somewhat arbitrary, it represents a good balance between including all species large enough to be potential target of intentional poisoning, but exclude those that are too small to be poisoned to kill vultures. Note that when small herbivores are poached, typically for bushmeat, the whole carcass can be taken away by poachers, with nothing left on the scene to attract vultures, and consequently no need to use poisons. Furthermore, because a few species, namely elephant, lion and leopard, had much higher reported incidences in the poisoning database compared to the other ungulate species, we incorporated this information for mapping intentional poisoning by assigning a weight to each species based on the number of reported incidences for that species. As this number varied greatly among the species, and given that the database is not fully comprehensive of all poisoning incidences, we conservatively applied a log + 1 transformation to those species-specific incidence numbers to reduce the difference in weight between the species. This transformation was deemed appropriate, as it allowed to retain the difference in poisoning incidence among the target species, e.g. elephant having 10 times the weight of all other ungulates (see Appendix S11), but at the same time avoid the output layer be largely driven by the occurrence of elephants, which would be unrealistic. All those species from the list of 72 for which no poisoning incidence was reported from the African wildlife poisoning database were assigned the weight of the species reported only once in the database (that is, they were given a weight = 0.3 or the log(1+1)).

We then used the IUCN range maps of the 72 species target of intentional poisoning in combination with the species-specific weight to derive a single map of sentinel poisoning risk. In doing so, we calculated the average of the species-specific weight of intentional poisoning across all species present in each 10km x 10km pixel within the range of the study area in Africa (see Appendix S12).

*Wind collision risk*

As a proxy for exposure to collision with wind energy infrastructures, we used a map of wind power potential calculated based on the incident wind harnessed by a horizontal axis wind rotor obtained from Pogson et al. (2013); see this publication for further details on how this map of wind power potential was derived). This map of potential unrestricted wind energy was shown to represent a good proxy for installed national wind energy capacity (see Santangeli et al. 2018) and also highlights areas where future development of wind energy infrastructure will most likely take place due to the availability of this renewable resource and the unprecedented positive trend of wind energy deployment worldwide (REN21 2017). The same wind power potential layer used here has also been successfully used in recent studies addressing the impact of renewable energy expansion on biodiversity conservation (Santangeli et al. 2016b; Santangeli et al. 2016c). In this study, we use wind energy potential without any restrictions imposed by cost, accessibility or energy demand (*sensu* Santangeli et al. 2016c). This decision is based on a conservative approach whereby all areas with moderate to high wind energy potential in the region are likely to be developed for wind energy production, thereby representing a current and incoming threat to vultures (Botha et al. 2017; Ogada et al. 2016b; Pearce-Higgins & Green 2014). Applying restrictions based on energy production costs, distribution and demand would likely underestimate the threat posed by potential wind energy infrastructure development in vast areas of low accessibility in the study region. Development in these remote regions may however carry the additional threat from electrocution and collision with overhead transmission lines as the electricity grid network is expanded (Ogada et al. 2016b; Pearce-Higgins & Green 2014). This further underscores the need to take a conservative approach when relating wind energy potential distribution to wildlife.

*Human influence index*

While poisoning and collision with wind energy infrastructures are among the most critical threats to vultures in Eurasia and Africa (Buechley & Şekercioğlu 2016; Ogada et al. 2016b), the recent Multi Species Action Plan to conserve African-Eurasian vultures (Botha et al. 2017) also listed, among others, five additional high to moderate threats to vultures. These are represented by collision with, and electrocution on, energy infrastructures (mainly powerlines), human disturbance, habitat degradation (including the loss of nesting trees) and decline in food availability. All these five threats are somewhat related to the development of areas for infrastructures and other land-uses aimed at producing food or fibers. As such, these threats are all broadly correlated with the human influence on the environment. Therefore, we used the Global Human Influence Index (GHII) map (Wildlife Conservation Society - WCS & Center for International Earth Science Information Network - CIESIN - Columbia University 2005) as a broad and combined proxy for the above five threats. The GHII is a combined map at the resolution of 1 km created from the integration of nine global layers on human population pressure (such as population density), human land-use and infrastructures (such as built-up areas, different land covers and land-uses) as well as human accessibility (such as transport networks and coastlines). As such, the GHII represents well areas of high potential risk to vultures due to high density of power lines and human disturbance (as a proof, compare the GHII map used here with maps T3 on electrocution threat and T1 on exposure to people in Africa available at: [www.habitatinfo.com/vultures/maps](http://www.habitatinfo.com/vultures/maps/T3_electrocution.pdf)). Conversely, the connection between GHII and decline in food availability or habitat degradation stems from the well reported fact that human land-uses, particularly under intensive production regimes, cause habitat degradation (Imhoff et al. 2004; Vitousek et al. 1997). This occurs when resources are subtracted, such as the loss of large trees used by vultures for nesting, or carrion availability is reduced through depletion of wild herbivores and/or sanitary disposal of dead domestic livestock (Buechley & Şekercioğlu 2016; Donázar et al. 2009).

*Treatment of expert opinion information*

We used the expert opinion information provided in the Multi Species Action Plan to conserve African-Eurasian vultures (Botha et al. 2017) whereby regional panels of local and international experts have gathered in order to identify and rank the threats to vultures across regions of Africa and Eurasia. Threats have been identified and their priority has been ranked within eight distinct geographic regions (hereafter regions) covering the distributions of vultures across Africa and Eurasia (see Botha et al. 2017; and Appendix S13). Each threat, including unintentional and intentional poisoning, collision with energy infrastructures (such as wind turbines), electrocution, human disturbance, habitat degradation (including the loss of nesting trees) and decline in food availability, has been ranked along a scale from critical to high, medium and low threat. In order to incorporate this information into our spatial analyses, we matched the threats as listed in Botha et al. (2017) with each of the spatial layers acting as a proxy for threats as detailed above. Intentional and unintentional poisoning, as well as collision with wind energy infrastructures, were directly matched with the respective threat as listed in Botha et al. (2017). Conversely, the GHII layer available for this study was assumed to represent, and thus matched to, the following threats as listed in Botha et al. (2017): Collision with, and electrocution on, energy infrastructures (mainly powerlines), human disturbance, habitat degradation (including the loss of nesting trees) and decline in food availability. We then assigned a value of 2 to a specific threat in a particular region which was assessed by experts as being critical (Fig. S8; and Botha et al. 2017). Similarly, threats assessed to be high in a specific region were given a value of 1.5. All threats assessed to be of medium to low priority were given a score of 1 (see Appendix S14). To derive a single score by region for the GHII, which is a proxy for multiple threats assessed by experts (see above), we assigned the GHII a value based on the sum of the related threats assessed by experts in the specific region. While the values we assigned to each ranked threat priority are somewhat arbitrary, we believe they represent an appropriate balance whereby a threat assessed to be of critical priority in one region would receive double the weight of a medium to low priority threat. At the same time, the difference between the values assigned to the critical and low threat priorities is limited enough that would not lead to a situation whereby a threat would be overestimated in the regions where it was assessed by experts to be of critical priority, and underestimated in the opposite case.

Finally, we incorporated the above expert knowledge on the intensity of each threat in each region into the four spatial maps intended as proxies for the threats. In doing so, we multiplied the value of the pixels of each the four original threat layers (unintentional and intentional poisoning, wind energy, GHII; see above) by the relative value for the associated threat in the specific region as reported in Appendix S14 and as shown in Appendix S13. The resulting four spatial layers would ultimately represent the spatial distribution of the threats weighted by their priority as assessed by experts. All the four spatial layers were then rescaled to fit the values between zero and one.

*Uncertainty and sensitivity analyses*

In order to assess the sensitivity of the main results to the uncertainty associated to the data, it was necessary to obtain a measure of uncertainty for the layers used in the prioritization analysis.

For the 15 vulture distributions, we derived the uncertainty measure around the average probability of occurrence of each species directly from the ensemble approach used for the SDMs (Urban et al. 2016). We used the standard deviation of the predictions from the ensemble of models to create maps of prediction uncertainty. Similarly, for the unintentional poisoning layer, uncertainty was calculated as the pixel-wise standard deviation of values from the datasets originally used to create this layer (see details above). For the wind energy layer, uncertainty was calculated as the pixel-wise standard deviation of values from the unrestricted wind potential layer (used in main analyses) and the cost-restricted wind energy potential layer, available from Pogson et al. (2013).

It was not possible to obtain spatial layers of uncertainty for the intentional poisoning or the GHII layers as these were not calculated based on averaging procedures from which a variance could be used, or alternative layers were not available. However, the intentional poisoning layer was validated with empirical and independent data (see Appendix S10). Moreover, regarding the GHII layer, this is assumed to be associated with low uncertainty overall given the strong correlation between this and other similar types of maps showing the impact of human influence (compare the layer used here with alternative ones, such as the human footprint map by Venter et al. (2016)).

In order to assess the robustness of the main priority map shown in Fig. 1 of the main manuscript, we incorporated the uncertainty in the SDMs for the 15 vulture species, as well as that in the wind and unintentional poisoning (see above), directly into a prioritization analysis using the distribution discounting tool in Zonation (Moilanen et al. 2006). Distribution discounting is a method for including uncertainty in a conservation prioritization. This method is particularly suitable for prioritization exercises carried at the regional or higher level, as is the case of this study (Moilanen et al. 2006). In brief, distribution discounting helps finding the most robust solution to achieve a conservation goal given a level of uncertainty in the distributions of a set of features (Moilanen et al. 2006). The highest ranking sites will be those that have a high conservation value and low uncertainty. The uncertainty specifies a set of bound that expand around the nominal estimate of a feature as a function of an uncertainty parameter α. The uncertainty is subtracted from the nominal estimate. For the analyses we set the uncertainty parameter to 0.5 and 1, which in turn subtracts 0.5 and 1 SD from the nominal estimates of the input layers of analysis. This exercise produced two outputs representing in turn the priorities obtained based on the 0.5 and 1 SD discounted distributions of the selected features (presented in Appendix S17 A and B). The priority values from these maps could then be compared with those of the main map (Fig. 1) both visually and quantitatively by means of a Pearson correlation.

Finally, we performed a sensitivity analysis by varying the weights assigned to each set of feature(s) used in the prioritization (Moilanen et al. 2014). We in turn increased the weights for a given set feature(s) by two and four times, while leaving the weights of all other features unchanged. At each iteration we extracted the proportion of each feature included within the top 30% fraction of the ranked landscape. This allowed us to quantify the impact of changing the weight of a feature on the ranking of that and the other features (see results of this sensitivity analysis to weights in Appendix S18).

*Reference list for Appendix S1:*

African wildlife poisoning database www.africanwildlifepoisoning.org. The Endangered Wildlife Trust and the Peregrine Fund, Available at: www.africanwildlifepoisoning.org.

Aiello-Lammens, M. E., R. A. Boria, A. Radosavljevic, B. Vilela, and R. P. Anderson. 2015. spThin: an R package for spatial thinning of species occurrence records for use in ecological niche models. Ecography **38**:541-545.

Barbet-Massin, M., W. Thuiller, and F. Jiguet. 2012. The fate of European breeding birds under climate, land-use and dispersal scenarios. Global Change Biology **18**:881-890.

Botha, A., J. Andevski, C. G. R. Bowden, M. Gudka, R. J. Safford, J. Tavares, and N. P. Williams. 2017. Multi-species Action Plan to Conserve African-Eurasian Vultures. Coordinating Unit of the CMS Raptors MOU. CMS Raptors MOU Technical Publication No. 5. CMS Technical Series No. xx, Abu Dhabi, United Arab Emirates.

Breiman, L. 2001. Random Forests. Machine Learning **45**:5-32.

Buechley, E. R., and Ç. H. Şekercioğlu. 2016. The avian scavenger crisis: Looming extinctions, trophic cascades, and loss of critical ecosystem functions. Biological Conservation **198**:220-228.

Chamberlain, S. 2017. rgbif: Interface to the Global Biodiversity Information Facility 'API'. R package version 0.9.8. https://CRAN.R-project.org/package=rgbif.

Díaz, S., S. Lavorel, F. de Bello, F. Quétier, K. Grigulis, and T. M. Robson. 2007. Incorporating plant functional diversity effects in ecosystem service assessments. Proceedings of the National Academy of Sciences **104**:20684-20689.

Donázar, J. A., A. Margalida, M. Carrete, and J. A. Sanchez-Zapata. 2009. Too Sanitary for Vultures. Science **326**:664-664.

Elith, J., J. R. Leathwick, and T. Hastie. 2008. A working guide to boosted regression trees. Journal of Animal Ecology **77**:802-813.

Fick, S. E., and R. J. Hijmans. 2017. WorldClim 2: new 1-km spatial resolution climate surfaces for global land areas. International Journal of Climatology **37**:4302-4315.

Friedman, J. H. 2001. Greedy function approximation: A gradient boosting machine. Ann. Statist. **29**:1189-1232.

GBIF.org (15 June 2017) GBIF Occurrence Download <https://doi.org/10.15468/dl.ftatsb>

GBIF.org (15 June 2017) GBIF Occurrence Download <https://doi.org/10.15468/dl.wt1mil>

GBIF.org (15 June 2017) GBIF Occurrence Download <https://doi.org/10.15468/dl.gkjgbe>

GBIF.org (27 January 2017) GBIF Occurrence Download <https://doi.org/10.15468/dl.agn2vj>

GBIF.org (27 January 2017) GBIF Occurrence Download <https://doi.org/10.15468/dl.ncksip>

GBIF.org (27 January 2017) GBIF Occurrence Download <https://doi.org/10.15468/dl.1sisi0>

GBIF.org (27 January 2017) GBIF Occurrence Download <https://doi.org/10.15468/dl.amkqmz>

GBIF.org (27 January 2017) GBIF Occurrence Download <https://doi.org/10.15468/dl.jgeo8n>

GBIF.org (27 January 2017) GBIF Occurrence Download <https://doi.org/10.15468/dl.k8kggk>

GBIF.org (27 January 2017) GBIF Occurrence Download <https://doi.org/10.15468/dl.jo3lv9>

GBIF.org (27 January 2017) GBIF Occurrence Download <https://doi.org/10.15468/dl.8hxzdt>

GBIF.org (27 January 2017) GBIF Occurrence Download <https://doi.org/10.15468/dl.jpwc8w>

GBIF.org (27 January 2017) GBIF Occurrence Download <https://doi.org/10.15468/dl.fbnmwp>

GBIF.org (27 January 2017) GBIF Occurrence Download <https://doi.org/10.15468/dl.9mtdbc>

GBIF.org (27 January 2017) GBIF Occurrence Download <https://doi.org/10.15468/dl.rpxdtl>

Hofierka, J., H. Mitášová, and M. Neteler. 2009. Chapter 17 Geomorphometry in GRASS GIS. Pages 387-410 in T. Hengl, and H. I. Reuter, editors. Developments in Soil Science. Elsevier.

Imhoff, M. L., L. Bounoua, T. Ricketts, C. Loucks, R. Harriss, and W. T. Lawrence. 2004. Global patterns in human consumption of net primary production. Nature **429**:870-873.

Jarvis, A., H. I. Reuter, A. Nelson, and E. Guevara. 2008. Hole-filled SRTM for the globe Version 4, available from the CGIAR-CSI SRTM 90m Database (http://srtm.csi.cgiar.org).

Jones, K. E., J. Bielby, M. Cardillo, S. A. Fritz, J. O'Dell, C. D. L. Orme, K. Safi, W. Sechrest, E. H. Boakes, C. Carbone, C. Connolly, M. J. Cutts, J. K. Foster, R. Grenyer, M. Habib, C. A. Plaster, S. A. Price, E. A. Rigby, J. Rist, A. Teacher, O. R. P. Bininda-Emonds, J. L. Gittleman, G. M. Mace, and A. Purvis. 2009. PanTHERIA: a species-level database of life history, ecology, and geography of extant and recently extinct mammals. Ecology **90**:2648-2648.

Mateo-Tomas, P., P. P. Olea, I. S. Sanchez-Barbudo, and R. Mateo. 2012. Alleviating human-wildlife conflicts: identifying the causes and mapping the risk of illegal poisoning of wild fauna. Journal of Applied Ecology **49**:376-385.

McCullagh, P., and J. A. Nelder 1989. Generalized Linear Models, Second Edition. Taylor & Francis.

Miller, J. R. B. 2015. Mapping attack hotspots to mitigate human–carnivore conflict: approaches and applications of spatial predation risk modeling. Biodiversity and Conservation **24**:2887-2911.

Moilanen, A., F. M. Pouzols, L. Meller, V. Veach, A. Arponen, J. Leppänen, and H. Kujala. "Zonation–Spatial Conservation Planning Methods and Software. Version 4. User Manual." University of Helsinki: Finland (2014).

Moilanen A, B.A. Wintle, J. Elith, M. Burgman. 2006. Uncertainty Analysis for Regional-Scale Reserve Selection. Conservation Biology **20**:1688-1697.

Muscarella, R., P. J. Galante, M. Soley-Guardia, R. A. Boria, J. M. Kass, M. Uriarte, and R. P. Anderson. 2014. ENMeval: An R package for conducting spatially independent evaluations and estimating optimal model complexity for Maxent ecological niche models. Methods in Ecology and Evolution **5**:1198-1205.

Ogada, D., A. Botha, and P. Shaw. 2016a. Ivory poachers and poison: drivers of Africa's declining vulture populations. Oryx **50**:593-596.

Ogada, D., P. Shaw, R. L. Beyers, R. Buij, C. Murn, J. M. Thiollay, C. M. Beale, R. M. Holdo, D. Pomeroy, N. Baker, S. C. Kruger, A. Botha, M. Z. Virani, A. Monadjem, and A. R. E. Sinclair. 2016b. Another Continental Vulture Crisis: Africa's Vultures Collapsing toward Extinction. Conservation Letters **9**:89-97.

Pearce-Higgins, J. W., and R. E. Green 2014. Birds and Climate Change - Impacts and Conservation Responses. Cambridge University Press, Cambridge, UK.

Pogson, M., A. Hastings, and P. Smith. 2013. How does bioenergy compare with other land-based renewable energy sources globally? Global Change Biology Bioenergy **5**:513-524.

R Core Development Team 2016. R: A language and environment for statistical computing. Version 3.4.1. Available from https://www.r-project.org/

REN21. 2017. Renewables 2017 Global Status Report in R. Secretariat, editor, Paris.

Robinson, T. P., G. R. W. Wint, G. Conchedda, T. P. Van Boeckel, V. Ercoli, E. Palamara, G. Cinardi, L. D'Aietti, S. I. Hay, and M. Gilbert. 2014. Mapping the Global Distribution of Livestock. PLOS ONE **9**:e96084.

Santangeli, A., V. Arkumarev, N. Rust, and M. Girardello. 2016a. Understanding, quantifying and mapping the use of poison by commercial farmers in Namibia – Implications for scavengers' conservation and ecosystem health. Biological Conservation **204,** 205-211.

Santangeli, A., S. H. M. Butchart, M. Pogson, A. Hastings, P. Smith, M. Girardello, and A. Moilanen. 2018. Mapping the global potential exposure of soaring birds to terrestrial wind energy expansion. Ornis Fennica **95**:1-14.

Santangeli, A., E. Di Minin, T. Toivonen, M. Pogson, A. Hastings, P. Smith, and A. Moilanen. 2016b. Synergies and trade-offs between renewable energy expansion and biodiversity conservation - a cross-national multifactor analysis. Global Change Biology Bioenergy **8**:1191-1200.

Santangeli, A., T. Toivonen, F. M. Pouzols, M. Pogson, A. Hastings, P. Smith, and A. Moilanen. 2016c. Global change synergies and trade-offs between renewable energy and biodiversity. Global Change Biology Bioenergy **8**:941-951.

Simmons, R. E., C. J. Brown, and J. Kemper 2015. Birds to watch in Namibia: red, rare and endemic species. Ministry of Environment and Tourism and Namibia Nature Foundation, Windhoek, Namibia.

Urban, M. C., G. Bocedi, A. P. Hendry, J.-B. Mihoub, G. Pe’er, A. Singer, J. R. Bridle, L. G. Crozier, L. De Meester, W. Godsoe, A. Gonzalez, J. J. Hellmann, R. D. Holt, A. Huth, K. Johst, C. B. Krug, P. W. Leadley, S. C. F. Palmer, J. H. Pantel, A. Schmitz, P. A. Zollner, and J. M. J. Travis. 2016. Improving the forecast for biodiversity under climate change. Science **353**.

Wildlife Conservation Society - WCS, and Center for International Earth Science Information Network - CIESIN - Columbia University. 2005. Last of the Wild Project, Version 2, 2005 (LWP-2): Global Human Influence Index (HII) Dataset (Geographic). NASA Socioeconomic Data and Applications Center (SEDAC), Palisades, NY.

Wilman, H., J. Belmaker, J. Simpson, C. de la Rosa, M. M. Rivadeneira, and W. Jetz. 2014. EltonTraits 1.0: Species-level foraging attributes of the world's birds and mammals. Ecology **95**:2027-2027.

Venter O, et al. 2016. Global terrestrial Human Footprint maps for 1993 and 2009. Scientific Data **3**:160067.

Vitousek, P. M., H. A. Mooney, J. Lubchenco, and J. M. Melillo. 1997. Human domination of Earth's ecosystems. Science **277**:494-499.

Woodroffe, R., S. J. Thirgood, and A. Rabinowitz 2005. People and wildlife: conflict or co-existance? Cambridge University Press, Cambridge, UK.

**Appendix S2:** The number of vulture occurrence data used for the species distribution modeling after applying the filters (see Appendix 1 extended methods) on the overall GBIF and ARDB observation data.

| **Species** | **Number of used records** |
| --- | --- |
| Aegypius monachus | 285 |
| Gypaetus barbatus | 443 |
| Gyps africanus | 1327 |
| Gyps bengalensis | 207 |
| Gyps coprotheres | 378 |
| Gyps fulvus | 636 |
| Gyps himalayensis | 213 |
| Gyps indicus | 111 |
| Gyps rueppelli | 383 |
| Gyps tenuirostris | 34 |
| Necrosyrtes monachus | 840 |
| Neophron percnopterus | 982 |
| Sarcogyps calvus | 70 |
| Torgos tracheliotos | 567 |
| Trigonoceps occipitalis | 316 |

**Appendix S3:** List of the original 35 land-cover categories derived European Space Agency 2012 land-cover map ([www.esa-landcover-cci.org/](http://www.esa-landcover-cci.org/)) and their aggregation in to seven broad and homogenous classes of land-cover used for the species distribution modeling of vulture distributions.

| **Original land-cover categories** | **Pooled land-cover categories** |
| --- | --- |
| Cropland, rainfed | agricultural |
| Herbaceous cover | grassland |
| Tree or shrub cover | woodland |
| Cropland,irrigated or post-flooding | agricultural |
| Mosaic cropland (>50%) / natural vegetation (tree, shrub, herbaceous) | agricultural |
| Tree cover, broadleaved, evergreen, closed to open (>15%) | woodland |
| Tree cover, broadleaved, deciduous, closed to open (>15%) | woodland |
| Tree cover, broadleaved, deciduous, closed (>40%) | woodland |
| Tree cover, broadleaved, deciduous, open (15‐40%) | woodland |
| Tree cover, needleleaved, evergreen, closed to open (>15%) | woodland |
| Tree cover, needleleaved, evergreen, closed (>40% | woodland |
| Tree cover, needleleaved, evergreen, open (15‐40%) | woodland |
| Tree cover, needleleaved, deciduous, closed to open (>15%) | woodland |
| Tree cover, needleleaved, deciduous, closed (>40%) | woodland |
| Tree cover, needleleaved, deciduous, open (15‐40%) | woodland |
| Tree cover, mixed leaf type (broadleaved and needleleaved) | woodland |
| Mosaic tree and shrub (>50%) / herbaceous cover (<50%) | mosaic |
| Mosaic herbaceous cover (>50%) / tree and shrub (<50%) | mosaic |
| Shrubland | shrubland |
| Evergreen shrubland | shrubland |
| Deciduous shrubland | shrubland |
| Grassland | grassland |
| Sparse vegetation (tree, shrub, herbaceous cover) (<15%) | grassland |
| Sparse shrub (<15%) | shrubland |
| Sparse herbaceous cover (<15%) | grassland |
| Urban areas | urban |
| Bare areas | bare |
| Consolidated bare areas | bare |
| Unconsolidated bare areas | bare |

**Appendix S4:** Validation metrics for the species distribution models (SDM) for each of the 15 vulture species considered for this study. The Figure shows values for the Area Under the receiver operating characteristic Curve (AUC; values of AUC > 0.70 indicate a fair discriminatory capacity according to the guidelines proposed by Swets (1988)) and the maximum True Skill Statistic (TSS; values of TSS > 0.4 indicate good discriminatory capacity) for the SDM of each vulture species used for the prioritization analysis. Only models with a fair performance according the ROC values were retained to create the final ensemble (AUC > 0.70). These were chosen on the basis of the guidelines provided by Swets 1988 (Measuring the accuracy of diagnostic systems. *Science*, *240*(4857), 1285-1293).


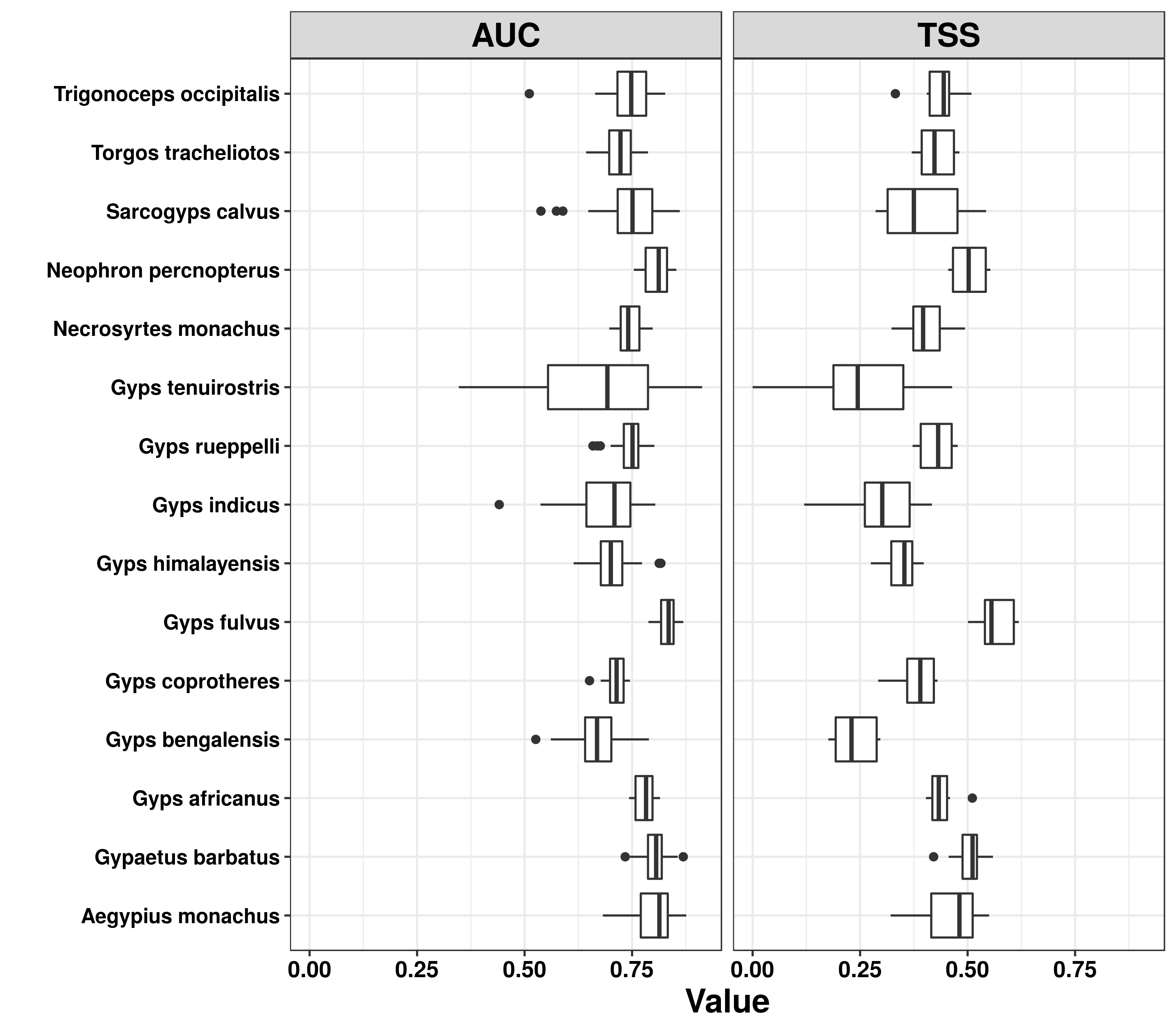


**Appendix S5:** The list of 107 carnivore species found within the range of the vulture distributions focus of this study. Species are listed with their latin name along with their body mass in kg obtained from Wilman et al*.* (2014). The third column from the left depicts the matching between each carnivore and the selected livestock prey category/categories based on the outcomes of the literature review. These outcomes are shown in the fourth to sixth columns from the left (poultry, small stock and large stock), whereby any incidence of predation by each carnivore on the selected livestock class is depicted by the value of 1. Empty cells depict lack of information or information not retrieved by our search on predation incidences of the carnivore on the specific livestock class. The rightmost column indicates the code for the source where the incidence was reported, which is listed in full below the Table.

| **Latin name** | **Mass (Kg)** | **Livestock prey** | **Poultry** | **Small stock** | | **Large stock** | | **Reference** | |  |
| --- | --- | --- | --- | --- | --- | --- | --- | --- | --- | --- |
| Mustela nivalis | 0,1 | poultry |  | |  | |  | |  | |
| Mustela erminea | 0,12 | poultry |  | |  | |  | |  | |
| Mustela altaica | 0,17 | poultry |  | |  | |  | |  | |
| Mustela kathiah | 0,21 | poultry |  | |  | |  | |  | |
| Poecilogale albinucha | 0,34 | poultry |  | |  | |  | |  | |
| Mustela subpalmata | 0,35 | poultry |  | |  | |  | |  | |
| Mustela sibirica | 0,41 | poultry |  | |  | |  | |  | |
| Mustela lutreola | 0,44 | poultry |  | |  | |  | |  | |
| Vormela peregusna | 0,45 | poultry |  | |  | |  | |  | |
| Poiana leightoni | 0,5 | poultry |  | |  | |  | |  | |
| Poiana richardsonii | 0,5 | poultry |  | |  | |  | |  | |
| Prionodon pardicolor | 0,51 | poultry |  | |  | |  | |  | |
| Herpestes javanicus | 0,75 | poultry | 1 | |  | |  | | 1 | |
| Melogale moschata | 0,81 | poultry |  | |  | |  | |  | |
| Mustela putorius | 0,92 | poultry |  | |  | |  | |  | |
| Genetta servalina | 1,05 | poultry |  | |  | |  | |  | |
| Vulpes zerda | 1,1 | poultry |  | |  | |  | |  | |
| Martes zibellina | 1,13 | poultry |  | |  | |  | |  | |
| Felis nigripes | 1,3 | poultry |  | |  | |  | |  | |
| Ictonyx striatus | 1,3 | poultry |  | |  | |  | |  | |
| Martes martes | 1,3 | poultry |  | |  | |  | |  | |
| Herpestes edwardsii | 1,3 | poultry | 1 | |  | |  | | 1 | |
| Prionailurus rubiginosus | 1,38 | poultry |  | |  | |  | |  | |
| Genetta thierryi | 1,4 | poultry |  | |  | |  | |  | |
| Mustela strigidorsa | 1,5 | poultry |  | |  | |  | |  | |
| Martes foina | 1,54 | poultry |  | |  | |  | |  | |
| Bdeogale crassicauda | 1,55 | poultry |  | |  | |  | |  | |
| Bdeogale omnivora | 1,55 | poultry |  | |  | |  | |  | |
| Paracynictis selousi | 1,64 | poultry |  | |  | |  | |  | |
| Genetta abyssinica | 1,65 | poultry |  | |  | |  | |  | |
| Genetta angolensis | 1,65 | poultry |  | |  | |  | |  | |
| Mustela eversmanii | 1,68 | poultry |  | |  | |  | |  | |
| Melogale personata | 1,7 | poultry |  | |  | |  | |  | |
| Herpestes smithii | 1,78 | poultry |  | |  | |  | |  | |
| Genetta genetta | 1,8 | poultry |  | |  | |  | |  | |
| Genetta bourloni | 1,82 | poultry |  | |  | |  | |  | |
| Genetta pardina | 1,82 | poultry |  | |  | |  | |  | |
| Genetta poensis | 1,82 | poultry |  | |  | |  | |  | |
| Martes flavigula | 1,84 | poultry |  | |  | |  | |  | |
| Herpestes urva | 1,86 | poultry | 1 | |  | |  | | 1 | |
| Genetta cristata | 1,86 | poultry |  | |  | |  | |  | |
| Herpestes fuscus | 1,98 | poultry |  | |  | |  | |  | |
| Nandinia binotata | 2 | poultry AND small stock | 1 | | 1 | |  | | 2 | |
| Martes gwatkinsii | 2,04 | poultry AND small stock |  | |  | |  | |  | |
| Ictonyx libycus | 2,06 | poultry AND small stock |  | |  | |  | |  | |
| Genetta johnstoni | 2,23 | poultry AND small stock |  | |  | |  | |  | |
| Genetta maculata | 2,23 | poultry AND small stock |  | |  | |  | |  | |
| Genetta tigrina | 2,23 | poultry AND small stock |  | |  | |  | |  | |
| Arctogalidia trivirgata | 2,25 | poultry AND small stock |  | |  | |  | |  | |
| Vulpes corsac | 2,4 | poultry AND small stock |  | |  | |  | |  | |
| Bdeogale jacksoni | 2,5 | poultry AND small stock |  | |  | |  | |  | |
| Bdeogale nigripes | 2,5 | poultry AND small stock |  | |  | |  | |  | |
| Felis margarita | 2,52 | poultry AND small stock |  | |  | |  | |  | |
| Herpestes vitticollis | 2,58 | poultry AND small stock |  | |  | |  | |  | |
| Vulpes pallida | 2,8 | poultry AND small stock |  | |  | |  | |  | |
| Pardofelis marmorata | 2,85 | poultry AND small stock |  | |  | |  | |  | |
| Viverricula indica | 2,91 | poultry AND small stock |  | |  | |  | |  | |
| Genetta victoriae | 3 | poultry AND small stock |  | |  | |  | |  | |
| Herpestes naso | 3 | poultry AND small stock |  | |  | |  | |  | |
| Otocolobus manul | 3,05 | poultry AND small stock |  | |  | |  | |  | |
| Vulpes rueppellii | 3,25 | poultry AND small stock |  | |  | |  | |  | |
| Atilax paludinosus | 3,3 | poultry AND small stock |  | |  | |  | |  | |
| Prionailurus bengalensis | 3,3 | poultry AND small stock |  | |  | |  | |  | |
| Nyctereutes procyonoides | 4,04 | poultry AND small stock |  | |  | |  | |  | |
| Otocyon megalotis | 4,15 | poultry AND small stock |  | |  | |  | |  | |
| Paguma larvata | 4,3 | poultry AND small stock |  | |  | |  | |  | |
| Ailurus fulgens | 4,9 | poultry AND small stock |  | |  | |  | |  | |
| Vulpes ferrilata | 5 | poultry AND small stock |  | |  | |  | |  | |
| Felis silvestris | 5,1 | poultry AND small stock |  | |  | |  | |  | |
| Herpestes ichneumon | 5,17 | poultry AND small stock |  | |  | |  | |  | |
| Vulpes vulpes | 5,48 | poultry AND small stock | 1 | | 1 | |  | | 1, 3, 4 | |
| Arctonyx collaris | 6,36 | poultry AND small stock |  | |  | |  | |  | |
| Felis chaus | 7,39 | poultry AND small stock | 1 | |  | |  | | 1 | |
| Mellivora capensis | 8,5 | poultry AND small stock |  | |  | |  | |  | |
| Canis mesomelas | 8,5 | poultry AND small stock |  | | 1 | |  | | 5-8 | |
| Prionailurus viverrinus | 9,14 | poultry AND small stock |  | |  | |  | |  | |
| Viverra megaspila | 9,25 | poultry AND small stock |  | |  | |  | |  | |
| Lynx pardinus | 9,4 | poultry AND small stock | 1 | | 1 | |  | | 9 | |
| Viverra zibetha | 9,5 | poultry AND small stock |  | |  | |  | |  | |
| Arctictis binturong | 9,88 | poultry AND small stock |  | |  | |  | |  | |
| Canis simensis | 10 | poultry AND small stock AND large stock |  | |  | |  | |  | |
| Canis aureus | 10,35 | poultry AND small stock AND large stock |  | | 1 | | 1 | | 7, 10, 11 | |
| Caracal aurata | 10,65 | poultry AND small stock AND large stock |  | |  | |  | |  | |
| Catopuma temminckii | 11,5 | poultry AND small stock AND large stock |  | | 1 | |  | | 7 | |
| Civettictis civetta | 12 | poultry AND small stock AND large stock |  | |  | |  | |  | |
| Leptailurus serval | 12 | poultry AND small stock AND large stock |  | | 1 | |  | | 11 | |
| Meles leucurus | 13 | poultry AND small stock AND large stock |  | |  | |  | |  | |
| Meles meles | 13 | poultry AND small stock AND large stock |  | |  | |  | |  | |
| Caracal caracal | 13,75 | poultry AND small stock AND large stock |  | | 1 | |  | | 7, 8 | |
| Cuon alpinus | 14,17 | poultry AND small stock AND large stock |  | |  | | 1 | | 12 | |
| Gulo gulo | 17,01 | poultry AND small stock AND large stock |  | | 1 | |  | | 3, 7 | |
| Lynx lynx | 17,95 | poultry AND small stock AND large stock |  | | 1 | |  | | 3, 7, 13 | |
| Neofelis nebulosa | 19,68 | poultry AND small stock AND large stock |  | |  | |  | |  | |
| Lycaon pictus | 22,05 | poultry AND small stock AND large stock |  | | 1 | | 1 | | 7, 14 | |
| Canis lupus | 32,18 | poultry AND small stock AND large stock | 1 | | 1 | | 1 | | 7, 8, 12, 13, 15-17 | |
| Hyaena hyaena | 41,71 | poultry AND small stock AND large stock |  | | 1 | |  | | 7 | |
| Parahyaena brunnea | 42,98 | poultry AND small stock AND large stock |  | | 1 | | 1 | | 7 | |
| Panthera uncia | 44,17 | poultry AND small stock AND large stock |  | | 1 | | 1 | | 16, 18 | |
| Helarctos malayanus | 46,5 | poultry AND small stock AND large stock |  | |  | |  | |  | |
| Acinonyx jubatus | 46,7 | poultry AND small stock AND large stock |  | | 1 | | 1 | | 5, 7, 14 | |
| Panthera pardus | 52,04 | poultry AND small stock AND large stock |  | | 1 | | 1 | | 5, 7, 8, 11, 14, 16, 18, 19 | |
| Crocuta crocuta | 66,49 | poultry AND small stock AND large stock |  | | 1 | | 1 | | 5, 7, 8, 11, 14, 19 | |
| Ursus thibetanus | 77,5 | poultry AND small stock AND large stock |  | | 1 | | 1 | | 7, 12, 16, 18, 20 | |
| Ailuropoda melanoleuca | 108,4 | poultry AND small stock AND large stock |  | |  | |  | |  | |
| Panthera leo | 161,5 | poultry AND small stock AND large stock |  | | 1 | | 1 | | 5, 7, 8, 14, 19 | |
| Panthera tigris | 162,56 | poultry AND small stock AND large stock |  | | 1 | | 1 | | 7, 8, 18 | |
| Ursus arctos | 180,52 | poultry AND small stock AND large stock |  | | 1 | | 1 | | 7, 8, 13, 15, 16, 20 | |

**Reference list for Table:**

1. Biswas PK*, et al.* (2006) Causes of loss of Sonali chickens on smallholder households in Bangladesh. *Prev. Vet. Med.* 76(3-4):185-195.

2. Campbell M (2009) Proximity in a Ghanaian savanna: Human reactions to the African palm civet Nandinia binotata. *Singapore Journal of Tropical Geography* 30(2):220-231.

3. Warren JT, Mysterud I, & Lynnebakken T (2001) Mortality of lambs in free-ranging domestic sheep (Ovis aries) in northern Norway. *Journal of Zoology* 254(2):195-202.

4. Plumer L, Davison J, & Saarma U (2014) Rapid Urbanization of Red Foxes in Estonia: Distribution, Behaviour, Attacks on Domestic Animals, and Health-Risks Related to Zoonotic Diseases. *PLoS One* 9(12):15.

5. Muriuki MW, Ipara H, & Kiringe JW (2017) The cost of livestock lost to lions and other wildlife species in the Amboseli ecosystem, Kenya. *Eur. J. Wildl. Res.* 63(4):11.

6. Gusset M, Swarner MJ, Mponwane L, Keletile K, & McNutt JW (2009) Human-wildlife conflict in northern Botswana: livestock predation by Endangered African wild dog Lycaon pictus and other carnivores. *Oryx* 43(1):67-72.

7. Breitenmoser U*, et al.* (2005) Non-lethal techniques for reducing depredation. *People and wildlife: conflict or coexistence?*, eds Woodroffe R, Thirgood S, & Rabinowitz A (Cambridge University Press, Cambridge), pp 49 - 71.

8. Miller JRB (2015) Mapping attack hotspots to mitigate human-carnivore conflict: approaches and applications of spatial predation risk modeling. *Biodivers. Conserv.* 24(12):2887-2911.

9. Garrote G*, et al.* (2013) Human-felid conflict as a further handicap to the conservation of the critically endangered Iberian lynx. *Eur. J. Wildl. Res.* 59(2):287-290.

10. Yoram YT, Ashkenazi S, & Viner O (1995) Cattle predation by the golden jackal Canis-aureus in the Golan-Heights, Israel. *Biol. Conserv.* 73(1):19-22.

11. Atickem A, Williams S, Bekele A, & Thirgood S (2010) Livestock predation in the Bale Mountains, Ethiopia. *Afr. J. Ecol.* 48(4):1076-1082.

12. Li XY, Buzzard P, Chen YC, & Jiang XL (2013) Patterns of Livestock Predation by Carnivores: Human-Wildlife Conflict in Northwest Yunnan, China. *Environ. Manage.* 52(6):1334-1340.

13. Karlsson J & Johansson O (2010) Predictability of repeated carnivore attacks on livestock favours reactive use of mitigation measures. *Journal of Applied Ecology* 47(1):166-171.

14. Woodroffe R, Frank LG, Lindsey PA, Ranah S, & Romanach S (2007) Livestock husbandry as a tool for carnivore conservation in Africa's community rangelands: a case-control study. *Biodivers. Conserv.* 16(4):1245-1260.

15. Frank J & Eklund A (2017) Poor construction, not time, takes its toll on subsidised fences designed to deter large carnivores. *PLoS One* 12(4):10.

16. Ahmad S*, et al.* (2016) Carnivores' diversity and conflicts with humans in Musk Deer National Park, Azad Jammu and Kashmir, Pakistan. *Eur. J. Wildl. Res.* 62(5):565-576.

17. Torres RT & Fonseca C (2016) Perspectives on the Iberian wolf in Portugal: population trends and conservation threats. *Biodivers. Conserv.* 25(3):411-425.

18. Sangay T & Vernes K (2008) Human-wildlife conflict in the Kingdom of Bhutan: Patterns of livestock predation by large mammalian carnivores. *Biol. Conserv.* 141(5):1272-1282.

19. Kissui BM (2008) Livestock predation by lions, leopards, spotted hyenas, and their vulnerability to retaliatory killing in the Maasai steppe, Tanzania. *Animal Conservation* 11(5):422-432.

20. Chauhan NPS (2003) Human Casualties and Livestock Depredation by Black and Brown Bears in the Indian Himalaya, 1989-98. *Ursus* 14(1):84-87.

**Appendix S6:** Map showing the interaction between density of large stock (i.e. cattle and buffalo) and the average body mass of selected carnivores (see Appendix S5). The interaction was performed by means of multiplication of the values of large stock density and average value for the selected carnivore body mass (see Appendix S1 for further details). Areas with color towards red represent high values for the interaction, whereby high densities of large stock co-occur with selected carnivores of high average body mass. These areas are intended as proxies for high potential conflict between large livestock farming and carnivores as opposed to the green areas in the map. Areas beyond the range of any of the 15 vulture species focus of this study are shown in grey.


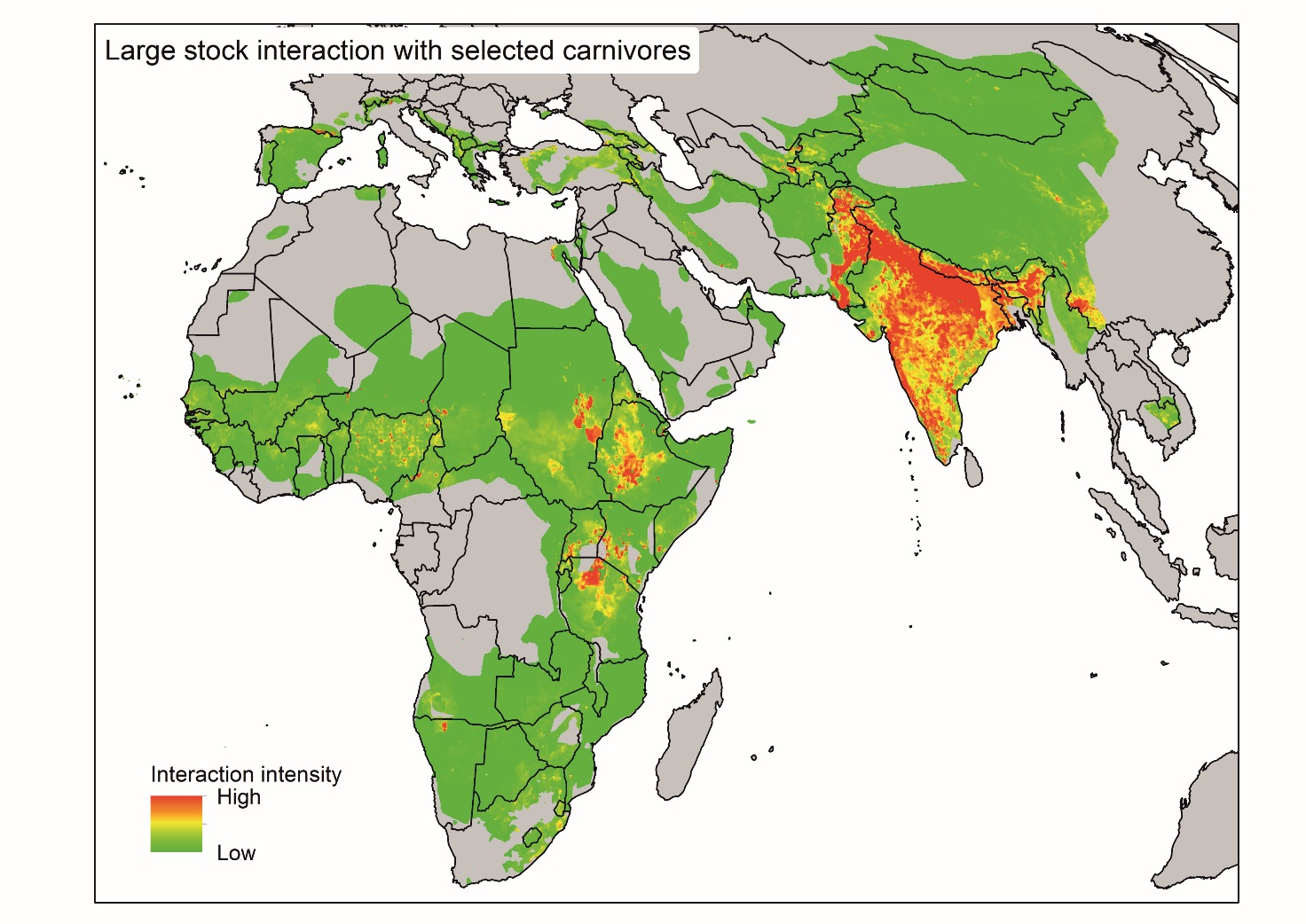


**Appendix S7:** Map showing the interaction between density of small stock (i.e. sheep, goat and pig) and the average body mass of selected carnivores (see Appendix S5). The interaction was performed by means of multiplication of the values of small stock density and average value for the selected carnivore body mass (see Appendix S1 for further details). Areas with color towards red represent high values for the interaction, whereby high densities of small stock co-occur with selected carnivores of high average body mass. These areas are intended as proxies for high potential conflict between small livestock farming and carnivores as opposed to the green areas in the map. Areas beyond the range of any of the 15 vulture species focus of this study are shown in grey.


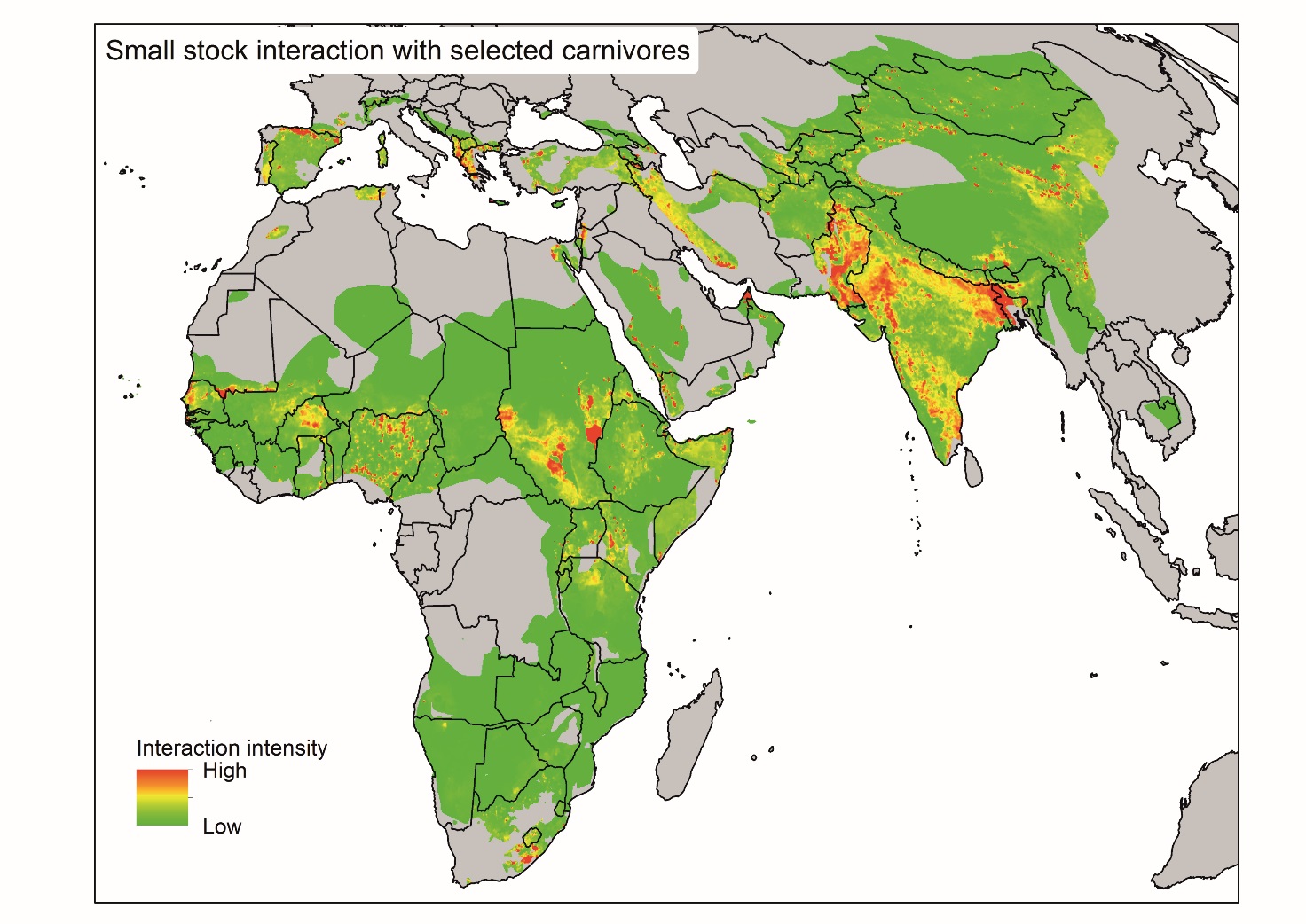


**Appendix S8:** Map showing the interaction between density of poultry (i.e. duck and chicken) and the average body mass of selected carnivores (see Appendix S5). The interaction was performed by means of multiplication of the values of poultry density and average value for the selected carnivore body mass (see Appendix S1 for further details). Areas with color towards red represent high values for the interaction, whereby high densities of poultry co-occur with selected carnivores of high average body mass. These areas are intended as proxies for high potential conflict between poultry farming and carnivores as opposed to the green areas in the map. Areas beyond the range of any of the 15 vulture species focus of this study are shown in grey.


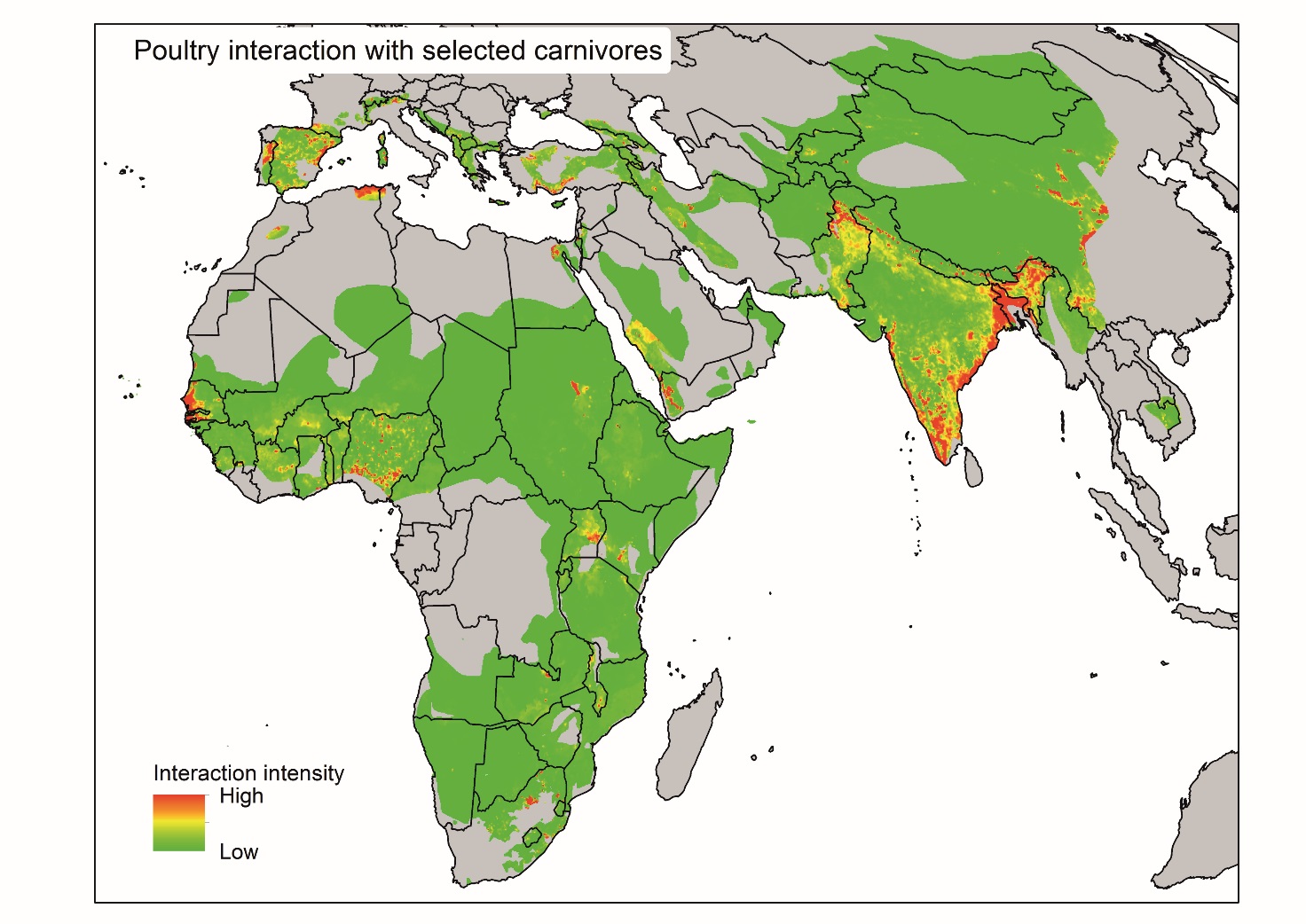


**Appendix S9:** The combined map showing the average of the three layers representing the interaction between the three livestock classes (poultry, large stock, small stock) and selected carnivores. To derive this map, the weighted average approach was used whereby the values of each of the three input maps were given a weight according to the average body mass (log transformed) of the livestock class in each of the three layers (see Appendix S1 for further details). Red areas in the map represent high values for the interaction. These areas are intended as having high potential conflict between livestock farming and carnivores as opposed to the green areas. Areas beyond the range of any of the 15 vulture species focus of this study are shown in grey.


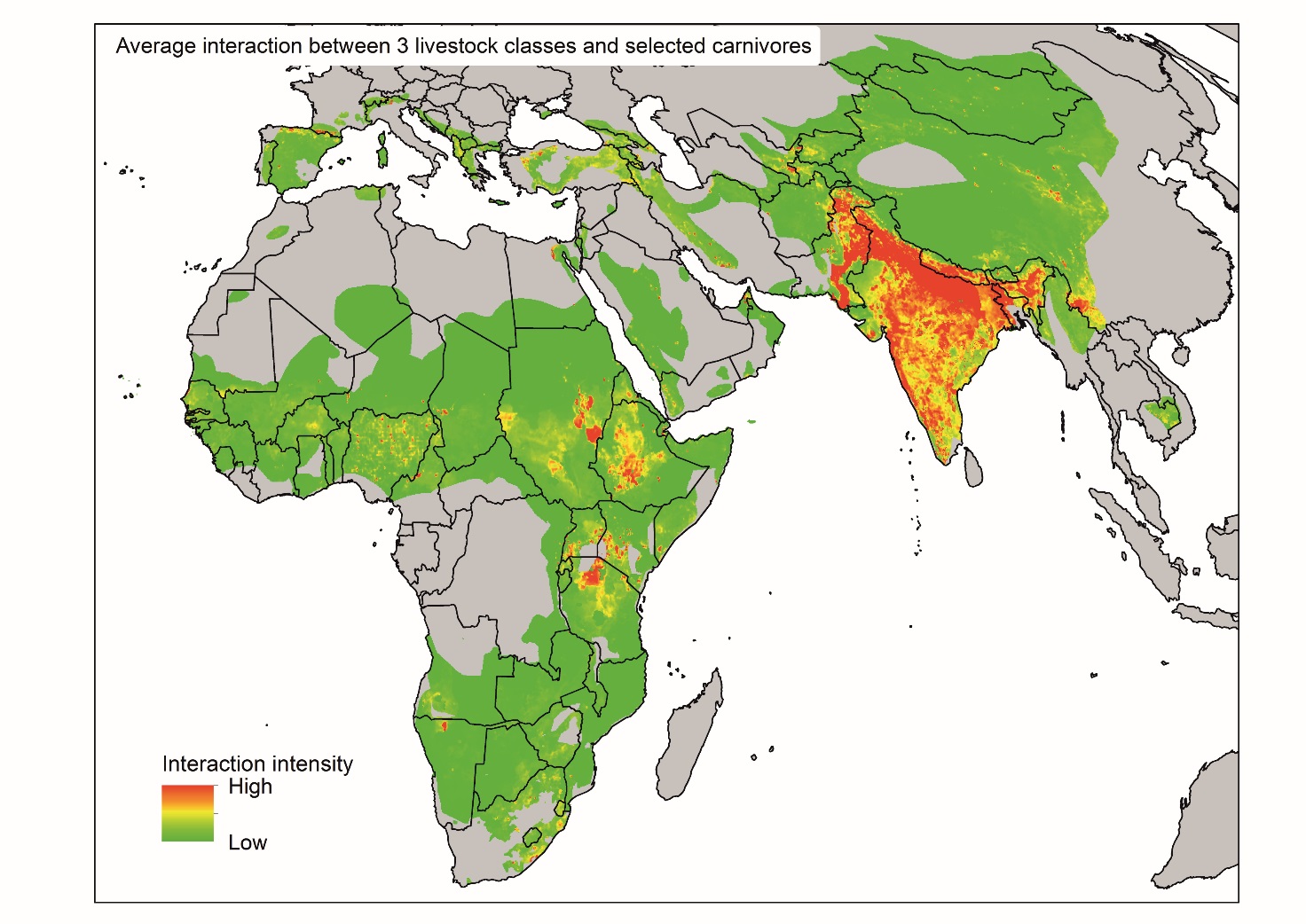


**Appendix S10:** The distribution of sentinel (intentional) and unintentional poisoning incidences reported in the African wildlife poisoning database overlaid on the distribution of priority areas for vulture conservation identified through a conservation planning exercise including the distributions of the 15 vulture species and both poisoning threat layers. Note that the database is not complete and nor unbiased, so that poisoning may be well reported in some areas, and less so in other areas. Hence, areas where poisoning incidences are not shown may be due to undetected and/or unreported cases rather than lack of poisoning per se.


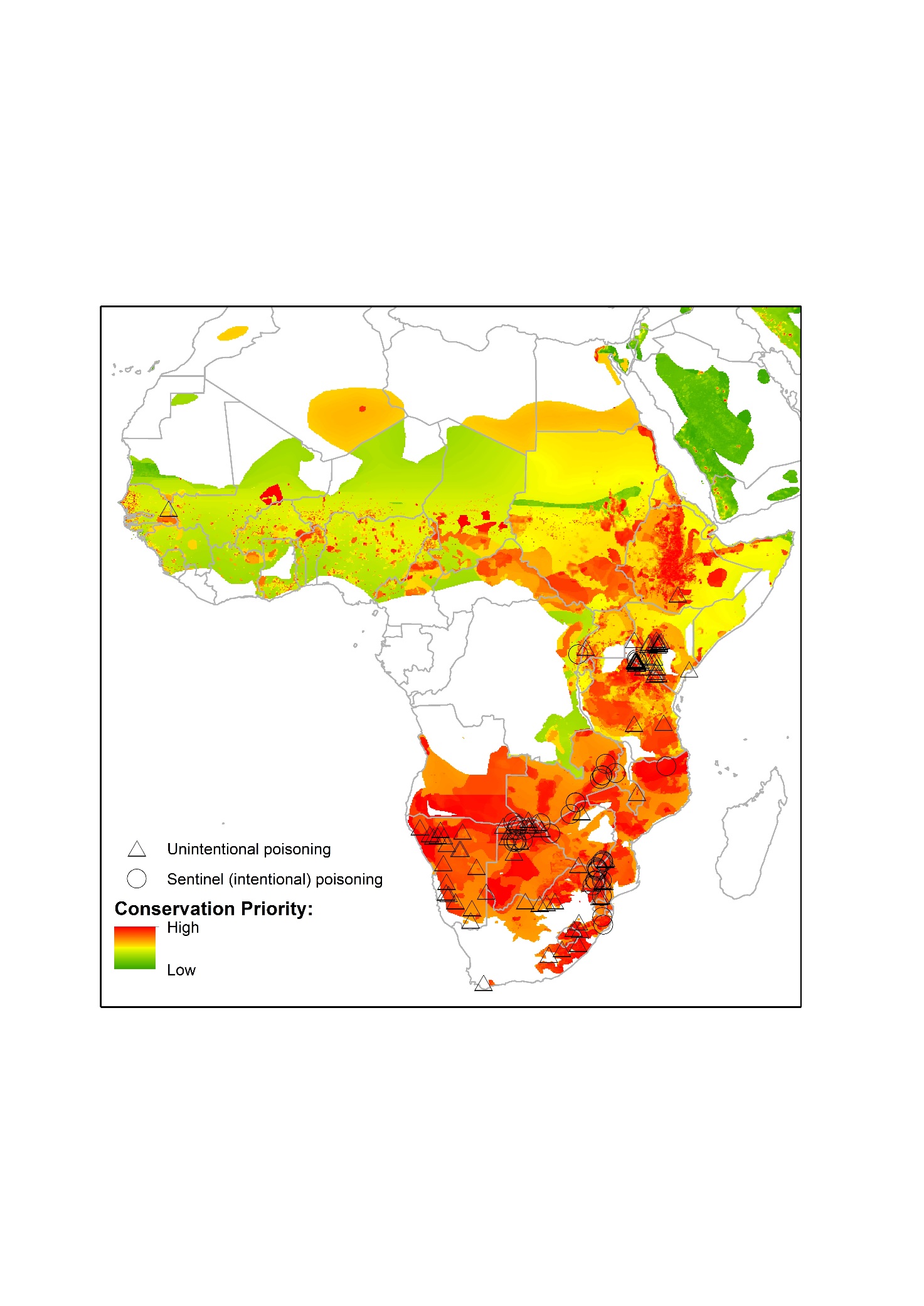


**Appendix S11:** The list of 72 species of herbivores and carnivores as identified for being potential target of intentional poisoning by poachers across South Saharan Africa. The weights reported in the relative column represent the relative number of incidences of intentional poisoning involving that species on the log (x+1) scale and as reported in the African wildlife poisoning database. Most species (n = 66) for which no incidences were reported in the poisoning database were given the weight of 0.3, i.e. the weight of the species reported in the database to have been target of intentional poisoning only once. This list of species and associated weights were used to derive the spatial layer of intentional poisoning (Appendix S10) across Africa by combining the distribution range of each of the species and their weights (see Appendix S1 for further details).

| **Latin Name** | **Weight** |
| --- | --- |
| Panthera leo | 1,4 |
| Loxodonta africana | 1,3 |
| Panthera pardus | 0,7 |
| Aepyceros melampus | 0,3 |
| Tragelaphus angasii | 0,3 |
| Hippopotamus amphibius | 0,3 |
| Cephalophus dorsalis | 0,3 |
| Cephalophus ogilbyi | 0,3 |
| Gazella spekei | 0,3 |
| Pelea capreolus | 0,3 |
| Cephalophus niger | 0,3 |
| Eudorcas albonotata | 0,3 |
| Eudorcas thomsonii | 0,3 |
| Gazella dorcas | 0,3 |
| Eudorcas rufifrons | 0,3 |
| Eudorcas tilonura | 0,3 |
| Ammodorcas clarkei | 0,3 |
| Redunca fulvorufula | 0,3 |
| Antidorcas marsupialis | 0,3 |
| Litocranius walleri | 0,3 |
| Nanger soemmerringii | 0,3 |
| Tragelaphus scriptus | 0,3 |
| Redunca redunca | 0,3 |
| Ammotragus lervia | 0,3 |
| Potamochoerus larvatus | 0,3 |
| Nanger granti | 0,3 |
| Cephalophus spadix | 0,3 |
| Redunca arundinum | 0,3 |
| Capra nubiana | 0,3 |
| Addax nasomaculatus | 0,3 |
| Cephalophus jentinki | 0,3 |
| Potamochoerus porcus | 0,3 |
| Phacochoerus aethiopicus | 0,3 |
| Nanger dama | 0,3 |
| Kobus vardonii | 0,3 |
| Cephalophus silvicultor | 0,3 |
| Tragelaphus spekii | 0,3 |
| Kobus kob | 0,3 |
| Beatragus hunteri | 0,3 |
| Tragelaphus imberbis | 0,3 |
| Phacochoerus africanus | 0,3 |
| Kobus megaceros | 0,3 |
| Capra walie | 0,3 |
| Damaliscus pygargus | 0,3 |
| Kobus leche | 0,3 |
| Damaliscus lunatus | 0,3 |
| Hippotragus leucophaeus | 0,3 |
| Oryx gazella | 0,3 |
| Alcelaphus buselaphus | 0,3 |
| Connochaetes gnou | 0,3 |
| Connochaetes taurinus | 0,3 |
| Hylochoerus meinertzhageni | 0,3 |
| Oryx dammah | 0,3 |
| Oryx beisa | 0,3 |
| Kobus ellipsiprymnus | 0,3 |
| Tragelaphus strepsiceros | 0,3 |
| Tragelaphus buxtoni | 0,3 |
| Hippotragus niger | 0,3 |
| Okapia johnstoni | 0,3 |
| Choeropsis liberiensis | 0,3 |
| Equus africanus | 0,3 |
| Hippotragus equinus | 0,3 |
| Equus zebra | 0,3 |
| Tragelaphus eurycerus | 0,3 |
| Equus quagga | 0,3 |
| Equus grevyi | 0,3 |
| Tragelaphus oryx | 0,3 |
| Syncerus caffer | 0,3 |
| Tragelaphus derbianus | 0,3 |
| Giraffa camelopardalis | 0,3 |
| Diceros bicornis | 0,3 |
| Ceratotherium simum | 0,3 |

**Appendix S12:** Map showing the areas of potentially high risk of intentional (sentinel) poisoning (red colors) across Africa obtained by deriving the per pixel average of an index, the species-specific weight of intentional poisoning (see Appendix S11), across all species occurring in each 10 km X 10 km pixel in the study region. Areas beyond the study region are shown in grey.


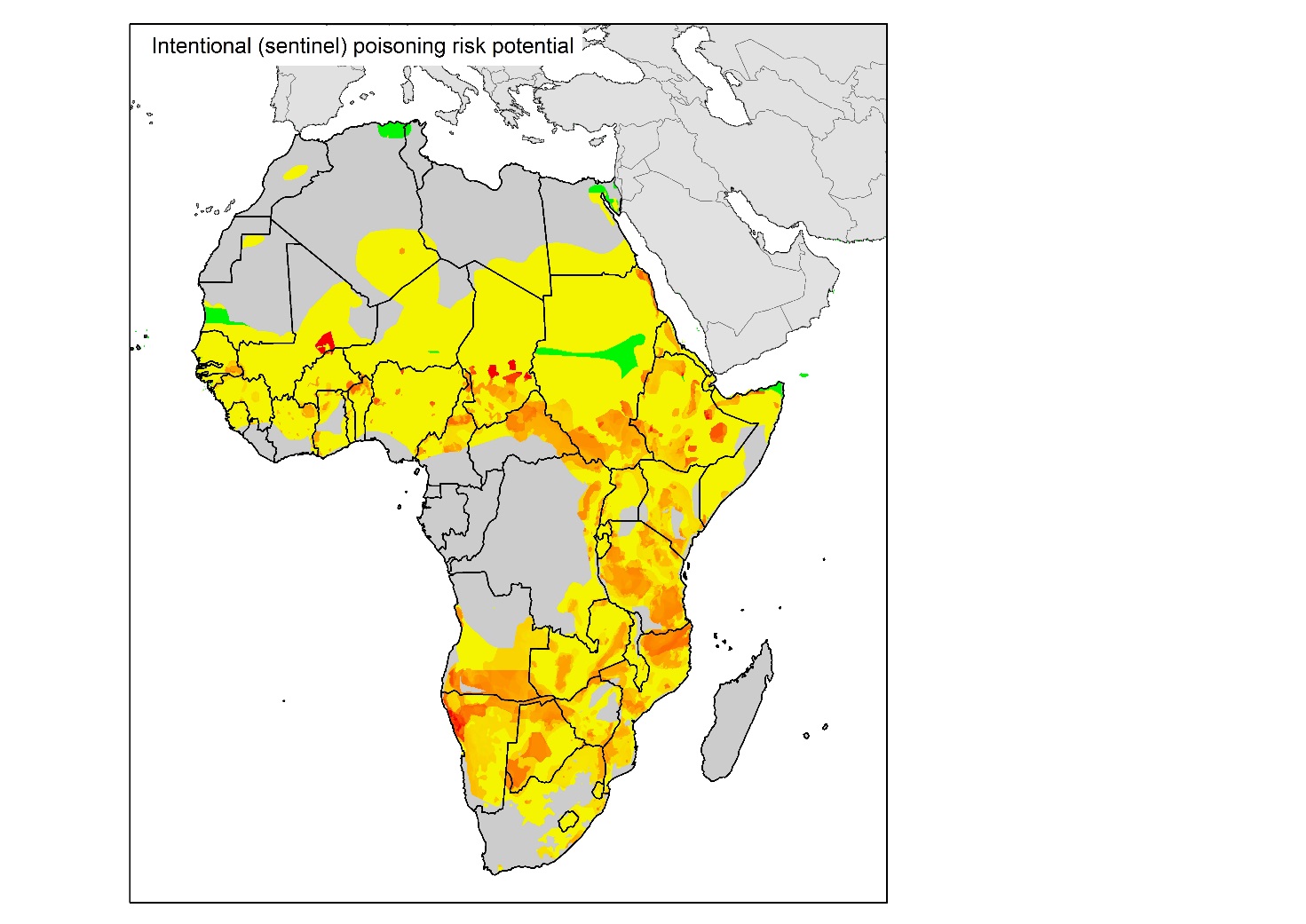


**Appendix S13:** The vulture study region across Africa and Eurasia divided in the eight regions (in a different colour) following Botha et al. 2017 (Multi-species Action Plan to Conserve African-Eurasian Vultures). Each threat to vultures was regionally ranked in its priority level by a regional panel of experts. Threat levels used were: Critical, high, medium and low, which are listed in the insets of the map next to the corresponding region (Botha et al. 2017). The present study included spatial layers as proxies for unintentional and intentional poisoning, collision with wind turbines, and a broad layer, namely the Global Human Influence Index, representing a proxy for threats such as collision and electrocution with powerlines, human disturbance, decline in food availability and habitat degradation. Threats for which a spatial layer was not available for the purpose of this study are omitted from the inset lists. Threat levels medium and low in the original document (Botha et al. 2017) were pooled in a single level. The value next to the threat level as assessed by Botha et al. (2017) depicts the weight that we assigned to each threat based on its original priority level (see Appendix S14). This value was later used to incorporate the expert knowledge information into the spatial layers prior to running the prioritization analyses (see Appendix S1 for further details).


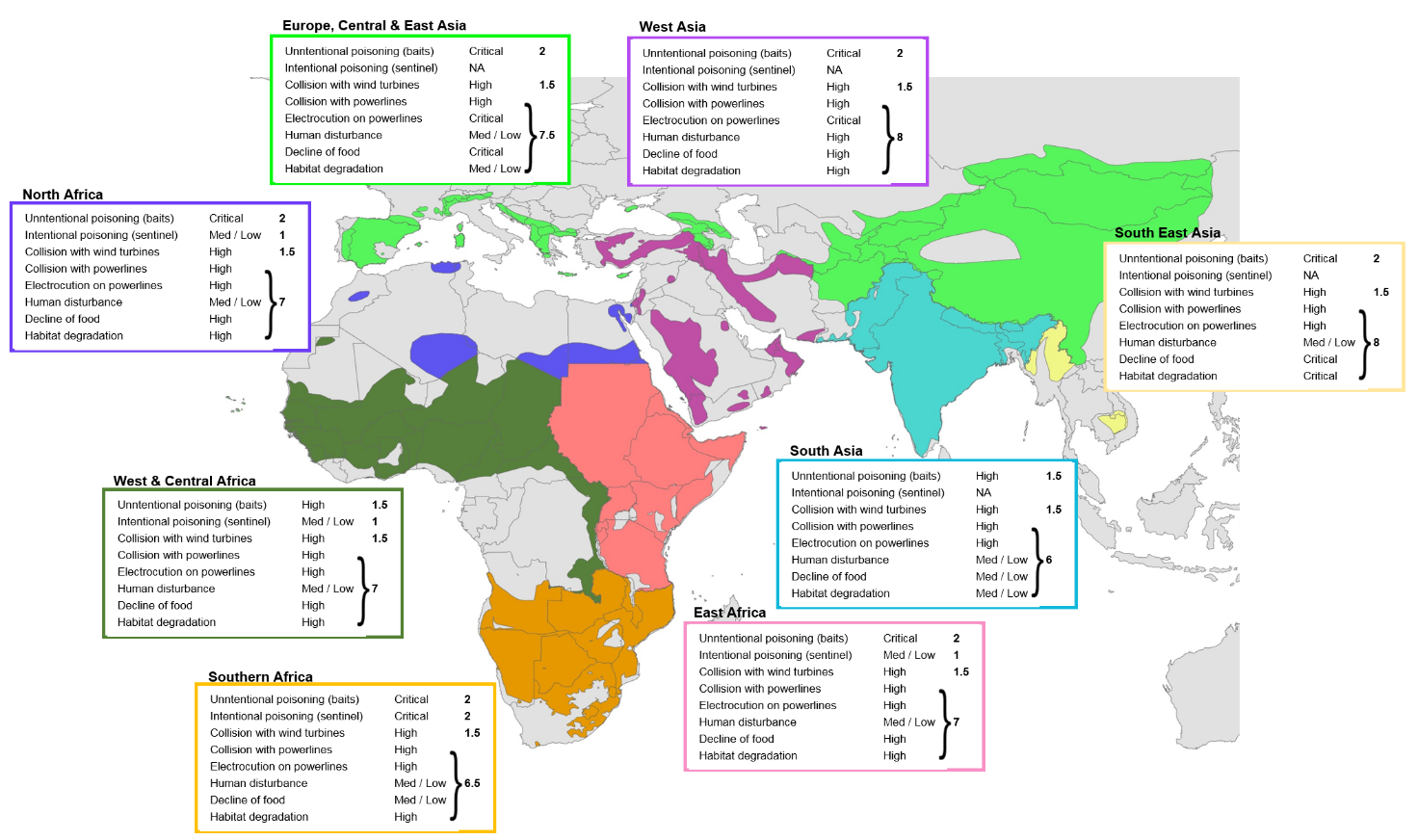


**Appendix S14:** The relative weight assigned to each of the four main threats (or proxy for multiple threats) to vultures across the eight regions in Africa and Eurasia where these threats have been assessed and ranked by panels of local and international experts as part of the development of the Multi Species Action Plan to conserve African-Eurasian vultures (Botha et al*.* 2017). The values for these threats have been used to refine the spatial layer of each of the four original threats used in this study by simply multiplying the pixel value of each threat by its relative region-specific weight as reported in this Table (see also Appendix S12). GHII depicts the Global Human Influence Index, which is here used as a combined proxy for multiple threats including collision with, and electrocution on, energy infrastructures (mainly powerlines), human disturbance, habitat degradation (including the loss of nesting trees) and decline in food availability. Intentional poisoning was given a value of zero beyond Africa where this threat has never been reported before.

| **Region:** | **Unintentional poisoning** | **Intentional (sentinel) poisoning** | **Wind energy** | **GHII** |
| --- | --- | --- | --- | --- |
| Southern Africa | 2 | 2 | 1,5 | 6,5 |
| West-Central Africa | 1,5 | 1 | 1,5 | 7 |
| East Africa | 2 | 1 | 1,5 | 7 |
| North Africa | 2 | 1 | 1,5 | 7 |
| Europe Central-East Asia | 2 | 0 | 1,5 | 7,5 |
| West Asia | 2 | 0 | 1,5 | 8 |
| South-East Asia | 2 | 0 | 1,5 | 8 |
| South Asia | 1,5 | 0 | 1,5 | 6 |

**Appendix S15:** List of explanatory variables considered for the model on the national share of the top 30% priority areas for vulture conservation in Africa and Eurasia. The description of each variable, along with the unit, year and source of the information are also given.

| **Factors name:** | **Description:** | **Unit:** | **Year:** | **Source:** |
| --- | --- | --- | --- | --- |
| HDI | Composite index of health (life expectancy),education (years of schooling) and wealth (gross national income per capita) | Index from 0 = least developed, to 1 = best developed | 2015 | World Bank |
| Governance | The average of six indicators that measure governance and that are all scaled in the same way: voice and accountability, political stability and absence of violence, government effectiveness, regulatory quality, rule of law, control of corruption | Index from -2.5 = poorest governance,to 2.5 = best governance | 2016 | World Bank |
| Country size | The land area of the country | Km2 | 2017 | Global Administrative Areas Database |
| % Terrestrial PAs | The fraction of land in each country that is covered by Protected Areas (PA) | percentage over the total land area | 2014 | World Bank |
| Rabies Incidence | Per capita death rate from rabies over 100,000 people | Death rate / 100,000 | 2015 | Hampson et al. 2015 |
| Rabies Costs | Total costs related to the burden of rabies impacts on the national public health sector budgets, local communities and livestock economies in USD in 2010 | USD | 2015 | Hampson et al. 2015 |

Data from the World Bank was retrieved from [www.govindicators.org](http://www.govindicators.org), whereas data from Global Administrative Areas Database was obtained from [www.gadm.org](http://www.gadm.org), and the rabies data from Hampson, K., Coudeville, L., Lembo, T., Sambo, M., Kieffer, A., Attlan, M., Barrat, J., Blanton, J.D., Briggs, D.J., Cleaveland, S., et al. (2015). Estimating the Global Burden of Endemic Canine Rabies. PLOS Neglected Tropical Diseases *9*, e0003709.

**Appendix S16:** The distribution of priority areas for vulture conservation identified through different conservation planning exercises including in turn the distributions of the 15 vulture species only (panel A), the distributions of the 15 vulture species and both poisoning threat layers (B), distributions of the 15 vulture species and wind threat layer (C), distributions of the 15 vulture species and Global Human Influence Index layer (D) across Africa-Eurasia. Grey areas depict places outside of the breeding and resident range of any of the 15 vultures considered. Priorities are ranked from highest (red color) to lowest (green).


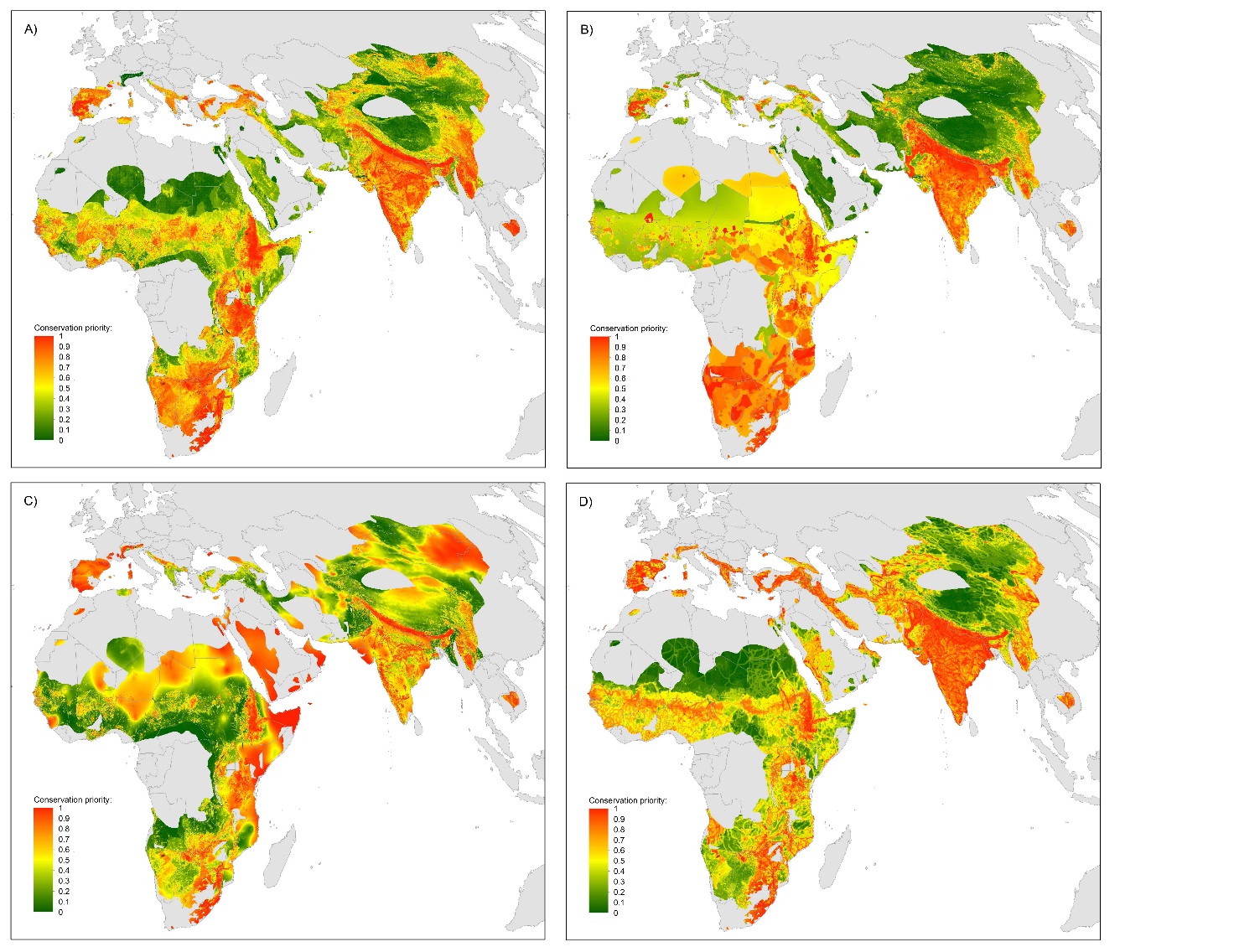


**Appendix S17:** The distribution of priority areas for vulture conservation identified as in Figure 1 in the main manuscript but taking in to account (technically penalising) areas of high uncertainty in the species distributions as well as the unintentional poisoning and wind layers (more details in Appendix S1). Figure A is based on priorities identified when 1 SD (standard deviation) was discounted from the species distributions, unintentional poisoning and wind layers to account for uncertainty, and Figure B when 0.5 SD was discounted. The Pearson correlation between each of the two maps shown here and the map showing the main results (Fig. 1) was r = 0.88 and 0.99 (correlation between Fig. 1 and Appendix S17A, and between Fig. 1 and Appendix S17B, respectively). This suggests that all these maps are rather concordant in identifying where priorities locate irrespective of the uncertainty associated to the features.


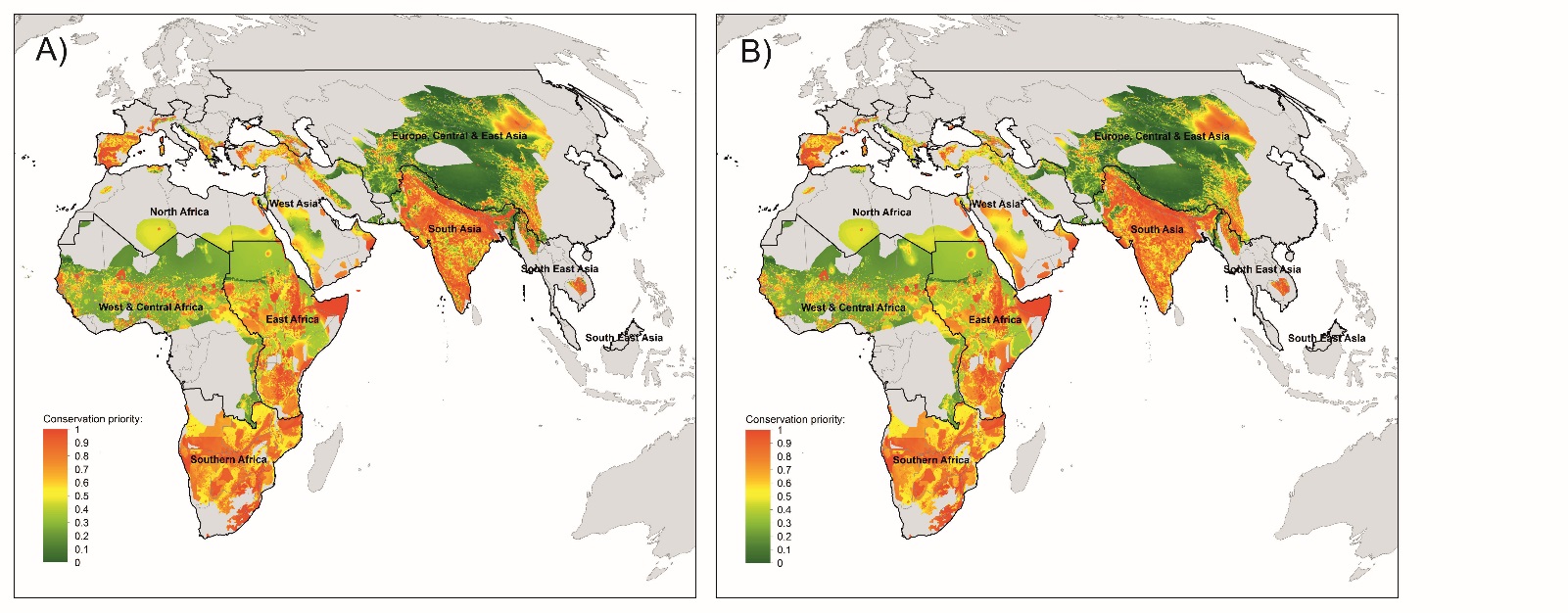


**Appendix S18:** The sensitivity of the prioritization results to weight changes assigned to the different features. The proportion of each feature retained in the top 30% fraction of the landscape (values and color gradient within each cell) when the weight of a given feature was varied (see x-axis, the numbers 2 and 4 after the feature name indicate that the weight of that feature was doubled or multiplied by four), while keeping the weights for all the other features constant (see Appendix S1). The baseline represents the situation whereby all features are weighted equally. The red colour indicates that a higher proportion a particular feature was retained in the final landscape. The Figure highlights an expected outcome, i.e. when the weight of a given feature is increased by 2 or 4 times, its representation in the top 30% fraction of the landscape consequently increases.


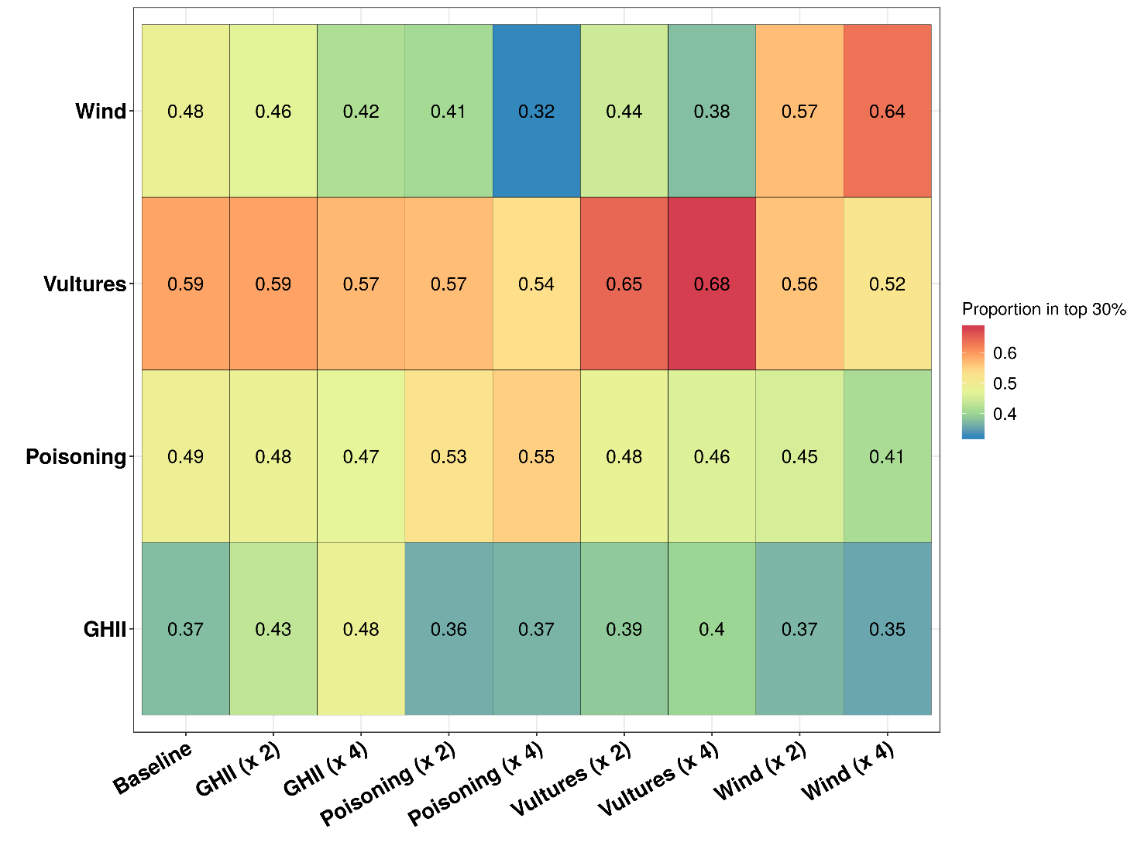

Supplement: Supplementary file 3 — Supporting Information [file COBI-33-1056-s003.docx]
